# Identification of Changes in Gene expression of rats after Sensory and Motor Nerves Injury

**DOI:** 10.1038/srep26579

**Published:** 2016-06-02

**Authors:** Yu Wang, Zhi-Yuan Guo, Xun Sun, Shi-bi Lu, Wen-Jing Xu, Qing Zhao, Jiang Peng

**Affiliations:** 1 Institute of Orthopedics, Chinese PLA General Hospital, FuXing Road 28^th^, Beijing, 100853, China; 2 Beijing Key Laboratory of Regenerative Medicine in Orthopedics, FuXing Road 28th, Beijing, 100853, China; 3 Key Laboratory of Musculoskeletal Trauma & War Injuries, PLA, FuXing Road 28th, Beijing, 100853, China; 4 The Neural Regeneration Co-innovation Center of Jiangsu Province, Qixiu Road 19th, Nantong, 226001, China; 5 Department of Orthopaedics, Cangzhou Central Hospital, Xinhua Road 16th, Cangzhou, 061001, China

## Abstract

Wallerian degeneration is a sequence of events in the distal stump of axotomized nerves. Despite large numbers of researches concentrating on WD, the biological mechanism still remains unclear. Hence we constructed a rat model with both motor and sensory nerves injury and then conducted a RNA-seq analysis. Here the rats were divided into the 4 following groups: normal motor nerves (NMN), injured motor nerves (IMN), normal sensory nerves (NSN) and injured sensory nerves (ISN). The transcriptomes of rats were sequenced by the Illumina HiSeq. The differentially expressed genes (DEGs) of 4 combinations including NMN vs. IMN, NSN vs. ISN, NMN vs. NSN and IMN vs. ISN were identified respectively. For the above 4 combinations, we identified 1666, 1514, 95 and 17 DEGs. We found that NMN vs. IMN shared the most common genes with NSN vs. ISN indicating common mechanisms between motor nerves injury and sensory nerves injury. At last, we performed an enrichment analysis and observed that the DEGs of NMN vs IMN and NSN vs. ISN were significantly associated with binding and activity, immune response, biosynthesis, metabolism and development. We hope our study may shed light on the molecular mechanisms of nerves degeneration and regeneration during WD.

Wallerian degeneration (WD) is a sequence of events in the distal stump of axotomized nerves, which begins with disintegration and degeneration of the axoplasma and axolemma^1^. Normally, we termed WD as the responses which was induced by axonal injury in the distal nerves segment^2^. The signals conducted by sensory nerves include pain and touch etc; motor nerves mainly participate in the control of muscle. Wallerian degeneration will occur in the distal end of injured nerves when peripheral nerves are damaged. Then, the damaged proximal nerves will grow towards to distal end, so that the injured nerves can be connected to regain nerves conduction function. However, the function of the nerves which is repaired after damaged is often not up to normal levels. One of the important factors leading to this phenomenon is the wrong connection of nerves.

In recent years, thousands of differentially expressed genes, the corresponding functional categories and signaling pathways have been identified^3^,^4^. Although the detailed molecular mechanisms of WD is still not well understood, previous studies indicated that it is a nervous system disease related with immune response^5^,^6^. For instance, some pro-inflammatory chemokines and cytokines, such as SOCS1 and SOCS3, have been showed to play important roles in the progress of WD^7^. Another research conducted by Lee *et al*.^8^ showed that Interleukin-6 (IL-6) was associated with WD. Despite of the rapid development of the mechanisms of WD, most of the current studies were performed by using microarrays or low-throughput experimental approaches without enough accuracy, throughput or sensitivity^9^.

In this study, we aimed to further investigate the detailed molecular mechanisms of WD. We built a rat model of WD which included both motor nerves injury and sensory nerves injury. Then the rats were divided into 4 groups: normal motor nerves group (NMN), normal sensory group (NSN), injured motor nerves group (IMN) and injured sensory nerves group (ISN). The four groups of rats formed 4 meaningful combinations including NMN vs. IMN, NSN vs. ISN, NMN vs. NSN and IMN vs. ISN. Then RNA-seq was exploited to get the transcriptome sequences of rats and differentially expressed genes of each combination were identified. At last, we determined the functional categories and KEGG pathways where differentially expressed genes were annotated by performing enrichment analyses. We hope our study may shed light on the molecular mechanisms of nerves degeneration and regeneration during WD.

## Results

### RNA Sequencing and Quality Analysis

The four groups can be abbreviated to NMN (normal motor nerves), IMN (injured motor nerves), NSN (normal sensory nerves) and ISN (injured sensory nerves). The A260/280 ratio for each sample is higher than 1.9 and the A260/230 ratio for each sample is higher than 2.0, suggesting the mRNA samples are of high quality. RIN values for our total RNA extractions ranged from 9.2 to 9.4, a measure of high quality. For each of the RNA samples, the paired-end cDNA library was prepared and RNA-seq was performed using the Illumina HiSeq. Across the samples, the number of raw reads ranged from 11,741,496 to 12,669,039. The detailed information for sequencing quality can be found in section 1 of Supplementary File. No significant difference was observed in the number of the reads between the control group and injured group (Student’s t-test: p = 0.27). After quality control, we calculated the percentage of clean reads from the total mapped reads and found that the percent of clean reads reached up to 99% for each of the samples. To assess the quality of reads, we mapped these reads to the reference genome of rat using SOAP. The proportion of total clean reads mapped to reference genome ranged from 76.51% to 90.07% (details see Table 1), and the percent of clean reads mapped to reference genes was from 61.52% to 73.70%. The above results demonstrated that the reads mapped to the reference genome quite well and our data was highly reliable.

### Differentially Expressed Genes (DEGs)

For the 4 groups (NMN, IMN, NSN, and ISN) of rats, we created 4 combinations including NMN vs. IMN, NSN vs. ISN, NMN vs. NSN and IMN vs. ISN. Then differentially expressed genes for each combination were identified (Table 2). There were 1666 DEGs of which 920 genes were up-regulated and the remaining 746 were down-regulated before and after motor nerves injury (NMN vs. IMN). 1514 DEGs including 763 up-regulated genes and 751 down-regulated genes were identified before and after sensory nerves injury (NSN vs. ISN). We identified 95 (18 up-regulated genes an 77 down-regulated genes) and 17 DEGs (2 up-regulated genes and 15 down-regulated genes) respectively for the group of normal motor nerves against normal sensory nerves (NMN vs. NSN) and the group of injured motor nerves against injured sensory nerves (IMN vs. ISN). The detailed information of DEGs for 4 combinations are listed from Table S9 to Table S12 at Section 3 of the Supplementary File.

**Figure 1:**
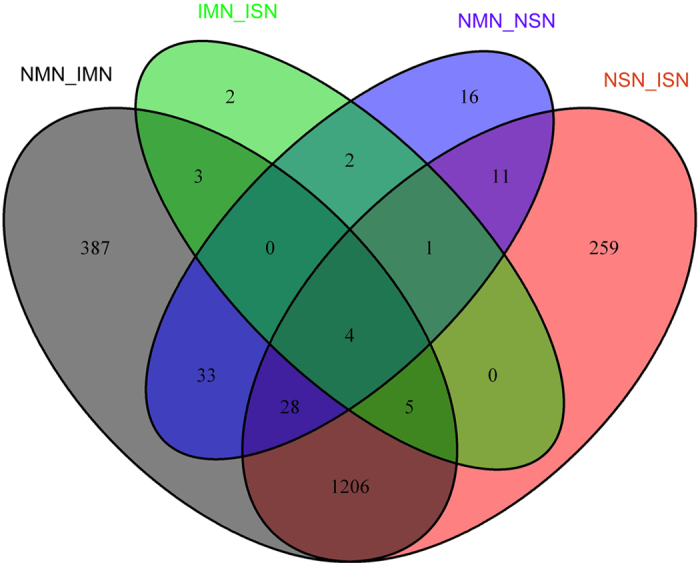
The intersection among the 4 groups of DEGs which include NMN vs. IMN, NSN vs. ISN, NMN vs. NSN and IMN vs. ISN.

**Figure 2:**
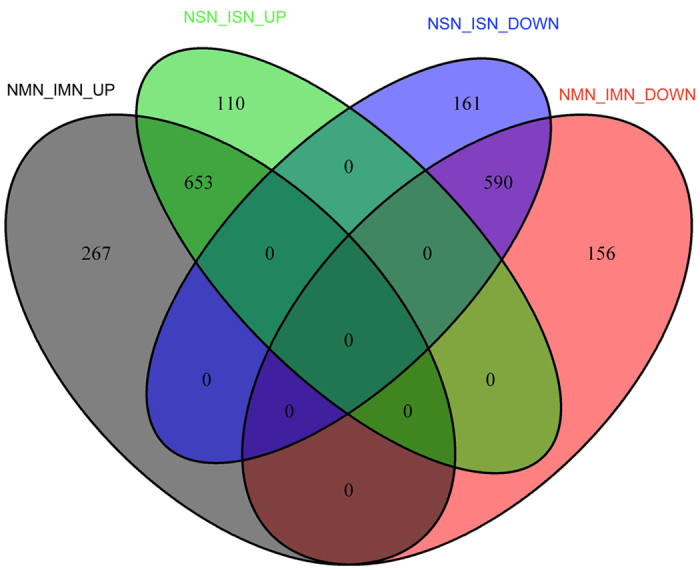
The intersection between up-regulated genes and down-regulated genes for the group of NMN vs. IMN and NSN vs. ISN.

To further explore the relationship of DEGs among the 4 combinations, we computed the number of common genes shared by the 4 groups and then displayed the intersection by plotting a Venn diagram (Fig. 1). 1243 common genes were shared between NMN vs. IMN and NSN vs. ISN. These common genes accounted for a large proportion in both the group of NMN vs. IMN (74.6%) and NSN vs. ISN (82.1%), which possibly indicated the common mechanisms between motor nerves injury and sensory nerves injury. Next, we investigated the intersection relationship of up-regulated and down-regulated genes between NMN vs. IMN and NSN vs. ISN (Fig. 2). We found 653 common genes between up-regulated genes of NMN vs. IMN and up-regulated genes of NSN vs. ISN, and found 590 common genes shared by down-regulated NSN vs. ISN and down-regulated NMN vs. IMN. However, no common genes were observed between any of the up-regulated groups and down-regulated groups.

### Gene Ontology Functional Classification of DEGs

For the sake of enhancing the understanding of molecular mechanisms of WD, we performed a GO annotation analysis using DAVID software for each of the 4 groups of DEGs. The DEGs of both NMN vs. IMN and NSN vs. ISN were mainly annotated to the molecular functions of binding and activity, such as protein binding, pattern binding, growth factor binding, transferase activity and transporter activity. For the groups of NMN vs. IMN and NSN vs. ISN, the up-regulated genes were significantly enriched in biological processes associated with immune response including response to stress, response to external stimulus, response to wounding and regulation of immune system process. However the down-regulated genes of both NMN vs. IMN and NSN vs. ISN were mainly associated with biosynthetic process, metabolic process and developmental process. The DEGs of normal motor nerves against normal sensory nerves (NMN vs. NSN) were significantly annotated to biological processes associated involved in development which included multicellular organismal development, anatomical structure development, system development and tissue development. The DEGs of injured motor nerves against injured sensory nerves (IMN vs. ISN) were mainly associated with inflammatory response, positive regulation of cellular development. Detailed annotation results can be found at Section 2 of Supplementary File (from Table S1 to Table S8).

### KEGG Pathway Enrichment Analysis of DEGs

We also analyzed the biological pathways of 4 groups of DEGs. For the group of NMN vs. IMN, the up-regulated genes were mainly annotated to pathways of cell cycle, p53 signaling pathway and DNA replication, while the down-regulated genes mainly enriched for biosynthesis and metabolic pathways such as steroid biosynthesis, terpenoid backbone biosynthesis, amino acid biosynthesis, drug metabolism and pyruvate metabolism. The KEGG pathway results of NSN vs. ISN group were consistent with those of NMN vs. IMN. For the DEGs of normal motor nerves against normal sensory nerves and injured motor nerves against injured sensory nerves, KEGG pathway enrichment analyses were also performed and detailed results can be found at section 2 of Supplementary File.

## Discussion

To deeply understand the molecular mechanisms of WD, we built a rat model with both motor and sensory nerves injury and used the RNA-seq technology to obtain the sequence of rat transcriptome. In this study, we not only focused on the DEGs between normal motor (sensory) nerves and injured motor (sensory) nerves, but also identified the DEGs between normal (injured) motor nerves and normal (injured) sensory nerves. Therefore, we got 4 groups of DEGs and then analyzed these DEGs from multiple aspects. Meanwhile, the GO and KEGG enrichment analysis was performed for each of the 4 groups of DEGs respectively.

Despite the large numbers of researches focusing on WD, most of them are based on microarrays technology. Compared with microarrays technology, RNA-seq has several advantages including higher sensitivity, wider linkage range of expression level and higher correlation coefficient^10^. More importantly, a large amount of data can be obtained through RNA-seq which makes it available to compare the expression level between two samples^11^. Furthermore, the RPKM is the most reliable approach for measuring the expression values of genes. The normalization was performed for both the sequencing depth and gene length that makes the expression values of genes with different length or under different sequencing depth can be comparable^12^. The RPKM values can be directly used to assess the expression difference between different samples. In brief, RNA-seq technology provides a powerful tool to find the changes in gene expression between two or more conditions^13^,^14^,^15^,^16^,^17^. Therefore, the data of high quality lay the foundation for the subsequent analysis.

It is worth noting that we used the NOIseq method^18^ to identify DEGs. Compared with NOISeq, the most majority of other approaches such as edgeR^19^, DESeq^20^, SAMseq^21^ and baySeq^22^ are strongly dependent on the sequencing depth. Thus the false positive rate (FPR) will be increased along with the increased number of reads. However, for NOISeq, the FPR remains at stable level. In addition, the noise distribution model of NOISeq is constructed based on real data, which makes it suitable for different data with various data size and perfectly controls false discovery rate (FDR)^23^.

Through analyzing the features of DEGs, we found that the group of NMN vs. IMN shared large amounts of genes with the group of NSN vs. ISN which indicated tight connection between the mechanism of motor nerves injury and sensory nerves injury. Then we further investigated the relationship of up-regulated and down-regulated genes before and after motor (sensory) nerves injury. Interestingly, we observed a phenomenon that there was a large overlap between up-regulated genes of NMN vs. IMN and up-regulated genes of NSN vs. ISN, however, no overlap was found between the up-regulated genes and down-regulated genes for both the 4 possible combinations. These results suggest that the gene expression change caused by motor nerves injury is consistent with that due to sensory nerves injury.

Although the sample size of our study is not very large, RNA-seq provides us sufficient data for identifying DEGs and further analyzing their features. In this research, we tried to explore the biological mechanisms underlying the WD and hoped that our results would provide new insights for the following studies of WD in the future.

## Materials and Methods

### Construction of Animal Model

16 male adult wistar rats whose weights ranged from 200 to 250 g were provided by the Animal Center of PLA General Hospital. All experimental procedures were approved by the Institutional Ethics Committee, Chinese PLA General Hospital in China and were carried out in accordance with relevant guidelines and regulations. Rats were anaesthetized by using 10% chloral hydrate intraperitoneal injection (0.3 ml/100 g) which was used to keep the rat quiet and 1% lidocaine local infiltration which was used to relieve the pain during operation procedure. Rats were housed in a temperature and humidity controlled room with a 12-hours light/12-hours dark cycle and allowed free access to food and water. The cages were bedded with clean and dried sawdust. The rats were randomly divided into 2 groups (n = 8) to undergo nerve transection and to do a biological repeat. The right hind limb of each rat were as the experimental group and the left side as the control group. We separated the right femoral nerves carefully, then transected at about 5 mm above its bifurcation and ligated with thin line at the distal of nerves. Meanwhile, we did nothing to the control group. Seven days later, the animals were anaesthetized by using 10% chloral hydrate intraperitoneal injection and scacrificed by cervical vertebral dislocation. The length of muscle branch sample was about 1 centimeter and the saphenous nerve was about 2 centimeters. After getting the samples, we placed it into the cold sterile saline quickly to wash the blood, and then immediately frozen in liquid nitrogen. The samples saved in −80 °C refrigerator until use. For the following analysis, these samples were divided into 4 groups which included normal motor nerves group (NMN), injured motor nerves group (IMN), normal sensory nerves group (NSN) and injured sensory nerves group (ISN).

### Transcriptome Sequencing

The RNA purity and integrity were calculated to measure the quality of mRNA samples prior to fragmentation and sequencing. The RNA concentration was measured by the absorbance at 230, 260 and 280 nm by using a spectrophotometer. The purity of RNA was estimated by calculating the ratios of A_260_/A_280_ and A_260_/A_230_ to evaluate the levels of protein and polysaccharide/phenolic compound contamination, respectively. RNA integrity was evaluated by a RNA Integrity Number (RIN) based on the comparison of the areas of 18S rRNA and 28 s rRNA^24^,^25^. RIN values range from 1 indicating the most degraded to 10 indicating the most intact. After mRNA quality evaluation, the total RNAs were first treated with DNase I to degrade any possible DNA contamination after extracting from samples. Then the mRNAs were enriched by using the oligo (dT) magnetic beads. By using the fragmentation buffer, the mRNAs were fragmented into short fragments (about 200 bp). Then the paired-end cDNA libraries were prepared for each sample and sequenced by Illumina HiSeq^TM^ 2000. By base calling, the original image data produced by the sequencer was transferred into sequences, which were saved as FASTQ files. Due to the low quality reads or adaptor sequences contained in the raw data, sequence quality controls were performed before the further analysis and those reads satisfying the quality controls were thought as “clean reads”. The quality control process was carried out with the following three criteria: (1) removal of the reads that contained adaptors; (2) removal of the reads with more than 10% N-bases and (3) removal of the low quality reads in which the number of Q ≤ 5 bases accounted for more than 50% of the total reads.

### Read Mapping and Quantification

Clean reads for each sample were mapped to the UCSC Rattus norregicus reference genome (RGSC 6.0) using SOAP2^26^ with no more than 2 mismatches. The expression value of each gene was calculated based on the numbers of reads uniquely mapped to the specific gene and the total number of uniquely mapped reads in the sample. We calculated the gene expression value by using RPKM method^12^ (Reads Per kb per Million reads). The formula of this method was described as follows:


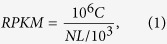


of which RPKM is the expression value of a gene; C is the number of reads that were uniquely mapped to the gene; N is the total number of reads that were uniquely aligned to all genes and L is the number of bases of a gene.

### Differentially Expressed Genes (DEGs)

To identify the differentially expressed genes between different sample groups, we used the NOIseq^18^ method which was a non-parametric approach for differential expression analyis of RNA-seq data. The basic idea of NOIseq is to compare the expression change between two conditions and to determine whether the expression change is significantly greater than the change found within the same experiment condition. The identification of DEGs was performed by Bioconductor R packages named NOISeq^27^.

### GO (Gene Ontology) and KEGG Pathway enrichment analysis of DEGs

The DAVID^28^ software, a commonly used web based tool, was utilized to conduct the GO and KEGG pathway enrichment. The enrichment analysis was based on hypergeometric test with the following formula:


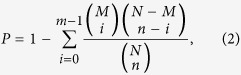


where N is the total number of background genes, n is the number of DEGs, M is the number of genes contained in a certain GO term and m is the number of DEGs in M. A GO term or pathway will be determined as significantly enriched when the p value is less than 0.05. In this study, we provided all GO terms and pathways significantly which were enriched with DEGs when compared to the genome background.

## Additional Information

**How to cite this article**: Wang, Y. *et al*. Identification of Changes in Gene expression of rats after Sensory and Motor Nerves Injury. *Sci. Rep*. **6**, 26579; doi: 10.1038/srep26579 (2016).

## Supplementary Material

Supplementary Information

## Figures and Tables

**Table 1:** The number of sequence reads mapping to the rat reference genome.

Sample ID	Raw Reads	Clean Reads (Percentage %)	Mapped Reads (Percentage %)
IMN1	12,001,477	11,933,482(99.43)	10,684,534(89.53)
IMN2	12,297,951	12,237,412(99.51)	11,022,600(90.07)
NMN1	12,679,815	12,615,224(99.49)	11,276,867(89.39)
NMN2	12,419,793	12,354,481(99.47)	9,452,604(76.51)
ISN1	11,822,467	11,765,703(99.52)	10,416,092(88.53)
ISN2	12,089,195	12,023,341(99.46)	10,719,873(89.16)
NSN1	11,741,496	11,682,257(99.50)	10,400,091(89.02)
NSN2	12,669,039	12,611,011(99.54)	11,290,287(89.53)

**Table 2:** The number of differentially expressed genes for 4 combinations.

Groups	Number of DEGs	Number of up-regulated DEGs	Number of down-regulated DEGs
NMN vs. IMN	1666	920	746
NSN vs. ISN	1514	763	751
NMN vs. NSN	95	18	77
IMN vs. ISN	17	2	15
