# Supplementary material for: Identification of Changes in Gene expression of rats after Sensory and Motor Nerves Injury
Source: Sci Rep. 2016 Jun 2;6:26579. doi: 10.1038/srep26579 (PMC4890583; doi:10.1038/srep26579)
Supplement: Supplementary Information [file srep26579-s1.pdf]

Identification of Changes in Gene expression of rats after Sensory and Motor Nerves Injury

Yu Wang[†,1], Zhi-Yuan Guo[†,1], Xun Sun[1], Shi-bi Lu[1], Wen-Jing Xu[1], Qing Zhao[*,2], Jiang Peng[*,1]

[1] Institute of Orthopedics, Chinese PLA General Hospital, FuXing Road 28th, Beijing, 100853, China

[2] Department of Orthopaedics, First Affiliated Hospital of PLA General Hospital, No. 51, Fucheng Road, Beijing, China.

* To whom correspondence should be addressed: pengjiang_beijing@163.com (Jiang Peng) and zhaoqing_beijing@163.com (Qing Zhao)

† Joint First Authors.

Section 1

Quality assessment of sequencing

1. Statistics of alignment

Table 1. Summary of mapping result (mapping to reference genes)

Sample ID	Total Reads	Total BasePairs	Total Mapped Reads	Perfect Match	<=2bp Mismatch	Unique Match	Multi-position Match	Total Unmapped Reads
MB1	11,933,482 (100.00%)	584,740,618 (100.00%)	6,300,735 (52.80%)	5,348,185 (44.82%)	952,550 (7.98%)	5,866,833 (49.16%)	433,902 (3.64%)	5,632,747 (47.20%)
MB2	12,237,412 (100.00%)	599,633,188 (100.00%)	6,642,934 (54.28%)	5,595,128 (45.72%)	1,047,806 (8.56%)	6,162,096 (50.35%)	480,838 (3.93%)	5,594,478 (45.72%)
MN1	12,615,224 (100.00%)	618,145,976 (100.00%)	6,715,542 (53.23%)	5,668,862 (44.94%)	1,046,680 (8.30%)	6,205,782 (49.19%)	509,760 (4.04%)	5,899,682 (46.77%)
MN2	12,354,481 (100.00%)	605,369,569 (100.00%)	5,602,973 (45.35%)	4,650,198 (37.64%)	952,775 (7.71%)	5,177,031 (41.90%)	425,942 (3.45%)	6,751,508 (54.65%)
SB1	11,765,703 (100.00%)	576,519,447 (100.00%)	7,117,232 (60.49%)	6,032,208 (51.27%)	1,085,024 (9.22%)	6,629,040 (56.34%)	488,192 (4.15%)	4,648,471 (39.51%)
SB2	12,023,341 (100.00%)	589,143,709 (100.00%)	6,655,252 (55.35%)	5,669,129 (47.15%)	986,123 (8.20%)	6,199,205 (51.56%)	456,047 (3.79%)	5,368,089 (44.65%)
SN1	11,682,257 (100.00%)	572,430,593 (100.00%)	6,649,271 (56.92%)	5,585,177 (47.81%)	1,064,094 (9.11%)	6,106,653 (52.27%)	542,618 (4.64%)	5,032,986 (43.08%)
SN2	12,611,011 (100.00%)	617,939,539 (100.00%)	6,525,960 (51.75%)	5,541,667 (43.94%)	984,293 (7.81%)	6,036,823 (47.87%)	489,137 (3.88%)	6,085,051 (48.25%)

Table 1. Summary of mapping result (mapping to reference genome)

Sample ID	Total Reads	Total BasePairs	Total Mapped Reads	Perfect Match	<=3bp Mismatch	Unique Match	Multi-position Match	Total Unmapped Reads
MB1	11,933,482 (100.00%)	584,740,618 (100.00%)	10,684,534 (89.53%)	8,717,992 (73.05%)	1,966,542 (16.48%)	9,542,456 (79.96%)	1,142,078 (9.57%)	1,248,948 (10.47%)
MB2	12,237,412 (100.00%)	599,633,188 (100.00%)	11,022,600 (90.07%)	8,869,420 (72.48%)	2,153,180 (17.60%)	9,773,119 (79.86%)	1,249,481 (10.21%)	1,214,812 (9.93%)
MN1	12,615,224 (100.00%)	618,145,976 (100.00%)	11,276,867 (89.39%)	9,247,911 (73.31%)	2,028,956 (16.08%)	10,223,994 (81.04%)	1,052,873 (8.35%)	1,338,357 (10.61%)
MN2	12,354,481 (100.00%)	605,369,569 (100.00%)	9,452,604 (76.51%)	7,601,007 (61.52%)	1,851,597 (14.99%)	8,541,808 (69.14%)	910,796 (7.37%)	2,901,877 (23.49%)
SB1	11,765,703 (100.00%)	576,519,447 (100.00%)	10,416,092 (88.53%)	8,460,030 (71.90%)	1,956,062 (16.63%)	9,237,800 (78.51%)	1,178,292 (10.01%)	1,349,611 (11.47%)
SB2	12,023,341 (100.00%)	589,143,709 (100.00%)	10,719,873 (89.16%)	8,800,950 (73.20%)	1,918,923 (15.96%)	9,571,788 (79.61%)	1,148,085 (9.55%)	1,303,468 (10.84%)
SN1	11,682,257 (100.00%)	572,430,593 (100.00%)	10,400,091 (89.02%)	8,500,359 (72.76%)	1,899,732 (16.26%)	9,409,355 (80.54%)	990,736 (8.48%)	1,282,166 (10.98%)
SN2	12,611,011 (100.00%)	617,939,539 (100.00%)	11,290,287 (89.53%)	9,294,021 (73.70%)	1,996,266 (15.83%)	10,238,042 (81.18%)	1,052,245 (8.34%)	1,320,724 (10.47%)

2. Quality assessment of reads

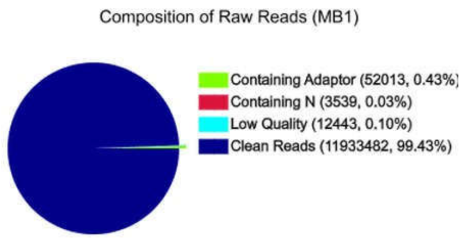

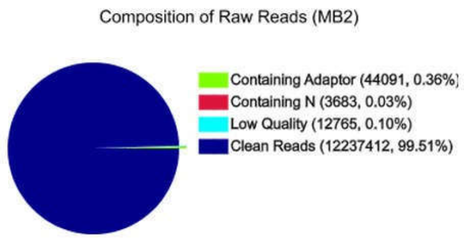

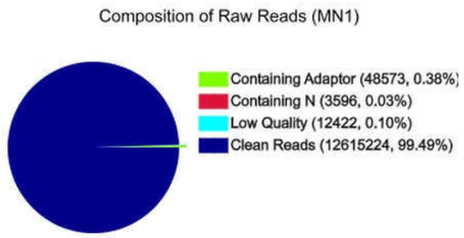

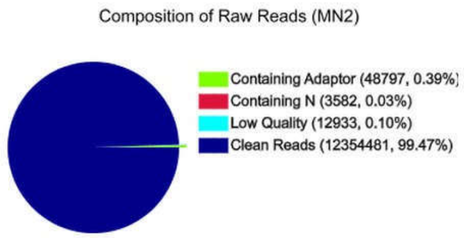

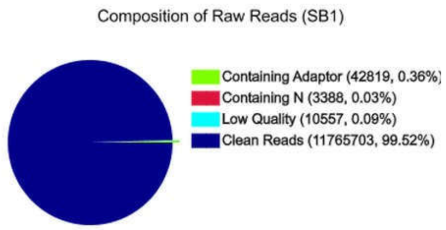

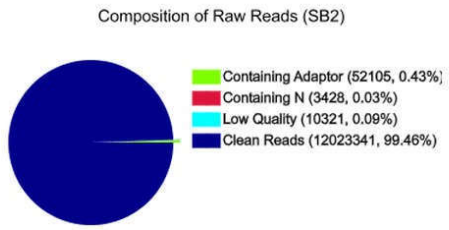

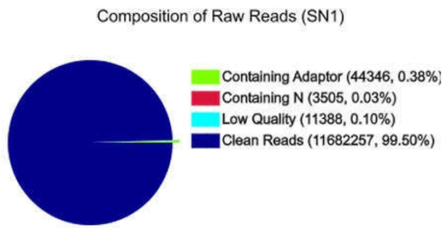

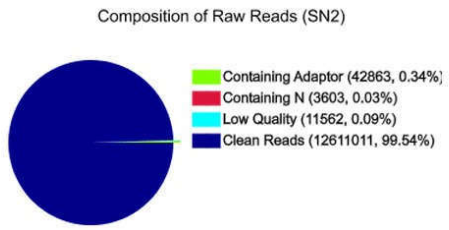

3. Sequencing saturation analysis

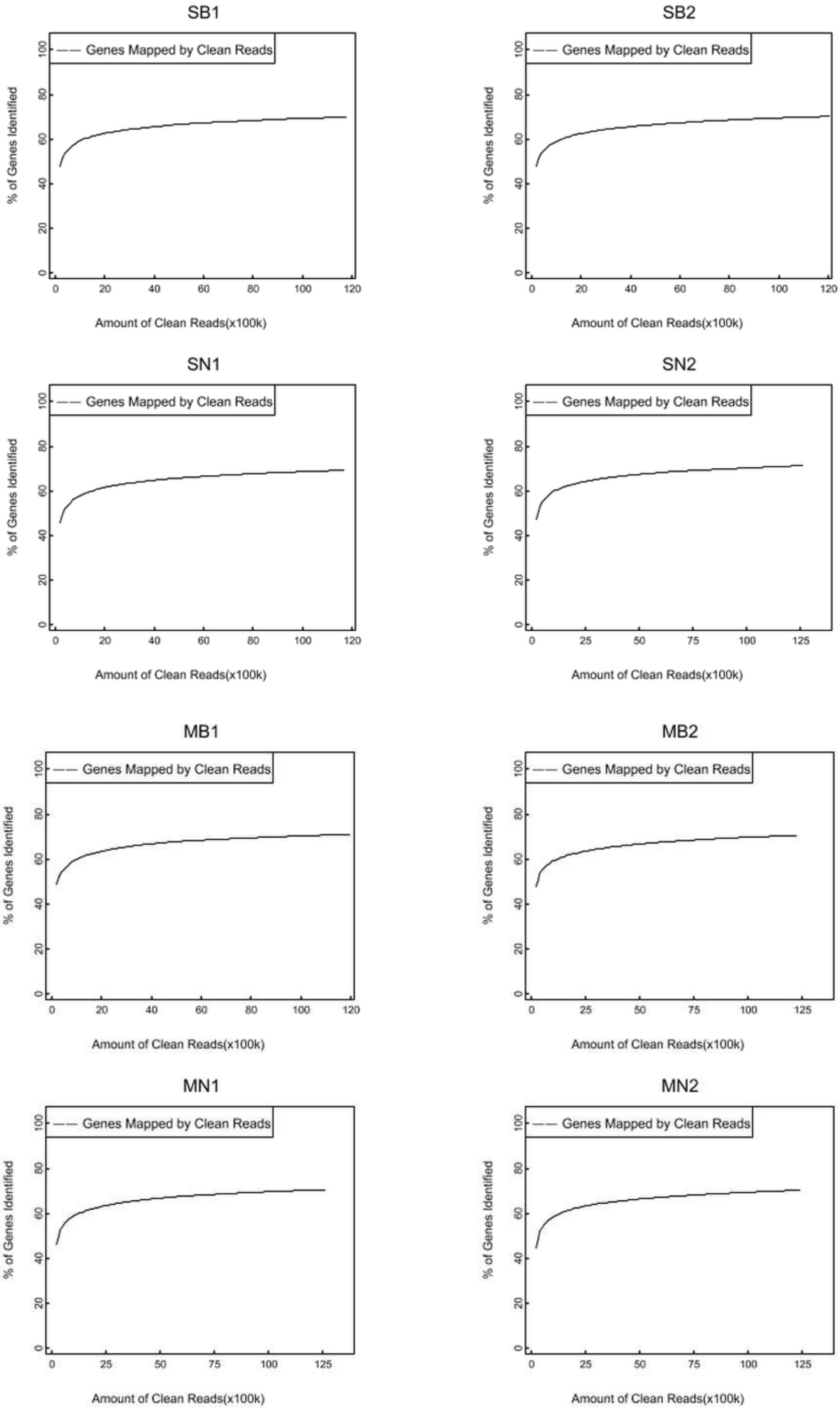

4. Randomness assessment

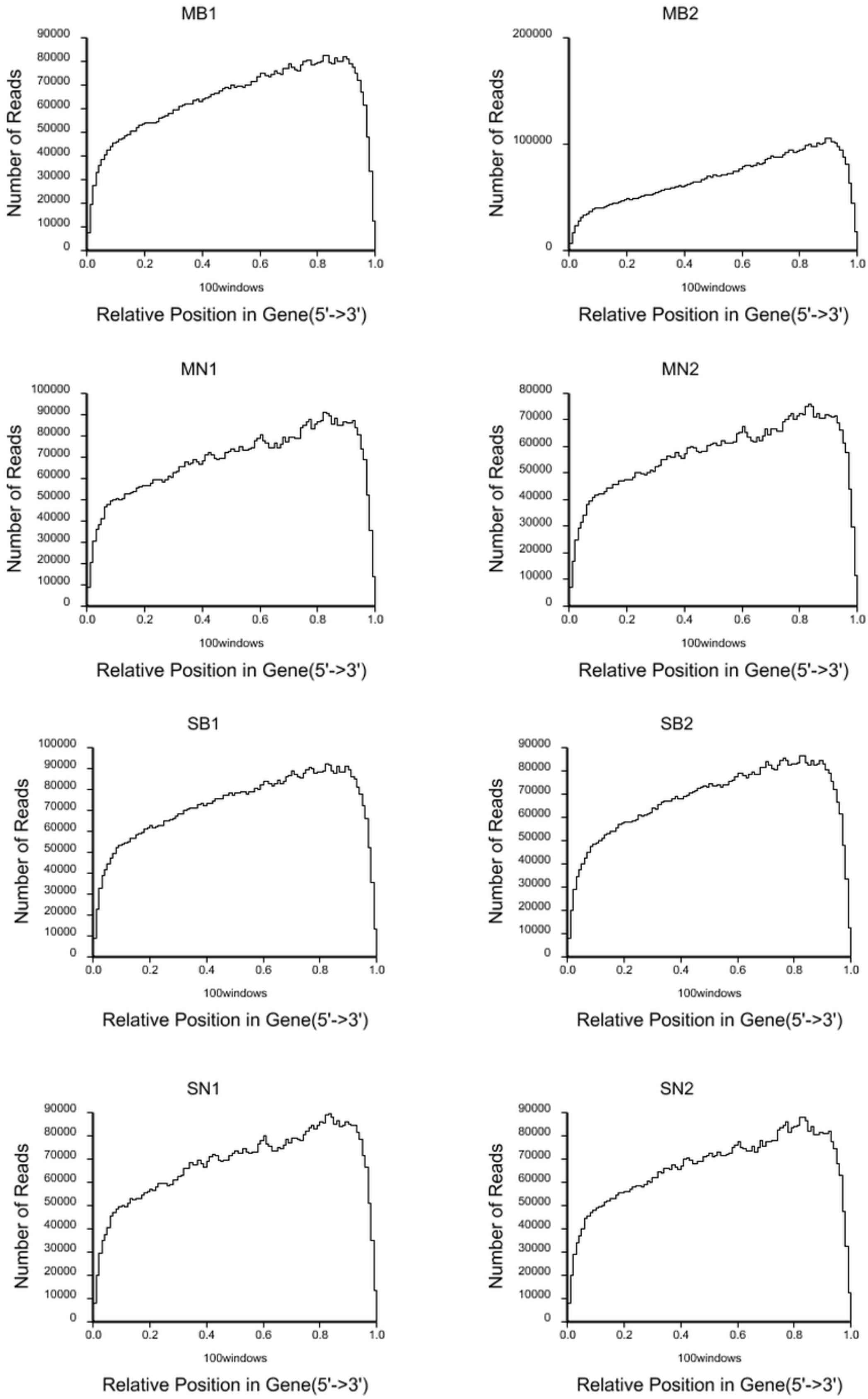

5. Distribution of reads on reference genome

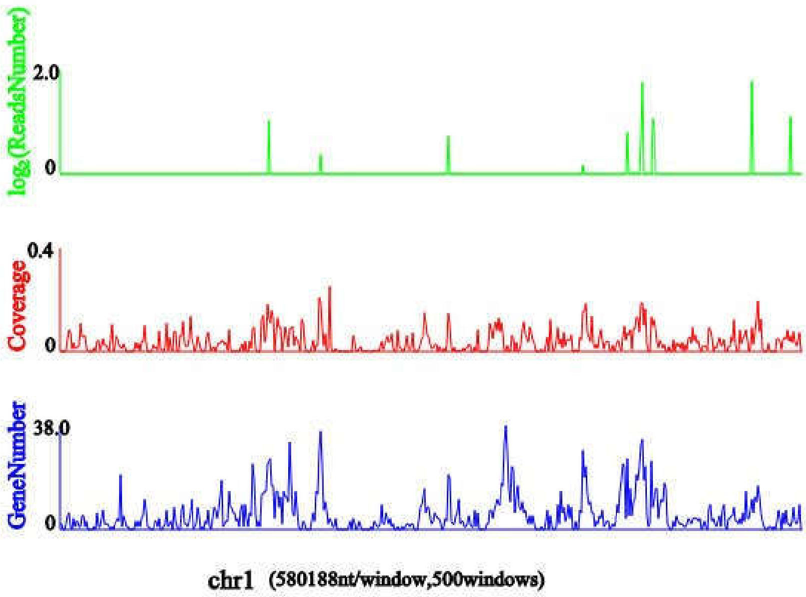

Section 2

Identification of Changes in Gene expression of rats after Sensory and Motor Nerves Injury

--Supplementary information: supplementary files of GO and KEGG annotation results

1. NMN vs. IMN

Table S1. The biological processes (BP) in which DEGs are enriched.

Category	Term	Count	%	PValue	FDR
GOTERM_BP_ALL	GO:0032502~developmental process	387	25.37704918	4.27E-29	7.99E-26
GOTERM_BP_ALL	GO:0048856~anatomical structure development	339	22.2295082	3.26E-28	6.09E-25
GOTERM_BP_ALL	GO:0048731~system development	322	21.1147541	6.41E-27	1.20E-23
GOTERM_BP_ALL	GO:0007275~multicellular organismal development	350	22.95081967	6.72E-26	1.26E-22
GOTERM_BP_ALL	GO:0009987~cellular process	832	54.55737705	1.08E-22	2.01E-19
GOTERM_BP_ALL	GO:0010033~response to organic substance	169	11.08196721	2.93E-20	5.48E-17
GOTERM_BP_ALL	GO:0009725~response to hormone stimulus	112	7.344262295	1.42E-19	2.65E-16
GOTERM_BP_ALL	GO:0009719~response to endogenous stimulus	120	7.868852459	3.33E-19	6.22E-16
GOTERM_BP_ALL	GO:0042127~regulation of cell proliferation	131	8.590163934	1.39E-18	2.60E-15

GOTERM_BP_ALL	GO:0048545~response to steroid hormone stimulus	76	4.983606557	8.62E-18	1.61E-14
GOTERM_BP_ALL	GO:0048513~organ development	244	16	9.40E-18	1.76E-14
GOTERM_BP_ALL	GO:0009605~response to external stimulus	147	9.639344262	3.05E-17	5.70E-14
GOTERM_BP_ALL	GO:0048869~cellular developmental process	229	15.01639344	3.99E-16	8.33E-13
GOTERM_BP_ALL	GO:0030154~cell differentiation	222	14.55737705	4.91E-16	8.33E-13
GOTERM_BP_ALL	GO:0065007~biological regulation	614	40.26229508	5.90E-16	1.04E-12
GOTERM_BP_ALL	GO:0009611~response to wounding	91	5.967213115	3.36E-15	6.23E-12
GOTERM_BP_ALL	GO:0016043~cellular component organization	268	17.57377049	1.03E-14	1.93E-11
GOTERM_BP_ALL	GO:0006950~response to stress	206	13.50819672	2.01E-14	3.76E-11
GOTERM_BP_ALL	GO:0006629~lipid metabolic process	121	7.93442623	3.51E-14	6.56E-11
GOTERM_BP_ALL	GO:0051239~regulation of multicellular organismal process	160	10.49180328	4.19E-14	7.83E-11
GOTERM_BP_ALL	GO:0050793~regulation of developmental process	123	8.06557377	6.15E-14	1.15E-10
GOTERM_BP_ALL	GO:0051128~regulation of cellular component organization	84	5.508196721	1.21E-13	2.26E-10
GOTERM_BP_ALL	GO:0008610~lipid biosynthetic process	67	4.393442623	1.39E-13	2.61E-10
GOTERM_BP_ALL	GO:0048518~positive regulation of biological process	256	16.78688525	1.87E-13	3.50E-10
GOTERM_BP_ALL	GO:0007399~nervous system development	157	10.29508197	2.28E-13	4.26E-10
GOTERM_BP_ALL	GO:0048522~positive regulation of cellular process	234	15.3442623	6.07E-13	1.14E-09
GOTERM_BP_ALL	GO:0006695~cholesterol biosynthetic process	18	1.180327869	7.35E-13	1.38E-09
GOTERM_BP_ALL	GO:0050789~regulation of biological process	563	36.91803279	1.07E-12	2.00E-09
GOTERM_BP_ALL	GO:0016125~sterol metabolic process	31	2.032786885	1.26E-12	2.36E-09
GOTERM_BP_ALL	GO:0022603~regulation of anatomical structure morphogenesis	58	3.803278689	1.63E-12	3.04E-09
GOTERM_BP_ALL	GO:0065008~regulation of biological quality	195	12.78688525	2.46E-12	4.61E-09
GOTERM_BP_ALL	GO:0016126~sterol biosynthetic process	19	1.245901639	3.66E-12	6.84E-09
GOTERM_BP_ALL	GO:0031960~response to corticosteroid stimulus	40	2.62295082	7.17E-12	1.34E-08
GOTERM_BP_ALL	GO:0043062~extracellular structure organization	41	2.68852459	9.86E-12	1.84E-08
GOTERM_BP_ALL	GO:0009653~anatomical structure morphogenesis	163	10.68852459	2.11E-11	3.94E-08
GOTERM_BP_ALL	GO:0051384~response to glucocorticoid stimulus	38	2.491803279	2.30E-11	4.31E-08
GOTERM_BP_ALL	GO:0001568~blood vessel development	55	3.606557377	2.45E-11	4.58E-08
GOTERM_BP_ALL	GO:0001944~vasculature development	56	3.672131148	2.59E-11	4.85E-08
GOTERM_BP_ALL	GO:0008203~cholesterol metabolic process	28	1.836065574	3.37E-11	6.31E-08
GOTERM_BP_ALL	GO:0009888~tissue development	107	7.016393443	4.76E-11	8.90E-08
GOTERM_BP_ALL	GO:0007154~cell communication	92	6.032786885	1.36E-10	2.55E-07
GOTERM_BP_ALL	GO:0048519~negative regulation of biological process	215	14.09836066	1.55E-10	2.90E-07
GOTERM_BP_ALL	GO:0008284~positive regulation of cell proliferation	75	4.918032787	2.31E-10	4.32E-07
GOTERM_BP_ALL	GO:0048523~negative regulation of cellular process	198	12.98360656	3.25E-10	6.09E-07
GOTERM_BP_ALL	GO:0030198~extracellular matrix organization	29	1.901639344	1.22E-09	2.29E-06
GOTERM_BP_ALL	GO:0042060~wound healing	43	2.819672131	1.67E-09	3.12E-06
GOTERM_BP_ALL	GO:0050794~regulation of cellular process	516	33.83606557	1.72E-09	3.21E-06
GOTERM_BP_ALL	GO:0006694~steroid biosynthetic process	26	1.704918033	2.32E-09	4.34E-06
GOTERM_BP_ALL	GO:0032879~regulation of localization	102	6.68852459	2.43E-09	4.54E-06
GOTERM_BP_ALL	GO:0048514~blood vessel morphogenesis	44	2.885245902	4.40E-09	8.23E-06

GOTERM_BP_ALL	GO:0044255~cellular lipid metabolic process	83	5.442622951	4.44E-09	8.31E-06
GOTERM_BP_ALL	GO:0022604~regulation of cell morphogenesis	35	2.295081967	5.44E-09	1.02E-05
GOTERM_BP_ALL	GO:0008202~steroid metabolic process	41	2.68852459	6.21E-09	1.16E-05
GOTERM_BP_ALL	GO:0045595~regulation of cell differentiation	86	5.639344262	7.79E-09	1.46E-05
GOTERM_BP_ALL	GO:0070482~response to oxygen levels	46	3.016393443	7.79E-09	1.46E-05
GOTERM_BP_ALL	GO:0048468~cell development	104	6.819672131	1.66E-08	3.10E-05
GOTERM_BP_ALL	GO:0031099~regeneration	32	2.098360656	2.24E-08	4.18E-05
GOTERM_BP_ALL	GO:0022610~biological adhesion	79	5.180327869	2.67E-08	5.00E-05
GOTERM_BP_ALL	GO:0007155~cell adhesion	79	5.180327869	2.67E-08	5.00E-05
GOTERM_BP_ALL	GO:0022403~cell cycle phase	49	3.213114754	2.99E-08	5.59E-05
GOTERM_BP_ALL	GO:0051093~negative regulation of developmental process	52	3.409836066	3.08E-08	5.77E-05
GOTERM_BP_ALL	GO:0007049~cell cycle	78	5.114754098	4.35E-08	8.14E-05
GOTERM_BP_ALL	GO:0042493~response to drug	61	4	5.26E-08	9.85E-05
GOTERM_BP_ALL	GO:0006066~alcohol metabolic process	70	4.590163934	6.10E-08	1.14E-04
GOTERM_BP_ALL	GO:0007272~ensheathment of neurons	19	1.245901639	6.47E-08	1.21E-04
GOTERM_BP_ALL	GO:0008366~axon ensheathment	19	1.245901639	6.47E-08	1.21E-04
GOTERM_BP_ALL	GO:0045596~negative regulation of cell differentiation	45	2.950819672	8.95E-08	1.67E-04
GOTERM_BP_ALL	GO:0008285~negative regulation of cell proliferation	52	3.409836066	9.93E-08	1.86E-04
GOTERM_BP_ALL	GO:0009991~response to extracellular stimulus	57	3.737704918	1.02E-07	1.90E-04
GOTERM_BP_ALL	GO:0002682~regulation of immune system process	66	4.327868852	1.66E-07	3.11E-04
GOTERM_BP_ALL	GO:0051130~positive regulation of cellular component organization	41	2.68852459	1.71E-07	3.20E-04
GOTERM_BP_ALL	GO:0002376~immune system process	105	6.885245902	3.31E-07	6.19E-04
GOTERM_BP_ALL	GO:0051270~regulation of cell motion	40	2.62295082	3.63E-07	6.80E-04
GOTERM_BP_ALL	GO:0010035~response to inorganic substance	51	3.344262295	4.05E-07	7.57E-04
GOTERM_BP_ALL	GO:0051049~regulation of transport	75	4.918032787	4.08E-07	7.63E-04
GOTERM_BP_ALL	GO:0032535~regulation of cellular component size	46	3.016393443	4.37E-07	8.18E-04
GOTERM_BP_ALL	GO:0040012~regulation of locomotion	40	2.62295082	4.86E-07	9.09E-04
GOTERM_BP_ALL	GO:0048145~regulation of fibroblast proliferation	16	1.049180328	5.54E-07	0.001036142
GOTERM_BP_ALL	GO:0042325~regulation of phosphorylation	67	4.393442623	6.35E-07	0.00118739
GOTERM_BP_ALL	GO:0043627~response to estrogen stimulus	34	2.229508197	6.64E-07	0.001242658
GOTERM_BP_ALL	GO:0001666~response to hypoxia	40	2.62295082	7.43E-07	0.001389271
GOTERM_BP_ALL	GO:0007584~response to nutrient	42	2.754098361	7.89E-07	0.0014759
GOTERM_BP_ALL	GO:0042552~myelination	17	1.114754098	8.12E-07	0.001519944
GOTERM_BP_ALL	GO:0031667~response to nutrient levels	52	3.409836066	8.31E-07	0.001555266
GOTERM_BP_ALL	GO:0030334~regulation of cell migration	36	2.360655738	1.08E-06	0.002013089
GOTERM_BP_ALL	GO:0022402~cell cycle process	61	4	1.10E-06	0.002056131
GOTERM_BP_ALL	GO:0051174~regulation of phosphorus metabolic process	68	4.459016393	1.28E-06	0.002386636
GOTERM_BP_ALL	GO:0019220~regulation of phosphate metabolic process	68	4.459016393	1.28E-06	0.002386636
GOTERM_BP_ALL	GO:0022008~neurogenesis	95	6.229508197	1.34E-06	0.002510506

GOTERM_BP_ALL	GO:0000279~M phase	37	2.426229508	1.42E-06	0.002653064
GOTERM_BP_ALL	GO:0014070~response to organic cyclic substance	42	2.754098361	1.51E-06	0.002829304
GOTERM_BP_ALL	GO:0007167~enzyme linked receptor protein signaling pathway	51	3.344262295	1.57E-06	0.002927937
GOTERM_BP_ALL	GO:0016044~membrane organization	52	3.409836066	1.97E-06	0.00368024
GOTERM_BP_ALL	GO:0010646~regulation of cell communication	128	8.393442623	2.02E-06	0.003783992
GOTERM_BP_ALL	GO:0010769~regulation of cell morphogenesis involved in differentiation	25	1.639344262	2.17E-06	0.004051604
GOTERM_BP_ALL	GO:0060548~negative regulation of cell death	58	3.803278689	2.19E-06	0.004097242
GOTERM_BP_ALL	GO:0043434~response to peptide hormone stimulus	43	2.819672131	2.34E-06	0.004370514
GOTERM_BP_ALL	GO:0043066~negative regulation of apoptosis	57	3.737704918	2.67E-06	0.005001089
GOTERM_BP_ALL	GO:0051726~regulation of cell cycle	46	3.016393443	2.98E-06	0.005572118
GOTERM_BP_ALL	GO:0007010~cytoskeleton organization	55	3.606557377	2.98E-06	0.005579096
GOTERM_BP_ALL	GO:0035295~tube development	46	3.016393443	3.33E-06	0.006232113
GOTERM_BP_ALL	GO:0060284~regulation of cell development	44	2.885245902	3.91E-06	0.007316977
GOTERM_BP_ALL	GO:0043069~negative regulation of programmed cell death	57	3.737704918	4.29E-06	0.00802262
GOTERM_BP_ALL	GO:0042246~tissue regeneration	16	1.049180328	4.54E-06	0.008495808
GOTERM_BP_ALL	GO:0016477~cell migration	47	3.081967213	4.73E-06	0.008849035
GOTERM_BP_ALL	GO:0032101~regulation of response to external stimulus	34	2.229508197	4.77E-06	0.008923318
GOTERM_BP_ALL	GO:0006897~endocytosis	33	2.163934426	4.89E-06	0.009148131
GOTERM_BP_ALL	GO:0010324~membrane invagination	33	2.163934426	4.89E-06	0.009148131
GOTERM_BP_ALL	GO:0051129~negative regulation of cellular component organization	29	1.901639344	4.92E-06	0.009200311
GOTERM_BP_ALL	GO:0001558~regulation of cell growth	35	2.295081967	5.30E-06	0.009907052
GOTERM_BP_ALL	GO:0048878~chemical homeostasis	75	4.918032787	5.39E-06	0.010077289
GOTERM_BP_ALL	GO:0009628~response to abiotic stimulus	64	4.196721311	5.60E-06	0.010468877
GOTERM_BP_ALL	GO:0010721~negative regulation of cell development	18	1.180327869	5.95E-06	0.011133872
GOTERM_BP_ALL	GO:0040007~growth	41	2.68852459	6.19E-06	0.01157346
GOTERM_BP_ALL	GO:0008361~regulation of cell size	37	2.426229508	6.27E-06	0.011738017
GOTERM_BP_ALL	GO:0048583~regulation of response to stimulus	69	4.524590164	6.44E-06	0.012048677
GOTERM_BP_ALL	GO:0048646~anatomical structure formation involved in morphogenesis	55	3.606557377	7.71E-06	0.014427074
GOTERM_BP_ALL	GO:0006954~inflammatory response	41	2.68852459	7.80E-06	0.014599497
GOTERM_BP_ALL	GO:0000278~mitotic cell cycle	42	2.754098361	8.08E-06	0.015120156
GOTERM_BP_ALL	GO:0032355~response to estradiol stimulus	22	1.442622951	9.01E-06	0.016854846
GOTERM_BP_ALL	GO:0001525~angiogenesis	28	1.836065574	9.20E-06	0.0172059
GOTERM_BP_ALL	GO:0043067~regulation of programmed cell death	96	6.295081967	9.24E-06	0.017279515
GOTERM_BP_ALL	GO:0048699~generation of neurons	86	5.639344262	9.59E-06	0.017936757
GOTERM_BP_ALL	GO:0042592~homeostatic process	99	6.491803279	9.70E-06	0.018153725
GOTERM_BP_ALL	GO:0040008~regulation of growth	51	3.344262295	9.72E-06	0.018178015
GOTERM_BP_ALL	GO:0010941~regulation of cell death	96	6.295081967	1.10E-05	0.020558655
GOTERM_BP_ALL	GO:0006928~cell motion	65	4.262295082	1.12E-05	0.020893452

GOTERM_BP_ALL	GO:0009966~regulation of signal transduction	103	6.754098361	1.15E-05	0.021604847
GOTERM_BP_ALL	GO:0042391~regulation of membrane potential	32	2.098360656	1.17E-05	0.021930673
GOTERM_BP_ALL	GO:0000087~M phase of mitotic cell cycle	25	1.639344262	1.39E-05	0.026013064
GOTERM_BP_ALL	GO:0051301~cell division	31	2.032786885	1.39E-05	0.02604052
GOTERM_BP_ALL	GO:0032787~monocarboxylic acid metabolic process	51	3.344262295	1.53E-05	0.028688871
GOTERM_BP_ALL	GO:0050768~negative regulation of neurogenesis	17	1.114754098	1.58E-05	0.029606777
GOTERM_BP_ALL	GO:0042981~regulation of apoptosis	94	6.163934426	1.59E-05	0.029674609
GOTERM_BP_ALL	GO:0019228~regulation of action potential in neuron	19	1.245901639	1.60E-05	0.029986047
GOTERM_BP_ALL	GO:0001932~regulation of protein amino acid phosphorylation	34	2.229508197	1.81E-05	0.033784158
GOTERM_BP_ALL	GO:0050770~regulation of axonogenesis	19	1.245901639	2.00E-05	0.03741161
GOTERM_BP_ALL	GO:0051179~localization	279	18.29508197	2.00E-05	0.037466393
GOTERM_BP_ALL	GO:0048146~positive regulation of fibroblast proliferation	12	0.786885246	2.14E-05	0.040088354
GOTERM_BP_ALL	GO:0032268~regulation of cellular protein metabolic process	64	4.196721311	2.16E-05	0.040326017
GOTERM_BP_ALL	GO:0043523~regulation of neuron apoptosis	25	1.639344262	2.29E-05	0.042916873
GOTERM_BP_ALL	GO:0032386~regulation of intracellular transport	17	1.114754098	2.60E-05	0.048540623
GOTERM_BP_ALL	GO:0040017~positive regulation of locomotion	24	1.573770492	2.63E-05	0.049280822
GOTERM_BP_ALL	GO:0032989~cellular component morphogenesis	59	3.868852459	2.65E-05	0.049617301

Table S2. The molecular functions (MF) in which DEGs are enriched.

Category	Term	Count	%	PValue	FDR
GOTERM_MF_ALL	GO:0005515~protein binding	784	51.40983607	8.92E-32	1.45E-28
GOTERM_MF_ALL	GO:0005488~binding	1039	68.13114754	3.49E-23	5.68E-20
GOTERM_MF_ALL	GO:0001871~pattern binding	36	2.360655738	1.00E-11	1.64E-08
GOTERM_MF_ALL	GO:0030247~polysaccharide binding	36	2.360655738	1.00E-11	1.64E-08
GOTERM_MF_ALL	GO:0005539~glycosaminoglycan binding	32	2.098360656	1.24E-10	2.03E-07
GOTERM_MF_ALL	GO:0008092~cytoskeletal protein binding	73	4.786885246	1.37E-10	2.23E-07
GOTERM_MF_ALL	GO:0008201~heparin binding	26	1.704918033	3.28E-10	5.35E-07
GOTERM_MF_ALL	GO:0019838~growth factor binding	29	1.901639344	4.00E-10	6.52E-07
GOTERM_MF_ALL	GO:0046983~protein dimerization activity	84	5.508196721	1.50E-08	2.44E-05
GOTERM_MF_ALL	GO:0003824~catalytic activity	473	31.01639344	2.11E-08	3.43E-05
GOTERM_MF_ALL	GO:0042802~identical protein binding	86	5.639344262	4.45E-07	7.25E-04
GOTERM_MF_ALL	GO:0042803~protein homodimerization activity	54	3.540983607	1.04E-06	0.001690603
GOTERM_MF_ALL	GO:0030246~carbohydrate binding	56	3.672131148	1.28E-06	0.002091313
GOTERM_MF_ALL	GO:0003779~actin binding	42	2.754098361	4.35E-06	0.007086154
GOTERM_MF_ALL	GO:0016491~oxidoreductase activity	95	6.229508197	4.54E-06	0.007395056
GOTERM_MF_ALL	GO:0005102~receptor binding	98	6.426229508	1.44E-05	0.023526523

Table S3. The cell components (CC) in which DEGs are enriched.

Category	Term	Count	%	PValue	FDR
GOTERM_CC_ALL	GO:0044421~extracellular region part	138	9.049180328	4.43E-20	6.46E-17
GOTERM_CC_ALL	GO:0005737~cytoplasm	676	44.32786885	2.21E-19	3.22E-16
GOTERM_CC_ALL	GO:0005576~extracellular region	191	12.52459016	2.38E-13	3.46E-10
GOTERM_CC_ALL	GO:0044424~intracellular part	833	54.62295082	5.74E-13	8.37E-10
GOTERM_CC_ALL	GO:0005783~endoplasmic reticulum	130	8.524590164	8.35E-13	1.22E-09
GOTERM_CC_ALL	GO:0031012~extracellular matrix	61	4	8.75E-13	1.27E-09
GOTERM_CC_ALL	GO:0005615~extracellular space	94	6.163934426	1.47E-12	2.14E-09
GOTERM_CC_ALL	GO:0000267~cell fraction	148	9.704918033	2.97E-11	4.33E-08
GOTERM_CC_ALL	GO:0005578~proteinaceous extracellular matrix	53	3.475409836	3.97E-11	5.78E-08
GOTERM_CC_ALL	GO:0005886~plasma membrane	300	19.67213115	5.12E-11	7.47E-08
GOTERM_CC_ALL	GO:0044444~cytoplasmic part	495	32.45901639	1.15E-10	1.68E-07
GOTERM_CC_ALL	GO:0005622~intracellular	863	56.59016393	7.07E-10	1.03E-06
GOTERM_CC_ALL	GO:0044459~plasma membrane part	181	11.86885246	1.33E-08	1.94E-05
GOTERM_CC_ALL	GO:0043226~organelle	702	46.03278689	1.88E-08	2.74E-05
GOTERM_CC_ALL	GO:0005829~cytosol	154	10.09836066	2.33E-08	3.39E-05
GOTERM_CC_ALL	GO:0043229~intracellular organelle	697	45.70491803	5.25E-08	7.65E-05
GOTERM_CC_ALL	GO:0005626~insoluble fraction	112	7.344262295	6.41E-08	9.35E-05
GOTERM_CC_ALL	GO:0005856~cytoskeleton	131	8.590163934	1.66E-07	2.42E-04
GOTERM_CC_ALL	GO:0043227~membrane-bounded organelle	616	40.39344262	2.01E-07	2.93E-04
GOTERM_CC_ALL	GO:0043231~intracellular membrane-bounded organelle	615	40.32786885	2.06E-07	3.01E-04
GOTERM_CC_ALL	GO:0042995~cell projection	106	6.950819672	3.63E-07	5.29E-04
GOTERM_CC_ALL	GO:0005624~membrane fraction	104	6.819672131	5.71E-07	8.32E-04
GOTERM_CC_ALL	GO:0044432~endoplasmic reticulum part	49	3.213114754	1.09E-06	0.001590666
GOTERM_CC_ALL	GO:0044422~organelle part	352	23.08196721	1.55E-06	0.002258071
GOTERM_CC_ALL	GO:0044446~intracellular organelle part	349	22.8852459	1.71E-06	0.002486667
GOTERM_CC_ALL	GO:0005625~soluble fraction	53	3.475409836	1.82E-06	0.002650547
GOTERM_CC_ALL	GO:0012505~endomembrane system	93	6.098360656	2.30E-06	0.003358309
GOTERM_CC_ALL	GO:0005794~Golgi apparatus	95	6.229508197	4.46E-06	0.00650687
GOTERM_CC_ALL	GO:0000777~condensed chromosome kinetochore	13	0.852459016	6.82E-06	0.009935462
GOTERM_CC_ALL	GO:0005789~endoplasmic reticulum membrane	40	2.62295082	6.96E-06	0.010148462
GOTERM_CC_ALL	GO:0042175~nuclear envelope-endoplasmic reticulum network	41	2.68852459	1.28E-05	0.018586966
GOTERM_CC_ALL	GO:0042598~vesicular fraction	47	3.081967213	1.35E-05	0.019682575
GOTERM_CC_ALL	GO:0005792~microsome	46	3.016393443	1.35E-05	0.019708131
GOTERM_CC_ALL	GO:0000779~condensed chromosome, centromeric region	14	0.918032787	1.38E-05	0.020068608
GOTERM_CC_ALL	GO:0044430~cytoskeletal part	96	6.295081967	1.41E-05	0.02057559

Table S4. The KEGG pathways for which DEGs are enriched.

Category	Term	Count	%	PValue	FDR
KEGG_PATHWAY	rno00100:Steroid biosynthesis	12	0.786885246	8.81E-08	1.08E-04
KEGG_PATHWAY	rno00900:Terpenoid backbone biosynthesis	9	0.590163934	2.07E-05	0.025513219
KEGG_PATHWAY	rno04110:Cell cycle	30	1.967213115	2.13E-05	0.026275255

2. NSN vs. ISN

Table S5. The biological processes (BP) for which DEGs are enriched.

Category	Term	Count	%	PValue	FDR
GOTERM_BP_ALL	GO:0007275~multicellular organismal development	34	37.77777778	1.82E-08	2.93E-05
GOTERM_BP_ALL	GO:0048856~anatomical structure development	32	35.55555556	5.43E-08	8.73E-05
GOTERM_BP_ALL	GO:0032502~developmental process	35	38.88888889	6.11E-08	9.83E-05
GOTERM_BP_ALL	GO:0048513~organ development	27	30	1.06E-07	1.71E-04
GOTERM_BP_ALL	GO:0009653~anatomical structure morphogenesis	22	24.44444444	1.32E-07	2.12E-04
GOTERM_BP_ALL	GO:0048731~system development	30	33.33333333	2.46E-07	3.96E-04
GOTERM_BP_ALL	GO:0009888~tissue development	16	17.77777778	8.20E-07	0.001318273
GOTERM_BP_ALL	GO:0030239~myofibril assembly	5	5.555555556	3.45E-06	0.005543558
GOTERM_BP_ALL	GO:0030154~cell differentiation	23	25.55555556	4.75E-06	0.007638083
GOTERM_BP_ALL	GO:0007155~cell adhesion	13	14.44444444	5.58E-06	0.008967159
GOTERM_BP_ALL	GO:0022610~biological adhesion	13	14.44444444	5.58E-06	0.008967159
GOTERM_BP_ALL	GO:0048869~cellular developmental process	23	25.55555556	8.83E-06	0.01420193
GOTERM_BP_ALL	GO:0031032~actomyosin structure organization	5	5.555555556	1.21E-05	0.019518768
GOTERM_BP_ALL	GO:0010927~cellular component assembly involved in morphogenesis	5	5.555555556	2.76E-05	0.044415277

Table S6. The molecular functions (MF) in which DEGs are enriched.

Category	Term	Count	%	PValue	FDR
GOTERM_MF_ALL	GO:0005515~protein binding	706	50.97472924	5.65E-27	9.12E-24
GOTERM_MF_ALL	GO:0005488~binding	954	68.88086643	1.16E-24	1.87E-21
GOTERM_MF_ALL	GO:0030247~polysaccharide binding	36	2.599277978	6.34E-13	1.02E-09
GOTERM_MF_ALL	GO:0001871~pattern binding	36	2.599277978	6.34E-13	1.02E-09
GOTERM_MF_ALL	GO:0019838~growth factor binding	31	2.238267148	1.15E-12	1.86E-09
GOTERM_MF_ALL	GO:0005539~glycosaminoglycan binding	32	2.310469314	1.07E-11	1.73E-08
GOTERM_MF_ALL	GO:0008201~heparin binding	26	1.877256318	4.20E-11	6.78E-08
GOTERM_MF_ALL	GO:0008092~cytoskeletal protein binding	66	4.76534296	1.66E-09	2.68E-06
GOTERM_MF_ALL	GO:0005509~calcium ion binding	94	6.78700361	1.05E-08	1.70E-05
GOTERM_MF_ALL	GO:0030246~carbohydrate binding	57	4.115523466	2.25E-08	3.64E-05
GOTERM_MF_ALL	GO:0019899~enzyme binding	70	5.054151625	5.45E-07	8.80E-04
GOTERM_MF_ALL	GO:0005102~receptor binding	94	6.78700361	2.47E-06	0.003993379
GOTERM_MF_ALL	GO:0008289~lipid binding	54	3.898916968	3.75E-06	0.006062398

GOTERM_MF_ALL	GO:0003824~catalytic activity	415	29.96389892	1.16E-05	0.018749283
GOTERM_MF_ALL	GO:0046983~protein dimerization activity	69	4.981949458	1.32E-05	0.021387805

Table S7. The cell components (CC) in which DEGs are enriched.

Category	Term	Count	%	PValue	FDR
GOTERM_CC_ALL	GO:0044421~extracellular region part	131	9.458483755	1.36E-20	1.94E-17
GOTERM_CC_ALL	GO:0005886~plasma membrane	298	21.51624549	4.67E-16	6.33E-13
GOTERM_CC_ALL	GO:0005576~extracellular region	186	13.42960289	6.96E-16	9.55E-13
GOTERM_CC_ALL	GO:0031012~extracellular matrix	60	4.332129964	5.13E-14	7.35E-11
GOTERM_CC_ALL	GO:0005578~proteinaceous extracellular matrix	53	3.826714801	1.12E-12	1.61E-09
GOTERM_CC_ALL	GO:0005737~cytoplasm	591	42.67148014	4.64E-12	6.64E-09
GOTERM_CC_ALL	GO:0005615~extracellular space	87	6.281588448	5.27E-12	7.55E-09
GOTERM_CC_ALL	GO:0000267~cell fraction	138	9.963898917	3.07E-11	4.40E-08
GOTERM_CC_ALL	GO:0044459~plasma membrane part	174	12.5631769	4.11E-10	5.88E-07
GOTERM_CC_ALL	GO:0009986~cell surface	57	4.115523466	2.19E-07	3.14E-04
GOTERM_CC_ALL	GO:0005626~insoluble fraction	102	7.364620939	2.80E-07	4.01E-04
GOTERM_CC_ALL	GO:0044444~cytoplasmic part	435	31.40794224	3.01E-07	4.31E-04
GOTERM_CC_ALL	GO:0005783~endoplasmic reticulum	102	7.364620939	8.57E-07	0.001227174
GOTERM_CC_ALL	GO:0005829~cytosol	136	9.819494585	1.08E-06	0.00154721
GOTERM_CC_ALL	GO:0005625~soluble fraction	50	3.610108303	1.44E-06	0.002058325
GOTERM_CC_ALL	GO:0005624~membrane fraction	95	6.859205776	1.73E-06	0.002478703
GOTERM_CC_ALL	GO:0005794~Golgi apparatus	88	6.353790614	5.45E-06	0.00780987
GOTERM_CC_ALL	GO:0042995~cell projection	94	6.78700361	5.85E-06	0.008384075
GOTERM_CC_ALL	GO:0005856~cytoskeleton	113	8.158844765	1.77E-05	0.025351146
GOTERM_CC_ALL	GO:0031982~vesicle	82	5.920577617	2.16E-05	0.030874773
GOTERM_CC_ALL	GO:0016324~apical plasma membrane	27	1.949458484	2.42E-05	0.034616503

Table S8. The KEGG pathways in which DEGs are enriched.

Category	Term	Count	%	PValue	FDR
KEGG_PATHWAY	rno00100:Steroid biosynthesis	13	0.938628159	2.03E-09	2.48E-06
KEGG_PATHWAY	rno00900:Terpenoid backbone biosynthesis	9	0.649819495	1.18E-05	0.014336122

Section 3

Lists of differentially expressed genes (DEGs) between four different groups

Table S9. DEGs between normal motor nerves and injured motor nerves

RNA_nucleotide_accession	GeneID	GeneSymbol	log2Ratio	Probability
NM_032070	84017	Hmga2	11.43802	0.973664
NM_031089	81753	Pth2r	10.34076	0.940366
NM_017096	25419	Crp	9.523617	0.891174
NM_080411	140595	Gpr83	8.338272	0.989241
NM_001106545	296395	Kcng1	7.857937	0.981681
NM_145083	246296	Lrrc15	7.625354	0.981751
NM_019139	25453	Gdnf	7.43232	0.962236
NM_001106579	296789	Sema3e	6.914721	0.976195
NM_001106858	301012	Col7a1	6.819478	0.969866
NM_031689	64348	Cryba4	6.790835	0.941913
NM_001044269	500750	Tmem196	6.783025	0.929149
NM_199082	287884	Sectm1b	6.675933	0.967124
NM_031091	81755	Rab3b	6.675192	0.990752
NM_022211	60662	Fgf5	6.464996	0.991245
NM_133385	170896	Ucn2	6.334629	0.983404
NM_001108111	315294	Wnt10b	6.296295	0.936463
NM_017091	25204	Pcsk1	6.267202	0.988889
NM_001106629	297738	Steap1	6.248	0.925703
NM_001105734	63995	Dusp10	6.207022	0.99244
NM_001109419	680551	Apoc4	6.204129	0.936111
NM_001107280	306081	Pcdh20	6.203003	0.972785
NM_031538	24930	Cd8a	6.154764	0.901793
NM_001100964	296935	Lmod2	6.063833	0.890893
NM_012610	24596	Ngfr	6.048107	0.995394
NM_053763	114700	Cyp27b1	5.996982	0.939733
NM_021865	60462	Batf3	5.601557	0.925809
NM_001113781	360559	Fam64a	5.578757	0.958685
NM_053896	116676	Aldh1a2	5.569265	0.978411
NM_001079937	290326	Pbk	5.545188	0.932736
NM_130407	154516	Ugt1a7c	5.530447	0.977391
NM_012690	24891	Abcb4	5.47246	0.970359
NM_001106134	291441	Ska1	5.440152	0.838924
NM_001107504	308511	Chst8	5.406534	0.857489
NM_012932	25415	Crmp1	5.357279	0.959599
NM_001007648	297594	Cdca3	5.323497	0.955591
NM_022634	64569	Lst1	5.223891	0.952602
NM_001109536	689388	Ptx3	5.194447	0.923734
NM_001009470	363088	Ccnb2	5.019278	0.951512
NM_001106671	298300	Ttc22	5.015773	0.904536
NM_001009654	295661	Spc25	4.95277	0.920851
NM_001108344	360847	Ube2t	4.907917	0.832032

NM_053881	116660	Ptprn	4.835543	0.973031
NM_019296	54237	Cdk1	4.806697	0.963502
NM_001107160	304648	Asf1b	4.773441	0.897398
NM_001108662	362510	Melk	4.771929	0.826582
NM_001025740	362720	Rrm2	4.729684	0.970781
NM_001130500	300126	Gtse1	4.634873	0.916385
NM_001024257	307805	Cenpt	4.607645	0.872152
NM_022256	64022	Btc	4.599311	0.968636
NM_001025646	294074	Cep55	4.594966	0.881505
NM_012752	25145	Cd24	4.563498	0.97275
NM_001271243	300219	Troap	4.556414	0.898945
NM_145773	252915	Mxd3	4.538102	0.840928
NM_001107702	310694	Fcrl2	4.509584	0.960302
NM_053719	114511	Emb	4.504141	0.940401
NM_052800	24834	Tk1	4.503936	0.954782
NM_001008882	316129	Uhrf1	4.503229	0.922644
NM_001271181	100361733	Ociad2	4.452375	0.937236
NM_001246319	689399	Cenpw	4.446923	0.828094
NM_001009353	301265	Pla2g7	4.44595	0.899367
NM_001108774	363112	Mrap2	4.398673	0.822539
NM_013077	25609	Tub	4.328653	0.846203
NM_001108172	315852	Ttk	4.308588	0.844691
NM_001013894	292594	Lilrb4	4.290132	0.88256
NM_001108155	315740	Kif23	4.244908	0.902743
NM_001106991	303004	Tmem8a	4.243774	0.957841
NM_201418	300795	Ns5atp9	4.233718	0.931927
NM_001107846	312052	Steap2	4.210451	0.932138
NM_001025768	499933	Dsn1	4.19969	0.896238
NM_139089	245920	Cxcl10	4.183599	0.967616
NM_022274	64041	Birc5	4.172122	0.914627
NM_001108287	360591	Prr11	4.168988	0.873699
NM_001191709	310621	Iqgap3	4.167926	0.924121
NM_001169139	306575	Ckap2	4.144894	0.921519
NM_022196	60584	Lif	4.140119	0.936603
NM_033237	29141	Gal	4.119626	0.94237
NM_001100827	257649	Cenpf	4.104196	0.840999
NM_019344	54297	Rgs8	4.087673	0.864346
NM_001107529	308761	Prc1	4.069281	0.943847
NM_172333	282836	Cthrc1	4.05474	0.820253
NM_001108223	316507	Shd	4.001592	0.909459
NM_053744	114587	Dlk1	4.001298	0.935478
NM_139097	245956	Scn3b	3.982165	0.94898
NM_022300	64160	Basp1	3.972078	0.959318
NM_139327	117062	Hmga1	3.944464	0.964979

NM_001004424	360962	Tacc3	3.940319	0.919937
NM_001126270	301701	Ndc80	3.937655	0.906118
NM_001107762	311336	Nusap1	3.92859	0.918565
NM_001108633	362384	Reep1	3.927168	0.883931
NM_001109181	499593	Sox2	3.910047	0.962271
NM_001106711	298850	Cenpa	3.905427	0.821976
NM_012912	25389	Atf3	3.856023	0.964592
NM_001039344	500040	Tes	3.850816	0.958544
NM_001009645	293502	Kif22	3.842676	0.931188
NM_138541	171577	Epcam	3.835043	0.881857
NM_001271366	291234	Mki67	3.827715	0.933052
NM_001077589	360857	Rgs16	3.817385	0.960162
NM_001014095	315952	Dzip1l	3.788893	0.941491
NM_012488	24153	A2m	3.779626	0.962588
NM_001004264	311325	Knstrn	3.776068	0.895253
NM_031517	24553	Met	3.746965	0.928622
NM_012732	25055	Lipa	3.744976	0.957208
NM_001109418	680531	Sapcd2	3.744192	0.860197
NM_144762	140720	Baalc	3.74098	0.948101
NM_001031638	287435	Cd68	3.719053	0.956786
NM_024127	25112	Gadd45a	3.708633	0.96097
NM_001169112	171304	Kif11	3.701821	0.862412
NM_001108158	315750	Lctl	3.693624	0.811674
NM_207615	365395	Clcf1	3.680476	0.940014
NM_001106542	296368	Ube2c	3.652411	0.943319
NM_001100474	117524	Ccnf	3.649559	0.847609
NM_001005878	294286	Kifc1	3.629403	0.88365
NM_001105925	288626	Mmp17	3.613687	0.938889
NM_022391	64193	Pttg1	3.613686	0.917651
NM_001109204	499870	Rad51	3.607527	0.832208
NM_001013886	291081	Tubb2b	3.607381	0.956329
NM_001012028	304951	Nuf2	3.600337	0.901723
NM_001168524	296060	Arhgap11a	3.597242	0.892722
NM_022624	64559	Slc22a23	3.570301	0.953903
NM_001108495	361615	Relt	3.549031	0.821554
NM_019333	54283	Pfkfb4	3.548974	0.936603
NM_001134969	500430	Ankrd6	3.51415	0.908228
NM_144750	246266	Aspg	3.51042	0.80116
NM_022194	60582	Il1rn	3.503569	0.862342
NM_181086	302965	Tnfrsf12a	3.479162	0.951864
NM_001191813	316602	Hjurp	3.468173	0.854044
NM_001106192	292071	Cdt1	3.462877	0.864487
NM_001109102	498533	Fam167a	3.462146	0.824684
NM_001039019	303575	Kif18b	3.451402	0.854079

NM_133298	113955	Gpnmb	3.4342	0.953446
NM_001106536	296344	Mybl2	3.428256	0.815577
NM_001106170	291885	Mcm5	3.410944	0.883439
NM_053734	114553	Ncf1	3.402417	0.940963
NM_001106884	301227	Trem2	3.400592	0.936006
NM_133393	170905	Lfng	3.39114	0.944726
NM_001013433	364396	Arl11	3.385249	0.908826
NM_001109163	499322	Fam111a	3.381531	0.827215
NM_001077650	366270	Edn3	3.380958	0.918425
NM_001106507	296137	Bub1	3.378279	0.842616
NM_030828	58920	Gpc1	3.368199	0.952461
NM_053397	362572	Artn	3.361049	0.913502
NM_001107946	313370	Hook1	3.358194	0.81294
NM_001107315	306506	Pragmin	3.341371	0.92071
NM_017100	25515	Plk1	3.287569	0.871449
NM_012953	25445	Fosl1	3.27437	0.919128
NM_001191763	298506	Mycl	3.270725	0.89564
NM_001107790	311546	Tpx2	3.268118	0.922293
NM_001011930	292206	Trip13	3.266797	0.800141
NM_053702	114494	Ccna2	3.252152	0.916807
NM_001100684	305450	Sh3bp2	3.245039	0.91519
NM_001025050	500545	Cdca8	3.234115	0.879044
NM_016994	24232	C3	3.227538	0.838361
NM_001107159	304608	Mmp19	3.227163	0.942792
NM_013185	25734	Hck	3.214358	0.89557
NM_133416	170929	Bcl2a1	3.210764	0.900105
NM_012959	25454	Gfra1	3.206443	0.948418
NM_013026	25216	Sdc1	3.187404	0.887693
NM_001108862	364190	Rasl10a	3.186876	0.936076
NM_001173386	502902	Clec7a	3.176398	0.912447
NM_017287	29685	Mcm6	3.162338	0.903973
NM_001107669	310344	Plk4	3.158401	0.801195
NM_001015032	366061	Galnt3	3.156759	0.875949
NM_012964	25460	Hmmr	3.147939	0.834845
NM_012955	25448	Cenpi	3.13899	0.804044
NM_001130583	689069	Parvg	3.127154	0.863502
NM_001191950	499254	Tmc7	3.119277	0.914276
NM_001192006	501872	Cd84	3.079097	0.84898
NM_001191991	500915	Fam19a5	3.061013	0.939346
NM_057120	117260	Nudt1	3.05383	0.818601
NM_001107807	311676	Pmepa1	3.050393	0.943038
NM_001271362	294297	Scube3	3.043325	0.809775
NM_001007554	362650	Fblim1	3.022791	0.905696
NM_031832	83781	Lgals3	3.016774	0.943952

NM_001107777	311426	Siglec1	3.013736	0.886146
NM_001014268	367113	Lrrc1	3.013355	0.888783
NM_057212	117582	Tmem158	3.006231	0.940858
NM_182824	315716	Cd276	3.003374	0.940999
NM_171992	58919	Ccnd1	2.980842	0.942651
NM_001135802	289997	Dlgap5	2.974053	0.868073
NM_031126	81804	Stxbp2	2.972851	0.864733
NM_017244	29563	Crabp2	2.952025	0.940858
NM_001191805	316273	Mcm3	2.930832	0.892827
NM_022183	360243	Top2a	2.929034	0.903657
NM_001271205	364712	Sox4	2.916941	0.938432
NM_172067	64456	Spon1	2.912981	0.901793
NM_001108112	315298	Racgap1	2.901125	0.879501
NM_001012125	315714	Loxl1	2.891913	0.934951
NM_013080	25613	Ptprz1	2.88011	0.82711
NM_031821	83722	Plk2	2.860201	0.93474
NM_001173344	308478	Spred3	2.854047	0.827004
NM_001107297	306279	Arhgap22	2.850665	0.912834
NM_001109207	499914	Gins1	2.848458	0.826442
NM_012867	25338	Ninj1	2.84634	0.937377
NM_001025767	499356	Blnk	2.841786	0.88052
NM_001012127	315994	Mapkapk3	2.83873	0.895394
NM_001107512	308582	Tead2	2.835073	0.890893
NM_001037780	309684	Itgb2	2.821744	0.892932
NM_001126291	366205	Flrt3	2.802155	0.928129
NM_022617	64552	Mpeg1	2.802004	0.92602
NM_012687	24886	Tbxas1	2.79639	0.852989
NM_012907	25383	Apobec1	2.796069	0.905063
NM_001100802	502317	Aplp1	2.787951	0.910338
NM_031633	58921	Foxm1	2.769026	0.868179
NM_001024277	366959	Gcat	2.760897	0.918495
NM_001191636	309499	Myof	2.753032	0.930837
NM_001135016	308602	Tmem86a	2.750744	0.901617
NM_001205348	501736	Cd300a	2.750574	0.849578
NM_138529	171563	Nav2	2.746038	0.919796
NM_001170487	293736	Myrf	2.735725	0.91853
NM_178095	313210	Abca1	2.732089	0.924824
NM_001191622	308431	Phldb3	2.729336	0.891842
NM_001271151	688611	Cetn4	2.726781	0.875703
NM_001105766	287101	Pkmyt1	2.723473	0.858474
NM_001013927	297694	Plbd1	2.716784	0.852883
NM_053698	114490	Cited2	2.709625	0.931259
NM_001106314	293618	Ifitm1	2.698618	0.935689
NM_130822	170641	Lphn3	2.672702	0.905977

NM_001014790	310486	Rarres1	2.666726	0.889627
NM_019262	29687	C1qb	2.66564	0.931681
NM_001007679	305070	Tmem206	2.649215	0.91744
NM_001108472	361514	Meis3	2.62133	0.89244
NM_001105769	287110	Tbc1d24	2.609136	0.885091
NM_001191862	362332	Flnc	2.607653	0.92166
NM_001168543	296371	Pltp	2.576161	0.931013
NM_053372	84386	Slpi	2.571313	0.871554
NM_001005384	310132	Osmr	2.564416	0.881329
NM_001014205	363013	Tmem123	2.563113	0.928516
NM_001191680	305449	Mfsd10	2.560688	0.910478
NM_021584	83825	Dclk1	2.558446	0.928586
NM_001009422	313211	Nipsnap3b	2.558138	0.896976
NM_001024262	312727	Tspan11	2.543527	0.91948
NM_001127640	684050	PCOLCE2	2.542888	0.886111
NM_001008515	298566	C1qa	2.542864	0.93038
NM_001108965	366262	Rbm38	2.539294	0.908509
NM_001013158	310508	B3galnt1	2.533674	0.906927
NM_053905	116685	Lmnb1	2.519873	0.882876
NM_172322	282817	Pycard	2.506725	0.893987
NM_207603	304966	Fcgr3a	2.50545	0.807138
NM_001039028	316333	Actr1b	2.501676	0.928692
NM_001191623	308492	Wdr62	2.493881	0.833228
NM_019165	29197	Il18	2.491822	0.877567
NM_012980	25481	Mmp11	2.484078	0.891842
NM_001047853	293019	Arrdc4	2.478265	0.869269
NM_031325	83472	Ugdh	2.477298	0.922574
NM_019354	54315	Ucp2	2.474269	0.930485
NM_053861	116640	Tnc	2.471335	0.925105
NM_019289	54227	Arpc1b	2.463245	0.931259
NM_213561	406195	Tcf19	2.457185	0.910197
NM_001007729	360918	Pf4	2.454527	0.899367
NM_001107796	311598	Fam83d	2.452281	0.819198
NM_001009646	293504	Qprt	2.449896	0.920886
NM_001107873	312538	Mcm2	2.44878	0.889311
NM_012934	25418	Dpysl3	2.44525	0.911498
NM_019358	54320	Pdpn	2.442891	0.92507
NM_001106996	303073	Cyfip2	2.437589	0.917968
NM_022618	64553	Akap6	2.436599	0.807384
NM_012523	24251	Cd53	2.429733	0.907841
NM_001106112	291137	Gmnn	2.42599	0.83052
NM_030859	81517	Mdk	2.40672	0.926406
NM_017320	50654	Ctss	2.403864	0.923347
NM_001106577	296762	Phtf2	2.40088	0.904536

NM_001107111	304091	Il10rb	2.390353	0.91948
NM_001106283	293154	Folr2	2.387723	0.899719
NM_001135992	498276	LOC498276	2.38536	0.900844
NM_022396	64199	Gng11	2.384	0.921484
NM_001190999	679812	Plekhg1	2.382009	0.908228
NM_023959	66015	Adamts4	2.374017	0.918917
NM_013123	25663	Il1r1	2.37347	0.878024
NM_001106028	289993	Cdkn3	2.363665	0.800457
NM_001126083	498709	Cks2	2.361525	0.885935
NM_001109636	691306	Tmem202	2.35826	0.847222
NM_171993	64515	Cdc20	2.356215	0.880415
NM_001169133	306451	Tenm3	2.351634	0.918319
NM_001037492	641603	Slc41a3	2.350756	0.841421
NM_001106263	292935	Htatip2	2.345952	0.811639
NM_001168285	498022	RGD1559482	2.342566	0.880239
NM_001131001	25441	Fcer1g	2.340353	0.92711
NM_001105928	288659	Vps37b	2.338265	0.915612
NM_019197	29366	Serpine2	2.338252	0.928903
NM_001271189	100360582	Nt5c	2.337938	0.892194
NM_001191882	363126	Nphp3	2.336991	0.818636
NM_001191568	681050	Pmf1	2.33342	0.87834
NM_001008722	292060	Irf8	2.312642	0.801336
NM_173136	286921	Akr1b8	2.309822	0.901617
NM_031807	83684	Tpbg	2.30934	0.82936
NM_001017464	361658	Btbd16	2.30526	0.823347
NM_001191791	315496	Dpy19l1	2.299648	0.920394
NM_017035	24655	Plcd1	2.296384	0.91737
NM_001013970	303378	Slfn13	2.290274	0.918214
NM_001109648	691750	Tmem107	2.287248	0.845956
NM_139342	246240	Ripk3	2.279764	0.875105
NM_001006975	293749	Ms4a6bl	2.275948	0.873523
NM_001013219	362924	St3gal1	2.274233	0.906083
NM_001109478	685679	Nme4	2.258256	0.849578
NM_017196	29427	Aif1	2.255933	0.914451
NM_019272	29745	Sema4f	2.252781	0.865858
NM_013151	25692	Plat	2.252724	0.925281
NM_001107225	305438	Sorcs2	2.247959	0.920007
NM_053843	116591	Fcgr2a	2.245334	0.812764
NM_001025046	500102	Fam131b	2.243784	0.831505
NM_148890	259272	Msi1	2.23949	0.815893
NM_001044257	498547	Epsti1	2.238327	0.808966
NM_001013149	308795	Mesdc1	2.230395	0.914346
NM_021578	59086	Tgfb1	2.226733	0.920851
NM_001109281	500762	Tmem198b	2.224681	0.897046

NM_001004095	445415	S100a11	2.217351	0.925492
NM_173101	25484	Myo1e	2.215849	0.916737
NM_001107205	305142	Abcg3l3	2.208401	0.890225
NM_173313	286978	Tmsb11	2.20707	0.901406
NM_053489	85251	Col18a1	2.207044	0.923383
NM_080782	114851	Cdkn1a	2.207035	0.907419
NM_171991	25203	Ccnb1	2.203407	0.828833
NM_001085381	266706	Gjc1	2.202957	0.887342
NM_013044	25566	Tmod1	2.19761	0.874437
NM_031107	81771	Rps6ka1	2.195206	0.884951
NM_001015020	316742	Tgif1	2.194877	0.887553
NM_001013214	362556	Ttc4	2.189794	0.881821
NM_130409	155012	Cfh	2.189554	0.90116
NM_012771	25211	Lyz2	2.188219	0.924262
NM_001108076	314654	Myo1f	2.178048	0.818776
NM_001108587	362187	Ccdc34	2.176258	0.851617
NM_001108214	316351	Npas2	2.172042	0.869655
NM_001044265	499991	Steap4	2.169604	0.920921
NM_013031	25549	Slc18a2	2.166338	0.871695
NM_001106461	295342	Rhoc	2.161205	0.922996
NM_001008366	361416	Cenpn	2.161119	0.802743
NM_001101011	64539	Ndufv3	2.1592	0.809951
NM_001169578	291006	Diras2	2.148619	0.863783
NM_017029	24588	Nefm	2.147008	0.887236
NM_012843	25314	Emp1	2.140066	0.92398
NM_017187	29395	Hmgb2	2.138928	0.910584
NM_013113	25650	Atp1b1	2.135395	0.877321
NM_001170563	306790	Sema4d	2.12941	0.817932
NM_021587	59107	Ltbp1	2.128252	0.905556
NM_012777	25239	Apod	2.124505	0.924508
NM_173114	286894	Parm1	2.12109	0.885162
NM_001106127	291355	Prtfdc1	2.119891	0.912342
NM_001047084	289076	Rgs18	2.116816	0.830591
NM_212525	361537	Tyrobp	2.109704	0.917335
NM_013025	25542	Ccl3	2.107496	0.831048
NM_001108426	361308	Kif20a	2.089042	0.864451
NM_001106422	294917	Tnik	2.088202	0.842932
NM_001024990	302972	Amdhd2	2.081218	0.881786
NM_001014269	367314	Lrrfip1	2.080646	0.900422
NM_001030027	290842	Gins4	2.077175	0.860654
NM_181634	314614	Lppr3	2.075631	0.90116
NM_001007672	303356	Tmem98	2.074987	0.876582
NM_023965	66021	Cybb	2.074448	0.848945
NM_017203	29454	Gdf11	2.069634	0.90429

NM_053332	80848	Cubn	2.068403	0.82718
NM_001108854	363984	Ikbke	2.068259	0.80974
NM_001025155	502715	Lrrc17	2.063904	0.885935
NM_001002807	406864	Clic1	2.060591	0.92057
NM_133624	171164	Gbp2	2.05922	0.916632
NM_198764	306417	Sh3rf1	2.058761	0.825387
NM_017113	29143	Grn	2.036321	0.863889
NM_001005881	300443	Lppr2	2.029063	0.883474
NM_001008524	362634	C1qc	2.023508	0.916632
NM_001127531	302671	Ap1s2	2.023118	0.896449
NM_001108385	361069	Dmtn	2.01225	0.889205
NM_001108664	362516	Msantd3	2.012082	0.87718
NM_001169138	292406	Thbs2	2.00596	0.909283
NM_031344	83512	Fads2	1.997741	0.918847
NM_001014787	304979	Igsf8	1.997079	0.905907
NM_001005539	294422	Smpdl3a	1.995421	0.903129
NM_001008278	287278	Cdkn2aipnl	1.992521	0.908087
NM_138538	171574	Dnm3	1.992329	0.868987
NM_001077673	362412	Rad18	1.991379	0.84455
NM_053598	94267	Nudt4	1.976057	0.918284
NM_001109327	501841	Coro1c	1.972546	0.913537
NM_203337	363040	St3gal4	1.969703	0.912975
NM_053749	114592	Aurkb	1.965472	0.830977
NM_172045	259225	Ppp1r14b	1.963663	0.916034
NM_001106607	297433	Podxl2	1.951765	0.892475
NM_207601	302562	Plp2	1.945311	0.918143
NM_001191737	295654	Ttc21b	1.942926	0.852286
NM_022205	60628	Cxcr4	1.942115	0.82711
NM_001048047	682105	Reep2	1.941458	0.883368
NM_021576	58813	Nt5e	1.941122	0.871695
NM_019138	25429	Cyp7b1	1.938255	0.823734
NM_021859	60450	Matk	1.937944	0.816245
NM_001100778	399489	E2f1	1.930494	0.851336
NM_001106074	290655	Crlf1	1.929082	0.917546
NM_012529	24264	Ckb	1.921765	0.918636
NM_138850	192203	Fap	1.918148	0.884951
NM_001012106	313702	Mad2l2	1.915897	0.880837
NM_001191558	307845	Mtss1l	1.915224	0.91526
NM_001134980	313373	Cyp2j10	1.909469	0.893671
NM_019336	54289	Rgs1	1.907957	0.840049
NM_001202463	360655	Cd300le	1.903713	0.878622
NM_031530	24770	Ccl2	1.88879	0.911498
NM_001100635	25119	Mr1	1.881004	0.862764
NM_012673	24832	Thy1	1.880336	0.900844

NM_001107376	307270	Me2	1.871428	0.883615
NM_001008768	246327	Prim1	1.868694	0.878235
NM_021835	24516	Jun	1.865367	0.916139
NM_001130577	500906	Cyth4	1.858026	0.834353
NM_012862	25333	Mgp	1.849613	0.917546
NM_001025137	498256	Ier5	1.837659	0.898558
NM_001004089	361710	Sipa1	1.829112	0.905239
NM_001102408	304944	Uck2	1.821333	0.856927
NM_001012061	308113	Cnksr3	1.819266	0.897046
NM_012733	25056	Rbp1	1.815785	0.910267
NM_001204879	100534597	Apitd1	1.815503	0.83801
NM_001004087	360942	Pcdh7	1.815453	0.815717
NM_001107400	307540	Celf4	1.811863	0.881716
NM_001013137	306748	Cxcl14	1.810201	0.892264
NM_001100735	361467	Lpcat1	1.802104	0.888819
NM_022226	63865	Lgmn	1.796243	0.906153
NM_001107489	308430	Zfp575	1.789413	0.812658
NM_001025119	310674	Plekho1	1.786399	0.899859
NM_017198	29431	Pak1	1.781225	0.854993
NM_017258	29618	Btg1	1.781032	0.911814
NM_001108902	365042	Rab32	1.780513	0.84782
NM_001008556	306808	Ippk	1.779955	0.806188
NM_001004274	360622	Igfbp4	1.777052	0.911217
NM_001108065	314612	Shc2	1.772959	0.860759
NM_001108452	361422	Cotl1	1.772439	0.911463
NM_001013995	305882	Haus4	1.771984	0.812764
NM_139341	246239	Slc15a3	1.770367	0.8359
NM_001108480	361567	Bcl2l12	1.769266	0.820534
NM_001013220	362956	Triobp	1.768959	0.902883
NM_001109300	501065	Cmtm7	1.767696	0.901582
NM_001134979	312299	Ezh2	1.758593	0.850809
NM_031348	83517	Fcna	1.757774	0.801723
NM_001106710	298845	Emilin1	1.755237	0.870429
NM_053931	116728	5-Sep	1.753374	0.833931
NM_017124	29185	Cd37	1.749547	0.864627
NM_001099647	685045	Abracl	1.749194	0.90443
NM_199093	295703	Serping1	1.747457	0.914065
NM_024160	79129	Cyba	1.746685	0.904184
NM_001013230	363930	Selplg	1.746319	0.814838
NM_001024771	305451	Tnip2	1.742399	0.8468
NM_001109508	688721	Lrfn4	1.740581	0.8859
NM_001024279	448830	Ly96	1.726896	0.87173
NM_001030026	290644	Ifi30	1.726783	0.902637
NM_001012164	361383	Cd97	1.725733	0.885443

NM_053563	89827	Ddx39a	1.724176	0.853692
NM_001005892	366126	Spi1	1.722545	0.863045
NM_001169101	266802	Alk	1.720487	0.805802
NM_001126287	361790	Ppp1r18	1.718178	0.904325
NM_001109584	690366	Vangl1	1.716444	0.887869
NM_212508	361791	Nrm	1.714493	0.869972
NM_001106268	292999	Chsy1	1.7141	0.874262
NM_001107163	304655	Zswim4	1.712262	0.839205
NM_019331	54281	Furin	1.711531	0.897609
NM_031140	81818	Vim	1.709732	0.912482
NM_001107137	304423	Tyw1	1.708894	0.875668
NM_017015	24434	Gusb	1.704772	0.874297
NM_001107716	310784	Taf13	1.703759	0.887869
NM_183330	252929	Ctsz	1.701405	0.899965
NM_017125	29186	Cd63	1.699857	0.912025
NM_001108550	361945	Postn	1.697451	0.87493
NM_012922	25402	Casp3	1.694867	0.874086
NM_001107524	308739	Rgma	1.691482	0.891104
NM_053586	94194	Cox5b	1.689207	0.9109
NM_001004226	295231	Glmp	1.681663	0.904325
NM_022597	64529	Ctsb	1.678145	0.91199
NM_001011898	288077	Hcls1	1.670927	0.808439
NM_001106173	291914	Cnep1r1	1.670022	0.89898
NM_001107977	313575	Hey1	1.665455	0.831927
NM_198767	308776	Pde8a	1.664127	0.866456
NM_001134883	314941	Lrp12	1.661916	0.87493
NM_022538	64369	Ppap2a	1.658421	0.907208
NM_053480	85242	Pola2	1.657448	0.808263
NM_152790	260416	Carhsp1	1.655257	0.904395
NM_053343	83825	Dclk1	1.654325	0.842722
NM_139096	245955	Lgals3bp	1.651202	0.902075
NM_001127337	499073	Brsk1	1.648074	0.837096
NM_001107821	311821	Agpat2	1.647346	0.87943
NM_001034032	619393	Dnajc12	1.647336	0.814803
NM_001109422	680611	Bcl3	1.64681	0.878376
NM_001271027	360959	Htra3	1.646786	0.897679
NM_001107124	304315	Ttyh3	1.64404	0.883298
NM_053727	114519	Nfil3	1.641788	0.835513
NM_012580	24451	Hmox1	1.641494	0.869444
NM_001130988	681062	Slbp	1.639623	0.858966
NM_001108210	316325	Hs6st1	1.630631	0.89884
NM_001034188	309651	Ppil1	1.630064	0.866913
NM_001105814	287537	Abr	1.628933	0.889487
NM_030847	81505	Emp3	1.627955	0.906118

NM_001014145	361237	Cdyl	1.627513	0.837131
NM_001025715	360646	Limd2	1.627116	0.881013
NM_001047851	292073	Galns	1.626347	0.829571
NM_001025721	361289	Colec12	1.624262	0.893495
NM_001008321	299626	Gadd45b	1.621779	0.896624
NM_012588	24484	Igfbp3	1.618484	0.884248
NM_001004203	288532	Mcm7	1.617244	0.86315
NM_001109291	500987	H2afx	1.616262	0.891913
NM_017195	29423	Gap43	1.614778	0.841069
NM_198755	301097	Vwa5a	1.614156	0.858333
NM_031092	81756	Rab13	1.613291	0.889135
NM_001107100	304021	Col8a1	1.612421	0.896238
NM_001108726	362800	Itgb8	1.611072	0.902602
NM_001079941	362095	Gtf3c5	1.6109	0.884318
NM_001014236	364534	Ssbp4	1.603999	0.890049
NM_053484	85246	Gas7	1.602613	0.906118
NM_001005891	362431	Clec4a3	1.602512	0.834599
NM_013085	25619	Plau	1.601124	0.84898
NM_001107153	304579	Usp30	1.599268	0.869444
NM_053786	116468	Rfc2	1.594886	0.810021
NM_001008379	363004	Prr13	1.59478	0.901688
NM_017154	497811	Xdh	1.594596	0.858439
NM_199382	296973	Bpgm	1.594414	0.898558
NM_001271080	292735	Shkbp1	1.591868	0.850035
NM_053765	114711	Gne	1.590354	0.868003
NM_139340	246237	Plekha5	1.589013	0.881224
NM_153318	266689	Cyp4f6	1.588205	0.822961
NM_012987	25491	Nes	1.585106	0.906013
NM_001100538	298510	Pabpc4	1.581818	0.891174
NM_145789	252963	Il13ra1	1.579537	0.868143
NM_001011893	287606	4-Sep	1.577318	0.850949
NM_001013236	685579	Rrm1	1.575185	0.873664
NM_001108381	361054	Ebpl	1.565725	0.815155
NM_001037768	297393	Nagk	1.559759	0.881083
NM_130411	155151	Coro1a	1.557503	0.845956
NM_012567	24392	Gja1	1.554529	0.897714
NM_053959	117028	Bin1	1.550413	0.893179
NM_001012064	308417	Pvrl2	1.550096	0.853446
NM_001012201	363058	Cadm1	1.546101	0.881962
NM_001012046	306141	Spry2	1.54526	0.888115
NM_017218	29496	Erbb3	1.544184	0.900563
NM_001024745	291733	Slc39a6	1.543145	0.881048
NM_017237	29545	Uchl1	1.54274	0.900949
NM_001014200	362828	Sppl2b	1.542381	0.887623

NM_023985	78969	Trib1	1.541812	0.83917
NM_001108313	360697	Ifngr2	1.5373	0.887623
NM_001025418	315648	Ppp2r1b	1.536224	0.857595
NM_001008508	288719	Tpst2	1.535683	0.887693
NM_053354	84350	Dnmt1	1.53462	0.841421
NM_130400	24312	Dhfr	1.534484	0.857068
NM_133380	25084	Il4r	1.532781	0.848558
NM_001079894	361659	Plekha1	1.53248	0.894902
NM_001002822	406196	Cchcr1	1.529495	0.824121
NM_173102	29214	Tubb5	1.526378	0.90545
NM_001007710	315939	Pxylp1	1.525174	0.865752
NM_001107657	310178	Myo10	1.522761	0.829184
NM_001108740	362859	Ckap4	1.522403	0.893495
NM_001025675	307351	Tubb6	1.521118	0.894444
NM_138892	192252	Dctpp1	1.520848	0.836779
NM_001108513	361689	Unc93b1	1.520488	0.877426
NM_001012044	306071	Lcp1	1.520293	0.878235
NM_012923	25405	Ccng1	1.520267	0.896132
NM_001037660	652957	Csf2ra	1.519824	0.866421
NM_017147	29271	Cfl1	1.519446	0.90538
NM_001007007	361056	Rnaseh2b	1.514103	0.859564
NM_181550	113894	Sqstm1	1.512794	0.827426
NM_001134572	361224	RGD1306058	1.510748	0.895499
NM_001100655	289786	Nacad	1.508509	0.819831
NM_001271261	554172	Pxdn	1.508393	0.881751
NM_001108747	362886	Rassf3	1.508047	0.887307
NM_001108815	363447	Tspan7	1.507604	0.885443
NM_023969	66025	Lpar3	1.505354	0.862271
NM_017066	24924	Ptn	1.504665	0.896519
NM_131902	54238	Cdkn2c	1.502348	0.806751
NM_177419	363247	Xrcc5	1.499813	0.866526
NM_001044287	685433	Evi2a	1.499208	0.837342
NM_133387	170898	Tmlhe	1.496416	0.819796
NM_012940	25426	Cyp1b1	1.493082	0.859177
NM_012904	25380	Anxa1	1.490853	0.899086
NM_019252	29640	Dpm2	1.485695	0.8782
NM_001008336	303882	Tnk2	1.484068	0.88147
NM_001108331	360785	Ap1s1	1.478752	0.888537
NM_001173341	305373	Hmgb3	1.478397	0.862482
NM_012924	25406	Cd44	1.477213	0.890823
NM_175756	289211	Fcgr2b	1.476742	0.843249
NM_001106215	292401	Smoc2	1.475539	0.891983
NM_138509	114764	Mapre1	1.47112	0.891842
NM_001134414	290501	Tmtc4	1.470337	0.855907

NM_033651	29728	Mcm4	1.468702	0.839873
NM_030826	24404	Gpx1	1.468118	0.898031
NM_001109612	690845	Rps4y2	1.467937	0.83147
NM_001039609	310839	Fnbp1l	1.467667	0.848101
NM_001107279	306055	Pcdh17	1.466288	0.832947
NM_001008300	291553	Nedd4l	1.464657	0.871941
NM_031318	83462	Dynlt1	1.463092	0.89135
NM_019141	25565	Tle4	1.462531	0.814311
NM_181478	353252	Rdh10	1.462393	0.888608
NM_001004215	291463	Ppic	1.459195	0.883544
NM_001013146	25446	Fosl2	1.456895	0.812377
NM_001005563	362941	Fbxl6	1.452866	0.825668
NM_001108068	314619	Sbno2	1.450532	0.852567
NM_001108750	362988	Cpne8	1.445704	0.842194
NM_012950	25439	F2r	1.445473	0.855239
NM_017253	29592	Bcat1	1.444884	0.866245
NM_139259	246143	Nradd	1.443316	0.845042
NM_021868	60465	Cttn	1.443258	0.893987
NM_001106998	303113	Jade2	1.443011	0.85661
NM_001006956	288449	Katnal1	1.442579	0.828762
NM_134373	171386	Avpi1	1.441354	0.887729
NM_001002851	289380	Nenf	1.440073	0.894023
NM_001114939	502642	RGD1563348	1.438281	0.863819
NM_001191840	84574	Tgfb1i1	1.437361	0.882841
NM_001004202	287910	Ccl6	1.434436	0.877918
NM_001107979	313582	Fhl3	1.428275	0.878165
NM_012918	25398	Cacna1a	1.424443	0.809775
NM_134334	171293	Ctsd	1.423948	0.899543
NM_001134640	502525	Dnajc19	1.421916	0.857278
NM_001107695	310661	Vps72	1.421235	0.880415
NM_053908	116689	Ptpn6	1.421092	0.825633
NM_001005543	298441	Nasp	1.420166	0.828973
NM_138894	192254	Grasp	1.418832	0.81199
NM_019316	54264	Mafb	1.417416	0.825211
NM_001006989	301013	Shisa5	1.41152	0.894093
NM_030842	81008	Itga7	1.408935	0.894796
NM_001108099	314856	Mdm2	1.408738	0.862377
NM_001013127	304983	Tagln2	1.408493	0.899226
NM_001170435	293155	Anapc15	1.407397	0.852496
NM_012555	24356	Ets1	1.398202	0.867862
NM_001106098	290959	Slc35d2	1.396402	0.824262
NM_001108617	362316	Cdk14	1.395167	0.823277
NM_138828	25728	Apoe	1.394527	0.885302
NM_030989	24842	Tp53	1.394114	0.886639

NM_001025406	294975	Exosc9	1.392286	0.860795
NM_001108308	360667	Mfsd11	1.389064	0.820113
NM_080885	140908	Cdk5	1.384659	0.863643
NM_153296	261730	Aurka	1.378515	0.82398
NM_001109376	679692	Lpgat1	1.378511	0.838748
NM_001100970	305494	Aebp1	1.377688	0.895992
NM_001001504	246770	Gtf2ird1	1.370226	0.822855
NM_182821	306203	Pxk	1.37003	0.860865
NM_001108866	364380	Abhd4	1.366714	0.893671
NM_001010953	303553	Rnd2	1.366615	0.8718
NM_001106210	292306	Rnaset2	1.365232	0.890155
NM_021744	60350	Cd14	1.364988	0.828129
NM_001012174	361810	Fkbp5	1.36393	0.860689
NM_001106700	298693	Isg15	1.36254	0.867229
NM_138839	192129	Vmp1	1.362049	0.885478
NM_001277055	310738	Ngf	1.36118	0.829641
NM_001106989	302993	Sox8	1.360826	0.867897
NM_001100518	292155	Hs2st1	1.360059	0.873945
NM_017059	24887	Bax	1.358344	0.876371
NM_001014135	360950	Wdr1	1.3583	0.892335
NM_001191792	315579	Ubash3b	1.356317	0.807489
NM_001013062	445442	Thbs1	1.355466	0.883439
NM_001127529	301525	Glb1l	1.35274	0.851266
NM_022278	64045	Glrx	1.352551	0.81308
NM_001107293	306261	Eaf1	1.351733	0.823242
NM_053827	116552	Plod1	1.350062	0.89026
NM_001012470	314329	Irf2bpl	1.349907	0.881786
NM_001107636	309837	Adamts14	1.349355	0.828024
NM_001106377	294291	RGD1564450	1.348831	0.850598
NM_175754	25592	Agrn	1.34756	0.870394
NM_022512	64304	Acads	1.345469	0.849754
NM_001037185	295107	Smc4	1.345453	0.851899
NM_022260	64026	Casp7	1.34215	0.8423
NM_001106033	290212	Efs	1.334719	0.875774
NM_001037775	304322	Chst12	1.334199	0.878586
NM_001013984	304860	Npl	1.334088	0.857525
NM_033443	25227	Arsb	1.330418	0.885091
NM_001173511	312083	Ccdc132	1.32928	0.836885
NM_031024	81653	Dbn1	1.325973	0.884318
NM_001106281	293118	Prcp	1.324527	0.865717
NM_053287	24390	B4galt1	1.324436	0.847293
NM_001013182	360492	Hn1l	1.321966	0.852286
NM_001103351	303905	Parp9	1.321346	0.858087
NM_153302	266605	Dcps	1.317234	0.828973

NM_198740	294273	RT1-DMb	1.316148	0.861463
NM_001134514	295678	Sestd1	1.312374	0.823769
NM_001100730	360971	Nipsnap1	1.306274	0.839135
NM_001100585	309153	Rce1	1.305152	0.834916
NM_133427	171015	Cyb5r4	1.304133	0.81744
NM_001007144	298199	Plin2	1.303841	0.875211
NM_199372	287436	Eif4a1	1.303479	0.890014
NM_053417	84477	Gab2	1.303027	0.857665
NM_012775	29591	Tgfbr1	1.300794	0.857068
NM_001025634	290500	Ggact	1.298254	0.811814
NM_001109150	499205	Fam181b	1.295419	0.835232
NM_198747	298101	Col27a1	1.29525	0.840471
NM_016990	24170	Add1	1.29478	0.889557
NM_024384	79227	Thoc6	1.294555	0.816245
NM_001008363	360772	Zfand2a	1.292188	0.841456
NM_001106876	301119	Mllt1	1.292154	0.872433
NM_001134863	306459	Stox2	1.290595	0.827672
NM_001106688	298544	Sh3bgrl3	1.28896	0.888889
NM_001108353	360914	Plac8	1.287924	0.863678
NM_001135743	368084	Wbscr22	1.287619	0.807243
NM_024156	79125	Anxa6	1.287384	0.89012
NM_001191807	301415	Ankrd44	1.285638	0.817264
NM_001035255	502603	Srsf11	1.28474	0.883439
NM_022511	64303	Pfn1	1.28378	0.891315
NM_001047854	293628	Chid1	1.283596	0.838854
NM_053593	94201	Cdk4	1.281779	0.88474
NM_199404	361378	Man2b1	1.280481	0.873875
NM_022674	58940	H2afz	1.279091	0.875563
NM_001013944	299153	Ppp1r36	1.276975	0.877075
NM_053445	84575	Fads1	1.275083	0.887729
NM_019621	29495	Dlg4	1.274317	0.864487
NM_001106913	301434	Clk1	1.273548	0.885478
NM_001126272	303164	Zfp692	1.271911	0.841983
NM_001108683	362606	Sync	1.267539	0.802145
NM_001106576	296758	Armc10	1.265657	0.860513
NM_001107989	313662	Mfap2	1.264644	0.867475
NM_031986	83841	Sdcbp	1.263261	0.88474
NM_012755	25150	Fyn	1.26252	0.880556
NM_001106511	296156	Cpxm1	1.261588	0.850211
NM_053776	116456	Dnajc2	1.260388	0.862518
NM_012903	25379	Anp32a	1.259345	0.886568
NM_001106455	295325	Igsf3	1.258159	0.83045
NM_001108393	361103	Zmiz1	1.25638	0.879852
NM_001126091	680737	Snrpf	1.256077	0.877989

NM_019904	56646	Lgals1	1.255348	0.89128
NM_001135749	499655	Cks1b	1.255217	0.842511
NM_001170335	292721	Erf	1.254767	0.862201
NM_001106795	300259	Aaas	1.253731	0.822504
NM_001025279	306014	Reep4	1.253536	0.838256
NM_001009624	287598	Ska2	1.251996	0.827707
NM_001113791	501007	RGD1562618	1.251036	0.853833
NM_001008327	300797	Fam96a	1.249986	0.810373
NM_001013213	362548	Itgb3bp	1.247529	0.817405
NM_012793	25257	Gamt	1.246537	0.871273
NM_001109274	500616	Socs5	1.244693	0.866069
NM_001108192	316033	Glb1	1.242128	0.860302
NM_134353	171350	Pabpc1	1.238992	0.882419
NM_138548	191575	Nme1	1.238889	0.868073
NM_001107749	311209	Tp53i11	1.232763	0.87173
NM_001108676	362586	Trit1	1.232066	0.811357
NM_031721	65164	Htra1	1.229848	0.88154
NM_001106164	291813	Cmtm3	1.22979	0.855169
NM_012705	24932	Cd4	1.224799	0.838994
NM_145094	246324	Rab31	1.224038	0.865014
NM_030992	25096	Pld1	1.22163	0.831997
NM_001106286	293186	Lyve1	1.220736	0.845077
NM_001012110	313913	Zfp513	1.217885	0.841421
NM_001011946	294673	Hexb	1.21598	0.866878
NM_172222	24231	C2	1.214258	0.8641
NM_001136162	292095	Ttc13	1.209252	0.850633
NM_001106226	292622	Gltscr1	1.209235	0.848523
NM_001106285	293166	Insc	1.206225	0.820534
NM_130812	25164	Cdkn2b	1.205167	0.820992
NM_031342	83510	Lypla2	1.203788	0.858193
NM_019152	29153	Capn1	1.202895	0.86512
NM_001024268	81513	Lig1	1.202804	0.836709
NM_001106622	297602	Tapbpl	1.201966	0.840928
NM_001106430	295037	Mgst2	1.199688	0.840506
NM_012801	25266	Pdgfa	1.199364	0.87057
NM_001009629	288414	Rfc3	1.197463	0.812271
NM_001005876	287828	Hn1	1.197291	0.870745
NM_012618	24615	S100a4	1.189902	0.880696
NM_001191753	312846	Rassf8	1.189769	0.842968
NM_001100651	288041	Pigx	1.189547	0.844796
NM_001135085	681031	Snrpg	1.187953	0.869585
NM_001006969	292892	Irf3	1.186642	0.864241
NM_001008303	292729	Snrpa	1.186636	0.83256
NM_001034124	287382	Mfap4	1.186066	0.80102

NM_001009543	494346	Slmo2	1.186042	0.861428
NM_024147	79115	Evl	1.185685	0.84775
NM_031130	81808	Nr2f1	1.185022	0.862729
NM_001106852	300974	Mrpl3	1.182363	0.845253
NM_001127544	310791	Slc25a24	1.18202	0.842968
NM_001033683	287721	Vat1	1.181422	0.877672
NM_001106833	300803	Lactb	1.18012	0.802426
NM_001014120	360627	Fkbp10	1.180102	0.863467
NM_022529	64360	Mrpl23	1.1795	0.867089
NM_001100770	366001	Arrdc1	1.17587	0.822222
NM_130744	170520	Cygb	1.173953	0.86635
NM_022242	63912	Fam129a	1.169311	0.863537
NM_001126274	305458	Tmem129	1.168903	0.80756
NM_145778	252921	Tubg1	1.168385	0.820218
NM_001009271	290558	Nt5dc2	1.167026	0.845675
NM_001025125	360800	Sumf2	1.166766	0.834529
NM_053943	116782	Pcdhgc3	1.166062	0.831364
NM_021581	59101	Leprel4	1.162033	0.83903
NM_001106097	290939	Med10	1.160417	0.856997
NM_001008521	315265	Twf1	1.160158	0.866385
NM_001014125	360722	Pdia5	1.159596	0.809599
NM_001109433	680945	Sdf2l1	1.159025	0.821554
NM_031574	29372	Rasa3	1.158301	0.844796
NM_057213	117596	Atp6v1b2	1.157563	0.847539
NM_001013191	361391	Cbfb	1.155703	0.845042
NM_001103352	307644	Kifc3	1.153774	0.819374
NM_001244867	691657	Cripl	1.151203	0.871765
NM_031012	81641	Anpep	1.151001	0.831188
NM_053374	84388	Il18bp	1.149809	0.81737
NM_001004238	299799	Rab21	1.149395	0.86621
NM_001105781	287302	Mrpl22	1.145185	0.82282
NM_001106163	291794	Snrpd1	1.145017	0.862588
NM_001007744	362112	Tor2a	1.144102	0.817932
NM_053616	113929	Nup88	1.143288	0.820288
NM_001191577	314623	Midn	1.142561	0.859529
NM_001108818	363485	Pbdc1	1.141598	0.830767
NM_022402	64205	Rplp0	1.140316	0.878059
NM_001106899	301337	Plekhb2	1.135799	0.866526
NM_001106219	292536	Prpf31	1.135116	0.80429
NM_001191675	289382	Ints7	1.133122	0.800035
NM_139336	246232	Uxs1	1.132479	0.858404
NM_001012167	361527	Pld3	1.131945	0.867229
NM_001106612	297481	Eif4e3	1.131492	0.832243
NM_001135020	689134	Sec61g	1.128805	0.870429

NM_019143	25661	Fn1	1.124431	0.875668
NM_130413	155183	Skap2	1.124111	0.841632
NM_175761	299331	Hsp90aa1	1.123356	0.87493
NM_001108630	362374	Vopp1	1.122452	0.833544
NM_001014090	315594	Oaf	1.120059	0.871624
NM_001106353	293939	Erlin1	1.119009	0.814205
NM_001108530	361831	Ube2d1	1.118403	0.824578
NM_001100493	287134	Gnptg	1.118197	0.848734
NM_001013870	288914	Trmt1	1.118181	0.813678
NM_130416	155423	Anxa7	1.11532	0.866913
NM_012875	25347	Rpl39	1.113522	0.875035
NM_021740	29222	Ptma	1.113273	0.875633
NM_053990	117063	Ptpn2	1.112904	0.80974
NM_001013157	310378	Nnt	1.110904	0.816737
NM_212498	361789	Atat1	1.108385	0.846976
NM_001244855	362965	Rangap1	1.108233	0.832771
NM_173116	286896	Sgpl1	1.105553	0.847152
NM_001126288	362792	Pld4	1.104624	0.828305
NM_001106600	297381	Wbp1	1.104523	0.864838
NM_001047885	310326	Arse	1.104192	0.830942
NM_001106654	298068	Sec61b	1.103988	0.860443
NM_019905	56611	Anxa2	1.103835	0.875281
NM_145878	140868	Fabp5	1.103569	0.85879
NM_001037199	313689	Dhrs3	1.100269	0.864768
NM_021261	50665	Tmsb10	1.097947	0.875985
NM_053607	94340	Acsl5	1.096631	0.8109
NM_001108296	360617	Mien1	1.096545	0.860443
NM_001167666	100312984	Eif2s3y	1.096008	0.856751
NM_012500	24206	Apeh	1.095131	0.829079
NM_001013186	361065	Bin3	1.095076	0.838291
NM_001115022	289831	Commd1	1.093759	0.852918
NM_001191693	307178	Arhgap21	1.090194	0.825598
NM_001130564	362923	Efr3a	1.086571	0.844163
NM_001025041	499772	Ier5l	1.086504	0.844409
NM_031723	65166	Sec11a	1.086206	0.860689
NM_031838	83789	Rps2	1.085765	0.875281
NM_019263	29688	Minpp1	1.084382	0.83917
NM_001024269	360503	Luc7l	1.084101	0.8173
NM_001002831	406230	Rpp21	1.083368	0.857736
NM_001004231	296562	Npdc1	1.083272	0.873207
NM_053439	84509	Ran	1.081979	0.865823
NM_031135	81813	Klf10	1.080408	0.834951
NM_001106116	291206	Mrpl32	1.080314	0.825246
NM_053453	84583	Rgs2	1.079479	0.810759

NM_001013105	300211	Fkbp11	1.079279	0.808615
NM_022399	64202	Calr	1.079064	0.874684
NM_031510	24479	Idh1	1.077741	0.847961
NM_030995	25152	Mapla	1.077693	0.847961
NM_001108865	364240	Dnajc9	1.077244	0.833931
NM_031137	81815	Tpp2	1.076708	0.829325
NM_019275	50554	Smad4	1.075606	0.858087
NM_012802	25267	Pdgfra	1.075317	0.857173
NM_021661	59293	Rgs19	1.073918	0.802602
NM_001108307	360664	Ten1	1.072104	0.843987
NM_017249	29579	Esyt1	1.071985	0.853692
NM_001115024	290994	Lman2	1.071522	0.862096
NM_001047856	294030	Sfr1	1.069648	0.864557
NM_053696	114488	Rbm3	1.068989	0.867827
NM_001007756	363462	Gemin8	1.067797	0.817897
NM_145781	252928	Timm13	1.067728	0.847539
NM_198732	291339	Commd3	1.067353	0.864592
NM_001105933	288669	Arpc3	1.066448	0.869902
NM_001008348	309172	Frmd8	1.066379	0.862834
NM_012620	24617	Serpine1	1.066289	0.834529
NM_001106950	302495	Rap2c	1.066214	0.838608
NM_153469	266709	Pkig	1.064424	0.851231
NM_138524	171553	A3galt2	1.062652	0.841421
NM_001107600	309444	Fbxw4	1.061784	0.822644
NM_001109442	681415	Erh	1.061544	0.843143
NM_001025420	361680	Lsp1	1.061143	0.839065
NM_053849	116598	Pdia4	1.060529	0.855028
NM_001271090	25603	Marcks	1.058732	0.861498
NM_001025718	361178	Tfdp1	1.057169	0.822187
NM_001014255	365872	Aph1a	1.056261	0.841702
NM_181091	113940	Gmfg	1.054063	0.801758
NM_023981	78965	Csf1	1.053396	0.830942
NM_001009962	287151	Metrn	1.05017	0.860338
NM_001143858	309081	Dock1	1.048989	0.834177
NM_053628	114029	Megf8	1.048903	0.81737
NM_080910	140946	Paics	1.048783	0.85327
NM_053819	116510	Timp1	1.047064	0.861181
NM_199256	80843	Sec61a1	1.046184	0.854079
NM_001047111	499941	Dbndd2	1.044898	0.85116
NM_001024368	501665	Slc10a3	1.04268	0.81526
NM_001012356	317259	Nono	1.042621	0.85879
NM_001128083	688785	Trim8	1.041498	0.850035
NM_001077200	361430	Piezo1	1.041416	0.816983
NM_021694	60323	Arhgef1	1.039908	0.842511

NM_001011936	293524	Bag3	1.039675	0.842018
NM_001014219	363255	Ankzf1	1.039578	0.814451
NM_001007606	266975	Sars	1.038293	0.853551
NM_031645	58965	Ramp1	1.037453	0.85334
NM_001002016	60374	Lmna	1.035725	0.859423
NM_001012179	361927	Fxr1	1.032244	0.844831
NM_053512	85274	Prdx4	1.031783	0.847855
NM_001008765	303163	Igtp	1.031122	0.816245
NM_001004230	296304	Edem2	1.030664	0.808439
NM_001106196	292090	Galnt2	1.030321	0.853059
NM_001039378	287427	Trappc1	1.029783	0.852918
NM_133569	171100	Angptl2	1.02801	0.850387
NM_001100547	300980	Dusp7	1.026599	0.832243
NM_001127295	300088	Polr3h	1.026485	0.815647
NM_012562	24375	Fuca1	1.02619	0.850949
NM_001030039	315707	Csk	1.025979	0.842546
NM_001108592	362223	Snrpb2	1.025823	0.837236
NM_134359	171366	Ppp4c	1.025579	0.833404
NM_001106073	290647	Lsm4	1.021868	0.838045
NM_001024779	310848	Cyp2u1	1.018926	0.823031
NM_017248	29578	Hnrnpa1	1.01877	0.85647
NM_022381	25737	Pcna	1.016835	0.842616
NM_017169	29338	Prdx2	1.013641	0.858368
NM_001106999	303122	RGD1310352	1.012076	0.846484
NM_001107806	311671	Npepl1	1.011965	0.84135
NM_001106572	296708	Olfml2a	1.011218	0.847785
NM_053928	116725	Ube2n	1.010491	0.851793
NM_001047874	302247	RGD1304704	1.010322	0.854079
NM_053857	116636	Eif4ebp1	1.010262	0.846941
NM_199081	287642	Slc35b1	1.009476	0.843425
NM_001013239	366014	Zdhhc12	1.008421	0.805837
NM_001037652	361771	Zdhhc6	1.008067	0.816526
NM_031504	24233	C4a	1.007849	0.826688
NM_001106945	302422	Pdzd11	1.007373	0.822363
NM_001134861	300129	Cerk	1.006907	0.829184
NM_031797	83628	Cd82	1.005531	0.852743
NM_001004272	360550	Phf23	1.004291	0.821027
NM_001012504	307947	Set	1.003825	0.850422
NM_001017537	297392	Tex261	1.003646	0.850387
NM_134396	171433	Micu2	1.002892	0.8327
NM_001126098	691193	Elof1	1.001673	0.837342
NM_031121	81785	Ssrp1	1.001102	0.846941
NM_001079705	292139	RGD1311558	1.000904	0.842792
NM_138549	191576	Tecr	-1.00057	0.858861

NM_001024864	287061	Rogdi	-1.00306	0.815612
NM_001107026	303351	Rhot1	-1.00669	0.828622
NM_053304	29393	Col1a1	-1.0108	0.861181
NM_001011995	300218	Tuba1c	-1.01119	0.850985
NM_172317	116831	Fxyd3	-1.01343	0.836041
NM_031819	83720	Fat1	-1.01398	0.824051
NM_024360	29577	Hes1	-1.02458	0.804923
NM_001004209	289456	Hsd17b11	-1.02705	0.857208
NM_001107001	303132	Aff4	-1.033	0.815647
NM_001100576	307096	Akr1c19	-1.03327	0.824508
NM_001007601	29700	Pcbd1	-1.03335	0.822996
NM_019156	29169	Vtn	-1.03348	0.835935
NM_012656	24791	Sparc	-1.0335	0.863221
NM_053714	114506	Ankh	-1.03404	0.853586
NM_001109050	498014	RGD1565033	-1.03513	0.829008
NM_172336	282840	Atf5	-1.03519	0.857595
NM_001008364	361328	Snx24	-1.03712	0.824051
NM_022289	64088	Snx16	-1.03907	0.816034
NM_001014007	306766	LOC306766	-1.04065	0.853059
NM_022177	24772	Cxcl12	-1.04482	0.806927
NM_031524	24628	Pdgfb	-1.04617	0.815893
NM_020088	117242	Tenm2	-1.04783	0.810935
NM_001108725	362789	Zfyve21	-1.04809	0.809072
NM_022592	64524	Tkt	-1.05022	0.85872
NM_130403	114004	Ppp1r14a	-1.05402	0.8077
NM_001100706	311839	Slc27a4	-1.05509	0.847996
NM_001191606	294988	Ankrd50	-1.05758	0.808896
NM_001106009	289664	Ldb2	-1.06289	0.817089
NM_001115021	499022	Qk	-1.06688	0.871624
NM_001108273	360519	Rasgef1c	-1.06803	0.843706
NM_198738	293820	Psat1	-1.06807	0.827954
NM_001271344	100359680	Lpcat2	-1.06911	0.841913
NM_199403	360863	Suco	-1.07191	0.807595
NM_017000	24314	Nqo1	-1.07203	0.811639
NM_031549	25123	Tagln	-1.07273	0.87057
NM_001107051	303547	Ezh1	-1.0733	0.827532
NM_001106749	299207	RGD1310769	-1.07861	0.835127
NM_001009692	311848	Sh3glb2	-1.07889	0.867089
NM_001008360	360480	Cdip1	-1.0793	0.83801
NM_001009405	310833	Arhgap29	-1.08149	0.811287
NM_001044284	684980	Tsc22d4	-1.08178	0.86315
NM_001013912	295264	Mllt11	-1.08185	0.833474
NM_177425	29317	Csrp2	-1.08242	0.835267
NM_053878	116657	Cplx2	-1.08403	0.801828

NM_019249	360406	Ptprf	-1.08546	0.8577
NM_031701	65131	Cldn5	-1.0875	0.85334
NM_001038615	308099	Fndc1	-1.08787	0.815577
NM_138832	170567	Slc38a1	-1.08795	0.845745
NM_206950	404280	Midlip1	-1.09455	0.859001
NM_001170600	24582	Myh11	-1.09579	0.84121
NM_001105936	288740	Iscu	-1.09601	0.866491
NM_001014239	364637	Fbxo25	-1.09651	0.813889
NM_053772	114906	Pkia	-1.09981	0.857841
NM_013070	25600	Utrn	-1.10471	0.858615
NM_057211	117560	Klf9	-1.10651	0.859037
NM_001108007	313860	Pkdcc	-1.10674	0.847785
NM_001101680	171356	Foxc2	-1.1077	0.8327
NM_001015003	297498	Crbn	-1.1096	0.841807
NM_022501	338401	Crip2	-1.11281	0.873699
NM_001011917	290291	Cab391	-1.11337	0.856997
NM_012609	24592	Nf1	-1.1144	0.817053
NM_001191846	84482	Foxo1	-1.11527	0.818214
NM_012576	24413	Nr3c1	-1.11657	0.84789
NM_023090	29452	Epas1	-1.11917	0.865752
NM_001134970	691042	Sbf2	-1.11927	0.851899
NM_001127546	315131	Kdelr3	-1.11981	0.868354
NM_001281824	303638	Abca8a	-1.12448	0.873594
NM_013000	25508	Pam	-1.13376	0.871167
NM_001107301	306338	Abhd8	-1.13656	0.834951
NM_001106911	301388	Mfsd6	-1.13738	0.83597
NM_001014106	499328	Rfk	-1.13769	0.859248
NM_057100	58935	Gas6	-1.13903	0.871484
NM_001007697	310664	Prune	-1.14141	0.804782
NM_001012119	315136	Cbx6	-1.14149	0.858298
NM_001007656	298848	Mapre3	-1.14513	0.863467
NM_013191	25742	S100b	-1.14516	0.878938
NM_032085	84032	Col3a1	-1.14563	0.879008
NM_017086	25148	Egr3	-1.15112	0.819444
NM_012847	25318	Fnta	-1.15532	0.872433
NM_130829	170673	Palm	-1.15624	0.867475
NM_001025703	315160	Desi1	-1.15916	0.868214
NM_012807	25273	Smo	-1.15932	0.835021
NM_031771	83580	Thbd	-1.15967	0.852567
NM_001044294	689161	Gabarapl1	-1.15968	0.871343
NM_001108708	362731	Snx13	-1.16033	0.840471
NM_001127580	690163	Oxct1	-1.16169	0.835654
NM_012782	25244	Bckdha	-1.16237	0.848347
NM_057104	84050	Enpp2	-1.16587	0.847082

NM_001271232	362118	Mvb12b	-1.17401	0.873066
NM_017024	24530	Lcat	-1.17444	0.842757
NM_178091	288985	Insig2	-1.17742	0.802426
NM_172034	64511	Fntb	-1.17865	0.850668
NM_001007647	297337	Rnf181	-1.18302	0.861006
NM_053555	89818	Vamp5	-1.18384	0.81853
NM_017061	24914	Lox	-1.1902	0.830767
NM_019204	29392	Bace1	-1.19093	0.839241
NM_001107954	313488	Reck	-1.1918	0.859283
NM_001006991	305149	Nudt9	-1.19195	0.864557
NM_053657	114116	Cdc42bpa	-1.19363	0.833122
NM_053986	117057	Myo1b	-1.19717	0.861674
NM_053811	116501	Slc9a3r2	-1.19723	0.870675
NM_001126093	685448	Pcp4l1	-1.20005	0.841315
NM_001100789	498331	Ptpn13	-1.20583	0.84782
NM_012913	25390	Atp1b3	-1.20589	0.879149
NM_012792	25256	Fmo1	-1.21411	0.8218
NM_001037097	366697	Sptlc2	-1.21761	0.858404
NM_001013047	288762	Mtm1	-1.21958	0.81403
NM_130741	170496	Lcn2	-1.22081	0.845148
NM_001014136	361014	Ngly1	-1.22163	0.851231
NM_001034125	287422	Per1	-1.22468	0.843249
NM_001012121	315189	Prr5	-1.22974	0.853446
NM_001127565	500464	Epb41l4b	-1.23481	0.835127
NM_001109558	689954	Setd7	-1.23515	0.857841
NM_172062	64475	P4ha1	-1.23544	0.868284
NM_133581	171112	Wfdc1	-1.24163	0.873523
NM_001271384	316021	Epm2aip1	-1.24283	0.839838
NM_001109062	498153	Gpr146	-1.2497	0.814909
NM_001106217	292462	Lrp11	-1.25019	0.80429
NM_181368	290553	Mustn1	-1.25273	0.852461
NM_001025753	366232	Rem1	-1.2529	0.8077
NM_031753	79559	Alcam	-1.26097	0.851828
NM_001014140	361118	RGD1309676	-1.26281	0.812764
NM_001100973	307237	Pard6g	-1.26387	0.823312
NM_139110	245977	Gpr116	-1.26414	0.845218
NM_001107519	308668	Nipa1	-1.26473	0.801969
NM_053367	84380	Dhh	-1.27022	0.883228
NM_001106198	292098	Pgbd5	-1.27055	0.838397
NM_001025017	361992	Them4	-1.27367	0.807947
NM_001009292	293489	Slx1b	-1.27773	0.835091
NM_001012000	300900	Plscr4	-1.28675	0.84993
NM_001047869	299012	Arhgap5	-1.28772	0.842511
NM_031648	58971	Fxyd1	-1.28803	0.891069

NM_001034012	310670	Adamtsl4	-1.28845	0.864276
NM_031063	81727	Mvk	-1.28921	0.844233
NM_019318	54267	Maf	-1.28934	0.883509
NM_134338	364706	Foxc1	-1.28938	0.815084
NM_031022	81651	Cspg4	-1.29038	0.857454
NM_022543	64387	Ccdc80	-1.29058	0.860795
NM_033234	24440	Hbb	-1.29059	0.876512
NM_001109444	681578	Rnf13	-1.30042	0.8859
NM_001008334	303330	Tmem97	-1.30052	0.864416
NM_001007802	316035	Cmtm6	-1.30217	0.874297
NM_031509	24421	Gsta1	-1.30564	0.865928
NM_031783	83613	Nefl	-1.30624	0.853165
NM_001191910	140589	Gli1	-1.31004	0.869655
NM_001015027	362453	Crebl2	-1.3133	0.857208
NM_001134884	315174	Scube1	-1.31661	0.88045
NM_001107303	306344	Arrdc2	-1.3173	0.830345
NM_001100533	296583	Nacc2	-1.31866	0.872855
NM_170788	266806	Erc1	-1.31929	0.803762
NM_199117	362962	Cbx7	-1.32246	0.846308
NM_001105845	287745	Plcd3	-1.32608	0.847398
NM_199396	315259	Prickle1	-1.33039	0.859248
NM_001108831	363634	Kctd11	-1.33727	0.862166
NM_001108542	361882	Arhgef28	-1.34102	0.814522
NM_001134548	310362	RGD1305938	-1.34685	0.817053
NM_001109338	502421	Slc35f1	-1.34834	0.845148
NM_001004213	290796	Saraf	-1.34893	0.890225
NM_021266	58868	Fzd1	-1.35125	0.855661
NM_031011	81640	Amd1	-1.3535	0.870886
NM_138905	192270	Ppap2b	-1.3536	0.889909
NM_024396	79248	Abca2	-1.35888	0.888291
NM_001191682	305467	Sfi1	-1.36539	0.846449
NM_001004269	315509	Jam3	-1.36896	0.879536
NM_001100666	295105	Schip1	-1.37607	0.878903
NM_057137	117278	Ebp	-1.38031	0.865331
NM_001009620	287129	Tmem204	-1.38242	0.829817
NM_031752	78958	Bcam	-1.38296	0.889557
NM_001108006	313825	Vps13d	-1.38566	0.820745
NM_138843	192172	Mpst	-1.38672	0.853094
NM_001169103	298744	Criml	-1.38767	0.820745
NM_001106162	291793	Abhd3	-1.389	0.803692
NM_031765	83574	Rxrg	-1.39353	0.831716
NM_177421	305886	Slc22a17	-1.39406	0.894761
NM_001135778	680409	Prodh	-1.4	0.801793
NM_023957	66013	Arhgef9	-1.40336	0.839241

NM_031762	83571	Cdkn1b	-1.40972	0.894866
NM_012988	25492	Nfia	-1.41289	0.881646
NM_053573	93667	Olfm1	-1.41301	0.847328
NM_013149	25690	Ahr	-1.41317	0.847398
NM_019196	29365	Mpdz	-1.42643	0.865401
NM_001101005	689852	Psmf1	-1.42677	0.89448
NM_134336	171297	Nlgn3	-1.42865	0.814557
NM_001109447	683713	Gal3st1	-1.42926	0.85102
NM_031613	58814	Tmod2	-1.43323	0.877743
NM_001271081	308571	Lrrc4b	-1.43659	0.866632
NM_022005	63847	Fxyd6	-1.43851	0.900176
NM_001134754	362713	Seli	-1.4397	0.87289
NM_012504	24211	Atp1a1	-1.44226	0.895359
NM_001002830	305302	Rasl11b	-1.44727	0.804114
NM_001106468	295425	Usp53	-1.45164	0.835443
NM_139325	24334	Eno2	-1.45269	0.891526
NM_001191927	498081	Slc35a5	-1.45389	0.854571
NM_001034083	29140	Snn	-1.4554	0.891667
NM_022245	64001	Cyb5a	-1.45678	0.897996
NM_012716	25027	Slc16a1	-1.45909	0.884529
NM_207616	367166	Hyal1	-1.46073	0.845956
NM_001108509	361676	Pnpla2	-1.46931	0.890717
NM_013096	25632	Hba1	-1.47038	0.900211
NM_022224	63852	Pter	-1.47276	0.85218
NM_001109120	498741	Mboat1	-1.47405	0.874332
NM_145682	246776	Filip1	-1.47848	0.874543
NM_017149	29279	Meox2	-1.48061	0.870534
NM_012596	24536	Lepr	-1.48079	0.846343
NM_001048044	313838	Cdc42ep3	-1.48496	0.827426
NM_001108286	360590	Ypel2	-1.48612	0.872855
NM_001013192	361621	Olfml1	-1.48843	0.879817
NM_053889	116669	Vwf	-1.48876	0.831997
NM_001106120	291309	Usp6nl	-1.49224	0.88256
NM_001013244	366602	Tspan13	-1.49327	0.88685
NM_001008352	310645	Pmvk	-1.50472	0.887729
NM_001107698	310678	Hist2h3c2	-1.50664	0.821378
NM_177426	24424	Gstm2	-1.5073	0.894233
NM_053394	84410	Klf5	-1.50834	0.889381
NM_017221	29499	Shh	-1.5169	0.886744
NM_053796	116479	F11r	-1.51849	0.873101
NM_024388	79240	Nr4a1	-1.52072	0.899508
NM_030865	81523	Myoc	-1.53245	0.903833
NM_145089	246307	Asrgl1	-1.53445	0.882138
NM_022249	64015	Khdrbs3	-1.53803	0.845816

NM_001014043	310849	Sgms2	-1.53902	0.836181
NM_012600	24552	Me1	-1.54043	0.871765
NM_001105759	259237	Dock9	-1.54134	0.879852
NM_022856	64824	Nab1	-1.54597	0.872152
NM_001137643	499422	Gstt3	-1.55132	0.882384
NM_001108125	315427	Sesn3	-1.55139	0.875598
NM_031726	65171	Scamp5	-1.55349	0.872117
NM_001107188	304893	Rasal2	-1.56159	0.890682
NM_022712	64678	Tfrc	-1.56224	0.859072
NM_001011933	293173	Far1	-1.56276	0.891948
NM_178330	291946	Tmem184c	-1.56319	0.883158
NM_001013171	314543	Gulp1	-1.56388	0.879079
NM_013145	25686	Gnai1	-1.56572	0.886111
NM_001107024	303342	Ssh2	-1.56635	0.822609
NM_013217	26955	Mllt4	-1.56662	0.819726
NM_012608	24590	Mme	-1.57839	0.883826
NM_001108272	360511	Pank3	-1.58028	0.897714
NM_199109	314323	Flvcr2	-1.58224	0.843249
NM_001107940	313270	Megf9	-1.58646	0.901899
NM_001007722	360504	Hba2	-1.58654	0.904325
NM_001107200	305064	Ptpn14	-1.59058	0.856329
NM_001108621	362336	Fam180a	-1.59535	0.87398
NM_001191872	362734	Stxbp6	-1.59687	0.821132
NM_017256	29610	Tgfbr3	-1.6024	0.899402
NM_001107288	306204	Flnb	-1.60392	0.897574
NM_021863	60460	Hspa2	-1.60863	0.904606
NM_001271239	500921	Tmem117	-1.60912	0.878376
NM_001009391	305177	Enoph1	-1.60925	0.89673
NM_181474	308140	Tfb1m	-1.61788	0.887482
NM_031057	81708	Aldh6a1	-1.62391	0.89557
NM_053703	114495	Map2k6	-1.62581	0.832419
NM_001047957	500350	LOC500350	-1.62645	0.878094
NM_031582	29473	Aoc3	-1.62781	0.894023
NM_001008351	309681	RGD1309594	-1.63053	0.896976
NM_001033694	300095	Srebf2	-1.63361	0.895077
NM_001134696	100188934	Ctxn3	-1.6348	0.885373
NM_024364	60563	Hr	-1.64124	0.859423
NM_001271410	100360066	Evi5	-1.64213	0.896378
NM_001106306	293566	Cpxm2	-1.64618	0.896589
NM_013104	25641	Igfbp6	-1.65156	0.908087
NM_001033663	64363	Araf	-1.65396	0.846554
NM_019312	54260	Itpkb	-1.65767	0.89571
NM_001107626	309732	Ado	-1.6667	0.881083
NM_145096	246326	Zdhhc2	-1.66833	0.902321

NM_001108654	362481	Tox	-1.6764	0.823453
NM_001106082	290705	Nek1	-1.67738	0.882349
NM_001107764	311341	Pla2g4b	-1.6784	0.877778
NM_001135879	365924	Tmem56	-1.67931	0.864592
NM_001009966	311187	Pacsin3	-1.67985	0.876688
NM_013162	25703	Rbp4	-1.68414	0.849332
NM_017105	25667	Bmp3	-1.6904	0.806927
NM_001024371	553106	Ncald	-1.69832	0.831927
NM_001109010	367323	Nudt12	-1.70508	0.850527
NM_145784	252939	Gpr37l1	-1.71382	0.903305
NM_001109535	689377	Rab20	-1.71455	0.835513
NM_033653	29253	Maoa	-1.71584	0.899191
NM_019239	29582	Mgat3	-1.725	0.9
NM_031731	65183	Aldh3a2	-1.72707	0.895992
NM_053365	79451	Fabp4	-1.72858	0.906118
NM_001047085	289089	Ivns1abp	-1.72902	0.909705
NM_001001800	308900	rnf141	-1.73007	0.899086
NM_053492	85254	Slc44a1	-1.73017	0.898699
NM_053363	84360	Clcn3	-1.73132	0.898031
NM_175582	291023	Id4	-1.7336	0.883755
NM_181381	312382	Abcg2	-1.73492	0.841667
NM_022193	60581	Acaca	-1.73684	0.876653
NM_022632	360272	Slit2	-1.74002	0.890788
NM_001271381	364952	Nkd1	-1.74985	0.882208
NM_001014762	296278	Pdrg1	-1.75803	0.906997
NM_022225	25075	Htr1b	-1.7626	0.819761
NM_022389	64191	Dhcr7	-1.76641	0.881118
NM_053731	114523	Ntn1	-1.76646	0.89121
NM_053350	84114	Agps	-1.77481	0.894233
NM_001106550	296469	Nkain4	-1.77738	0.826195
NM_001107052	303559	Arl4d	-1.77818	0.843179
NM_199101	308584	Plekha4	-1.78059	0.912799
NM_001001515	361084	Lmo7	-1.78501	0.88706
NM_012848	25319	Fth1	-1.78631	0.914803
NM_053473	84685	Ppp1r9a	-1.7879	0.848207
NM_001108533	361840	Spock2	-1.79256	0.895007
NM_001107269	305956	Wdfy2	-1.80078	0.882525
NM_001276715	85421	Prkd1	-1.80766	0.840647
NM_001047915	362799	Rapgef5	-1.80851	0.831153
NM_001106304	293546	Gprc5b	-1.81023	0.906786
NM_080892	140927	Selenbp1	-1.8139	0.880098
NM_001105879	288093	Arhgap31	-1.81565	0.904501
NM_175578	140666	Rcan2	-1.81726	0.840155
NM_022266	64032	Ctgf	-1.81992	0.889416

NM_001108693	362662	Rbp7	-1.82196	0.840752
NM_198731	290551	Chdh	-1.82198	0.819093
NM_139189	246046	Lmbrd1	-1.82221	0.903903
NM_001108226	316526	Wnt6	-1.82292	0.878446
NM_012828	25297	Cacnb3	-1.8239	0.905942
NM_001013858	287472	Tlcd1	-1.82901	0.856399
NM_001007092	313717	Clstn1	-1.82945	0.912271
NM_053566	89830	Ptch1	-1.83417	0.908298
NM_001034081	313615	Paqr7	-1.83598	0.899191
NM_031521	24586	Ncam1	-1.8364	0.884705
NM_001105983	289338	Disp1	-1.8372	0.89244
NM_001007691	308807	Prss23	-1.83998	0.891667
NM_001002289	432392	Fut8	-1.84353	0.910127
NM_022269	64036	Cd55	-1.84435	0.853833
NM_001163168	25054	Ntrk2	-1.84443	0.886111
NM_031028	81657	Gabbr1	-1.84527	0.912975
NM_001135009	290905	Col4a1	-1.84645	0.915858
NM_017060	24913	Pla2g16	-1.84953	0.914662
NM_001013138	306771	Tspan17	-1.8533	0.910584
NM_080906	140942	Ddit4	-1.8577	0.905626
NM_001108938	365691	Egflam	-1.86074	0.856048
NM_032083	84030	Chn1	-1.86229	0.90436
NM_001007645	297109	Mturn	-1.86342	0.848629
NM_001106840	300850	Gsta4	-1.86421	0.901758
NM_001013231	364052	Pea15	-1.86541	0.917616
NM_206845	298859	Dnajc27	-1.87203	0.837975
NM_031667	60568	Syt11	-1.87433	0.905767
NM_001024334	500300	LOC500300	-1.87593	0.900527
NM_001106276	293056	Cpeb1	-1.87962	0.878868
NM_001025775	501194	Ppip5k2	-1.8832	0.884001
NM_001107681	310506	Ppm1l	-1.88325	0.892405
NM_001013967	303211	Mmgt2	-1.88938	0.895183
NM_013060	25587	Id2	-1.88993	0.899402
NM_001107131	304375	Agfg2	-1.8968	0.862834
NM_001108015	313974	Trib2	-1.89976	0.897257
NM_001172151	498902	Cmtm4	-1.90036	0.870218
NM_001131013	303798	Arvcf	-1.90415	0.9032
NM_017090	497757	Gucy1a3	-1.90659	0.806188
NM_001003929	313173	Cntfr	-1.91263	0.898453
NM_001134698	641316	Aldh4a1	-1.91456	0.846378
NM_001024267	317409	MGC109340	-1.91552	0.885302
NM_001108644	362429	Mfap5	-1.91643	0.915049
NM_031518	24560	Cd200	-1.91779	0.911181
NM_031699	65129	Cldn1	-1.91981	0.91403

NM_001100657	291445	Megf10	-1.92637	0.800492
NM_001082410	500636	Rnf144a	-1.92676	0.840541
NM_001029899	301460	Adam23	-1.92809	0.825563
NM_198761	304135	Adamts5	-1.93111	0.859177
NM_001012163	361303	Lims2	-1.93196	0.916842
NM_001271143	306792	S1pr3	-1.93651	0.905872
NM_017274	29653	Gpam	-1.93793	0.888678
NM_001108084	314751	Tmcc3	-1.95197	0.876828
NM_012868	25339	Npr3	-1.95436	0.855696
NM_001107768	311384	Sema6d	-1.95476	0.897328
NM_021760	60379	Col5a3	-1.96936	0.918671
NM_001007721	360468	Emp2	-1.97359	0.917159
NM_001025112	288611	Wbscr17	-1.97584	0.858052
NM_178105	306439	Gpm6a	-1.97687	0.903833
NM_001191647	287478	Ctns	-1.98312	0.905063
NM_017348	50690	Slc6a8	-1.98839	0.914451
NM_024132	29347	Faah	-1.99046	0.815471
NM_001134604	498392	Cytl1	-1.99275	0.883052
NM_012498	24192	Akr1b1	-2.00042	0.920992
NM_012543	24309	Dbp	-2.00072	0.857595
NM_031321	83467	Slit3	-2.00558	0.890155
NM_012837	25307	Cst3	-2.00797	0.921554
NM_001108415	361251	Elmo1	-2.00922	0.907841
NM_053455	84586	Fgl2	-2.01355	0.921062
NM_031623	58844	Grb14	-2.01462	0.89339
NM_001201369	292077	Sult5a1	-2.01536	0.832771
NM_133651	25404	Cav1	-2.01738	0.860584
NM_053629	114031	Fst13	-2.01776	0.861674
NM_001037218	304299	Radil	-2.02218	0.825176
NM_013180	25724	Itgb4	-2.02235	0.921097
NM_001108653	362466	Bicd1	-2.02376	0.829641
NM_001047102	360687	Cadm2	-2.02449	0.883228
NM_017332	50671	Fasn	-2.02529	0.913045
NM_001104633	246262	Sema3d	-2.03104	0.864768
NM_001107899	312912	Prex2	-2.03306	0.865788
NM_012634	24689	Prps2	-2.03435	0.904079
NM_080882	29213	Tubb4a	-2.04296	0.918143
NM_001001799	308134	Tmem35	-2.046	0.894093
NM_001008520	313917	Abhd1	-2.05582	0.892757
NM_001135878	363517	Plxnb3	-2.05647	0.889768
NM_001128194	688993	Kctd7	-2.05744	0.844515
NM_017094	25235	Ghr	-2.06036	0.881821
NM_001107445	307997	Hspa12a	-2.0701	0.915893
NM_012548	24323	Edn1	-2.0711	0.867792

NM_001114401	282587	Cttnbp2	-2.07233	0.866104
NM_032084	84031	Chn2	-2.07316	0.900985
NM_019328	54278	Nr4a2	-2.07447	0.900985
NM_001109563	689986	LOC689986	-2.07878	0.857103
NM_001106483	295647	Gca	-2.0789	0.900633
NM_001100882	290562	Sema3g	-2.08518	0.877848
NM_001244933	100361818	Nfatc1	-2.08807	0.909283
NM_001011922	291044	Nedd9	-2.09075	0.881153
NM_001106769	299611	Apc2	-2.09147	0.841948
NM_001108000	313774	Fam132a	-2.09326	0.811428
NM_138518	171547	Crispld2	-2.09591	0.917792
NM_001106750	299236	Flrt2	-2.09991	0.858615
NM_001100690	308572	Myh14	-2.10495	0.909669
NM_001130542	299940	Sntb1	-2.10582	0.904993
NM_001109364	503306	Sowahc	-2.10636	0.889662
NM_133396	170908	Tesk2	-2.10957	0.844058
NM_001100901	362242	Snta1	-2.11064	0.913643
NM_172033	64471	Plekhb1	-2.11079	0.924156
NM_001013218	362835	Reep6	-2.11165	0.896871
NM_181386	353229	Sgms1	-2.11286	0.905731
NM_001110143	306454	Cldn22	-2.1137	0.885724
NM_001033883	24772	Cxcl12	-2.11568	0.81512
NM_001007712	316384	Sdpr	-2.11726	0.916842
NM_022608	64543	Sec14l3	-2.12143	0.869831
NM_001109597	690489	Cys1	-2.13287	0.829149
NM_053926	116723	Pip4k2a	-2.14006	0.91621
NM_001108303	360652	Sdk2	-2.14581	0.822011
NM_001006995	308100	Acat2	-2.14989	0.914451
NM_001107869	312492	Dysf	-2.15743	0.850738
NM_017136	29230	Sqle	-2.17376	0.91294
NM_053018	24936	Cd9	-2.1794	0.924719
NM_031031	81660	Gatm	-2.18575	0.92282
NM_001107236	305497	Cobl	-2.19084	0.842475
NM_001276721	85420	Prkcq	-2.20398	0.904712
NM_001108162	315762	Rasl12	-2.20918	0.878903
NM_131914	363425	Cav2	-2.21049	0.920956
NM_017312	29884	Bok	-2.22805	0.902954
NM_031840	83791	Fdps	-2.23366	0.92166
NM_053536	85497	Klf15	-2.23525	0.883228
NM_019230	29504	Slc22a3	-2.23537	0.865788
NM_052809	81718	Cdo1	-2.23741	0.902672
NM_031817	83717	Omd	-2.23926	0.909107
NM_177481	140915	Slco3a1	-2.24201	0.915295
NM_001106892	301261	Enpp4	-2.24805	0.911568

NM_012927	25409	Cdh6	-2.25843	0.8359
NM_133623	171163	Slc6a13	-2.26522	0.898347
NM_013090	25624	Vamp1	-2.27419	0.859423
NM_017353	50719	Slc7a5	-2.27674	0.917616
NM_001134993	499566	Car13	-2.28548	0.902496
NM_031798	83629	Slc12a2	-2.28721	0.920113
NM_001106159	291699	Stard4	-2.28987	0.853481
NM_001109547	689711	Fam101a	-2.30137	0.869163
NM_001171177	299762	Tmtc2	-2.31006	0.830837
NM_031062	81726	Mvd	-2.31216	0.901934
NM_001079888	303384	Mmp28	-2.32055	0.898523
NM_017174	29354	Pla2g5	-2.32416	0.891772
NM_031048	81680	Lifr	-2.32734	0.881505
NM_199502	363455	Chrdl1	-2.33314	0.859634
NM_173151	286936	Pcyt1b	-2.33392	0.806646
NM_001012345	252900	Dgat2	-2.33489	0.915225
NM_001111341	690102	Hist2h2ab	-2.3364	0.920921
NM_053935	116743	Sh3gl2	-2.33656	0.889381
NM_031813	83708	Mybph	-2.34378	0.86417
NM_001014244	365345	Cyb5r2	-2.36077	0.917862
NM_031345	83514	Tsc22d3	-2.36412	0.924824
NM_001107947	313385	Kank4	-2.36716	0.916842
NM_138533	171569	Spon2	-2.37472	0.923699
NM_022297	64157	Ddah1	-2.37722	0.925246
NM_001017457	314438	Degs2	-2.39911	0.836498
NM_031766	83575	Cpz	-2.3996	0.88474
NM_012981	25482	Mras	-2.41196	0.918917
NM_031716	65154	Wisp1	-2.41263	0.899965
NM_012999	25507	Pcsk6	-2.41569	0.893108
NM_138914	192348	Fnbp1	-2.42541	0.922785
NM_031049	81681	Lss	-2.43215	0.907032
NM_023104	65984	Aacs	-2.43556	0.905204
NM_001106610	297453	Hdac11	-2.45314	0.897433
NM_173094	24450	Hmgcs2	-2.45381	0.841456
NM_053375	84390	Hcn1	-2.45515	0.83808
NM_053608	94341	Kcnj13	-2.46338	0.892475
NM_001015024	361686	Osbpl5	-2.46516	0.924754
NM_001107533	308787	Adamts13	-2.46658	0.867722
NM_012505	24212	Atp1a2	-2.46768	0.928059
NM_001077641	24654	Plcb1	-2.46773	0.868565
NM_001013049	289734	Smtn	-2.46908	0.925563
NM_001168527	296115	Secisbp2l	-2.48357	0.931716
NM_021690	59326	Rapgef3	-2.48469	0.882841
NM_031003	81632	Abat	-2.48682	0.889276

NM_001191810	301509	Tns1	-2.49309	0.924156
NM_001105833	287644	Phospho1	-2.49438	0.896449
NM_182738	308965	Chp2	-2.49992	0.909072
NM_001107076	303754	Rab40b	-2.53474	0.840823
NM_019292	54232	Car3	-2.54406	0.922855
NM_001113752	362278	Tmem189	-2.54616	0.932736
NM_022860	64828	B4galnt1	-2.54695	0.867229
NM_001032285	406167	Prrt1	-2.56006	0.92166
NM_001004080	296654	Gsn	-2.56289	0.933333
NM_001108045	314332	Tmem63c	-2.57308	0.875457
NM_012820	25288	Acsl1	-2.57795	0.924156
NM_053503	85265	Ajuba	-2.5785	0.90756
NM_031598	29692	Pla2g2a	-2.58614	0.905345
NM_144737	246245	Fmo2	-2.60005	0.903973
NM_031543	25086	Cyp2e1	-2.61213	0.911146
NM_053681	114216	S100a3	-2.63514	0.875949
NM_053633	114090	Egr2	-2.65082	0.927039
NM_013198	25750	Maob	-2.65215	0.929677
NM_022533	64364	Pllp	-2.65655	0.935724
NM_017171	29340	Prkce	-2.66664	0.87609
NM_001108233	316639	Farp2	-2.67236	0.904887
NM_021595	59115	Ninj2	-2.67468	0.861568
NM_012671	24827	Tgfa	-2.68256	0.921484
NM_019363	54349	Aox1	-2.68729	0.90225
NM_017137	29232	Clcn2	-2.68901	0.920816
NM_001009399	309262	Nsdhl	-2.69965	0.929923
NM_022499	25269	Pvalb	-2.70735	0.800844
NM_133567	171097	Adap1	-2.72118	0.89891
NM_022008	63848	Fxyd7	-2.72173	0.925598
NM_145717	29639	Fxyd2	-2.73535	0.903129
NM_031834	83783	Sult1a1	-2.73649	0.933509
NM_053535	85496	Enpp1	-2.74436	0.903973
NM_001135007	680451	Nrbp2	-2.75243	0.935197
NM_012946	25434	Sparcl1	-2.75357	0.936709
NM_001107495	308445	Cyp2s1	-2.75807	0.932208
NM_053777	116457	Mapk8ip1	-2.78056	0.937236
NM_213626	305967	Kif13b	-2.78818	0.923945
NM_001107793	311569	Acss2	-2.78943	0.924965
NM_001108374	361032	RGD1310110	-2.79072	0.905591
NM_024399	79251	Aspa	-2.79087	0.930661
NM_001011991	299923	Ndrg1	-2.79559	0.938748
NM_001024276	366792	Mettl7b	-2.80227	0.89782
NM_001107023	303337	Rab11fip4	-2.80498	0.818038
NM_001191800	316023	Dclk3	-2.80933	0.898031

NM_031688	64347	Sncg	-2.81038	0.938783
NM_053715	114507	Slc5a3	-2.81545	0.853586
NM_001106893	301264	Cyp39a1	-2.8314	0.851477
NM_001106003	289594	Nipal1	-2.84299	0.814944
NM_031556	25404	Cav1	-2.85051	0.878551
NM_019276	50555	Ugt8	-2.86488	0.940084
NM_030852	81510	Mia	-2.87084	0.868636
NM_001025688	310811	Palmd	-2.8789	0.927637
NM_001109263	500527	Lurap1	-2.92214	0.922433
NM_031715	65152	Pfkm	-2.9232	0.934423
NM_133296	113918	Slc6a20	-2.92443	0.83917
NM_182844	360034	Myrip	-2.93865	0.814557
NM_053346	83834	Nrn1	-2.94295	0.893636
NM_001108669	362540	Ptplad2	-2.94389	0.928446
NM_144744	246253	Adipoq	-2.94662	0.919831
NM_001127567	500841	RGD1561648	-2.94844	0.80218
NM_001100974	307398	Arhgef37	-2.95017	0.840788
NM_019361	54323	Arc	-2.97533	0.867792
NM_053642	114100	Sc5d	-2.97991	0.937693
NM_001010958	314441	Slc25a29	-2.98155	0.893882
NM_017308	29744	Sema6c	-2.98183	0.933826
NM_001107942	313325	Lad1	-2.98237	0.911779
NM_017070	24950	Srd5a1	-3.01372	0.931259
NM_053433	84493	Fmo3	-3.01764	0.809494
NM_080886	140910	Msmo1	-3.0278	0.94244
NM_001108578	362107	Aifl1	-3.03567	0.937447
NM_012497	24191	Aldoc	-3.03588	0.930907
NM_001108438	361364	Dok4	-3.04292	0.925598
NM_053505	85267	Slc24a3	-3.04581	0.816561
NM_134459	171485	Cd9912	-3.0645	0.938502
NM_001106081	678743	Tll1	-3.07067	0.853762
NM_012881	25353	Spp1	-3.07772	0.945886
NM_001037200	313840	Rmdn2	-3.08279	0.905802
NM_001025129	361018	Fam107a	-3.08554	0.934951
NM_138502	29254	Mgll	-3.10096	0.944444
NM_012941	25427	Cyp51	-3.10559	0.944691
NM_001106104	291022	Ptpdc1	-3.11583	0.932384
NM_001048042	289095	Nmnat2	-3.13017	0.85756
NM_012517	24239	Cacna1c	-3.14029	0.904747
NM_175762	300438	Ldlr	-3.1725	0.93161
NM_001106305	293561	Rnf152	-3.18311	0.828903
NM_001107239	305509	Adcy1	-3.18742	0.89884
NM_001191563	309533	Sorcs1	-3.18983	0.940331
NM_013134	25675	Hmgcr	-3.19557	0.941526

NM_001109426	680723	Tmem88b	-3.21415	0.91962
NM_012598	24539	Lpl	-3.22407	0.941983
NM_182954	360202	Ston1	-3.23204	0.940858
NM_001130502	315145	Fam83f	-3.25063	0.925703
NM_001106985	302975	Syngr3	-3.26836	0.827567
NM_017255	29597	P2ry2	-3.26949	0.941174
NM_001037336	641521	Lrrc4	-3.28024	0.877391
NM_134376	171393	Clstn3	-3.28476	0.900985
NM_012945	25433	Hbegf	-3.28562	0.94462
NM_001109423	680615	Hist1h2ak	-3.29671	0.807068
NM_001107857	312275	Ephb6	-3.30467	0.918319
NM_199370	25626	Krt8	-3.31839	0.910197
NM_013107	25644	Bmp6	-3.34806	0.887904
NM_001107591	309377	Sfrp5	-3.35541	0.952532
NM_001107811	311730	Gpr155	-3.36314	0.920675
NM_133295	113902	Ces1d	-3.38735	0.832349
NM_031154	81869	Gstm7	-3.45042	0.945851
NM_153311	266681	Tmprss5	-3.46989	0.940928
NM_001170597	362931	Bai1	-3.4724	0.819726
NM_001108584	362161	Rapsn	-3.4758	0.896835
NM_001107056	303601	Cyb561	-3.50425	0.925316
NM_001107065	303678	Caskin2	-3.50869	0.950422
NM_001100713	313644	Rap1gap	-3.5432	0.940366
NM_139192	246074	Scd1	-3.5514	0.945113
NM_198780	362282	Pck1	-3.55348	0.892229
NM_001080148	298298	Dhcr24	-3.55904	0.95443
NM_022392	64194	Insig1	-3.56726	0.950316
NM_001100842	305104	Col9a1	-3.57029	0.927954
NM_001107583	309312	Gldc	-3.57186	0.913221
NM_001130548	314981	Col14a1	-3.57598	0.950387
NM_001134610	499126	Igflr1	-3.61419	0.865928
NM_053457	84588	Cldn11	-3.61695	0.878692
NM_053767	114767	Ptpre	-3.63469	0.947257
NM_012935	25420	Cryab	-3.64349	0.960267
NM_138521	360616	Ppp1r1b	-3.66352	0.942546
NM_012808	25274	Tst	-3.67024	0.934177
NM_012703	25357	Thrsp	-3.68871	0.93474
NM_031066	81730	Fez1	-3.68978	0.95334
NM_130431	161476	Hspb2	-3.70088	0.951195
NM_017155	29290	Adora1	-3.70533	0.892686
NM_017251	29584	Gjb1	-3.71671	0.957771
NM_001104527	312358	Prr15	-3.72294	0.918003
NM_053621	113970	Magi2	-3.72732	0.927707
NM_012676	24837	Tnnt2	-3.75201	0.94135

NM_001165880	406166	Egfl8	-3.75962	0.957489
NM_001191678	305332	Limch1	-3.7762	0.960091
NM_001191072	680616	Ppp1r16b	-3.78475	0.940823
NM_001106662	298141	Frmd3	-3.79467	0.931329
NM_001108526	361764	Sema4g	-3.80388	0.951371
NM_001105749	116996	Il16	-3.81903	0.945534
NM_001108970	366352	Mob3b	-3.858	0.955802
NM_057116	117256	Ppp2r2c	-3.86427	0.948734
NM_001107713	310764	Fam212b	-3.86522	0.958368
NM_001107659	310207	Sema5a	-3.86938	0.963221
NM_001108088	314772	Epyc	-3.89296	0.891491
NM_022215	60666	Gpd1	-3.90526	0.959283
NM_001034944	366962	Grap2	-3.92902	0.820394
NM_001134746	500219	Lrrtm4	-3.96812	0.85654
NM_001106784	299802	Lgr5	-3.97596	0.940471
NM_001135834	288271	Mrap	-4.04257	0.959494
NM_001107645	309922	Rhobtb3	-4.06667	0.962869
NM_001012049	306424	Mfap3l	-4.11146	0.962904
NM_022182	29348	Fgf7	-4.1455	0.964276
NM_153737	266803	Sostdc1	-4.15046	0.969304
NM_001191077	681021	Paqr6	-4.16694	0.969972
NM_019238	29580	Fdft1	-4.20586	0.966491
NM_017027	24564	Mpz	-4.21414	0.973031
NM_001109394	680178	Cldn20	-4.21641	0.903622
NM_053539	89784	Idi1	-4.23582	0.968847
NM_001105754	192247	Sez6	-4.26629	0.871308
NM_017184	29388	Tnni1	-4.29093	0.916842
NM_001271084	499157	Svip	-4.29956	0.973418
NM_001013071	293688	Tm7sf2	-4.32916	0.947398
NM_019169	29219	Snca	-4.33251	0.97398
NM_017235	29540	Hsd17b7	-4.33394	0.899719
NM_199498	360626	Krt19	-4.3407	0.961814
NM_001109467	685462	Emid1	-4.35678	0.964944
NM_134383	171402	Elovl6	-4.36121	0.963045
NM_183403	29326	Gpx2	-4.36123	0.970921
NM_017268	29637	Hmgcs1	-4.477	0.978165
NM_012561	24373	Fst	-4.48177	0.886463
NM_031686	64155	Scn7a	-4.51531	0.977215
NM_001271202	303659	Kif19	-4.52187	0.972257
NM_001163921	685202	Efcc1	-4.53597	0.95545
NM_001012009	303272	Pipox	-4.54771	0.801653
NM_031069	81733	Nell1	-4.55726	0.923383
NM_019278	50561	Resp18	-4.59118	0.956188
NM_053955	117024	Crym	-4.60931	0.813221

NM_212522	314251	Sptb	-4.62808	0.966421
NM_145670	246755	Bcas1	-4.64421	0.976758
NM_001108901	365038	Samd5	-4.65913	0.839979
NM_001107745	311184	C1qtnf4	-4.6721	0.851266
NM_012721	25041	P2rx6	-4.7306	0.834318
NM_144741	246250	Retn	-4.78619	0.912693
NM_031743	84550	Slc24a2	-4.82525	0.854923
NM_012686	24877	Vsnl1	-4.8437	0.948383
NM_012972	25470	Kcna5	-4.85883	0.96839
NM_173152	286937	Pex5l	-4.89426	0.920781
NM_153294	259224	Npw	-4.92268	0.950141
NM_001127538	307925	Spire2	-4.92578	0.947046
NM_021687	59323	Erbb4	-4.99523	0.861006
NM_053504	85266	Prss12	-4.99643	0.976758
NM_080692	140725	Cacng4	-5.00347	0.951231
NM_001191069	680317	Endou	-5.01027	0.8032
NM_001191575	116680	Ptpru	-5.03579	0.915436
NM_001047103	360882	Cadm3	-5.05788	0.98692
NM_001104528	24338	Ephb1	-5.08799	0.821624
NM_001109403	680404	C1ql3	-5.16303	0.980591
NM_213624	291325	St8sia6	-5.16704	0.878586
NM_001105994	289440	Ephx4	-5.22967	0.948136
NM_001002835	314280	Smoc1	-5.28809	0.978235
NM_001109480	685756	Tmem229a	-5.31	0.971484
NM_021688	59324	Kcnk1	-5.334	0.981083
NM_001191844	361895	Elovl7	-5.39693	0.981821
NM_030856	81514	Lrrn3	-5.42194	0.987342
NM_012970	25468	Kcna2	-5.42647	0.973558
NM_017037	24660	Pmp22	-5.4316	0.991948
NM_138530	171564	Pbld1	-5.43763	0.949543
NM_001109492	686081	B3galt2	-5.44773	0.846695
NM_017226	29511	Padi2	-5.48407	0.984775
NM_001109514	688790	Pmp2	-5.50611	0.992335
NM_001127504	499156	Gas2	-5.51729	0.965506
NM_057156	117523	St8sia2	-5.59848	0.880309
NM_001108491	361602	Me3	-5.60689	0.89666
NM_001170399	304280	Tmem130	-5.64408	0.904817
NM_019375	56003	3-Sep	-5.7004	0.983931
NM_013015	25526	Ptgds	-5.71385	0.924262
NM_024346	29246	Stmn3	-5.71851	0.925105
NM_031334	83502	Cdh1	-5.84581	0.990471
NM_013166	25707	Cntf	-5.87297	0.994163
NM_001008514	298487	Cldn19	-5.87387	0.994304
NM_001135253	689933	Syce3	-5.96363	0.886568

NM_001004022	287700	Krt15	-6.0528	0.913959
NM_019183	29275	Actc1	-6.14935	0.816245
NM_053968	117038	Mt3	-6.15968	0.994761
NM_012798	25263	Mal	-6.25067	0.996062
NM_001134845	688613	LOC688613	-6.25139	0.972961
NM_012846	25317	Fgf1	-6.27299	0.990788
NM_001106110	291130	Dcdc2	-6.28988	0.855098
NM_001135583	307855	Fa2h	-6.2988	0.994972
NM_145769	252892	Lgi1	-6.35194	0.965928
NM_181382	315675	Gldn	-6.369	0.993952
NM_001047870	300242	Krt7	-6.38493	0.946273
NM_017190	29409	Mag	-6.47081	0.991772
NM_001013943	299052	Aldoart2	-6.53066	0.831575
NM_001109374	679668	LRRTM1	-6.59159	0.849402
NM_022285	64057	Hapln2	-6.60726	0.984072
NM_012654	24784	Slc9a3	-6.67636	0.9391
NM_017069	24947	Gabra3	-6.76037	0.959916
NM_023971	66027	Drp2	-6.8163	0.99666
NM_001012738	29593	Ckmt1b	-6.92145	0.877672
NM_023976	78960	Prx	-6.92751	0.997433
NM_001253918	689826	Ncmap	-6.96134	0.997398
NM_001005898	450224	Omg	-6.97605	0.964803
NM_213628	364901	St8sia5	-7.03911	0.972996
NM_139339	246235	Slc36a2	-7.07608	0.992686
NM_053440	84510	Stmn2	-7.08063	0.874297
NM_173095	24520	Kcna1	-7.09536	0.994655
NM_012929	25412	Col2a1	-7.26458	0.991737
NM_030854	81512	Lect1	-7.35998	0.996484
NM_001169141	306616	Myom2	-7.6294	0.987412
NM_022866	64846	Slc13a3	-7.66229	0.98045
NM_181636	353303	Col23a1	-7.7008	0.99128
NM_001135710	287060	Sec14l5	-7.97015	0.926828
NM_144756	246274	Faim2	-8.2435	0.991632
NM_181433	305427	Otop1	-8.41374	0.956857
NM_001008809	406227	Krt72	-8.73526	0.815155
NM_001105894	288289	Chodl	-8.7941	0.822082
NM_001134580	362133	Lypd6b	-8.94417	0.837799
NM_001008311	295619	Ermn	-9.10031	0.852989
NM_030993	25113	Ddn	-9.11079	0.981716
NM_053514	85327	Lin7a	-9.64074	0.900492
NM_053809	116499	Fgf4	-10.636	0.95225

In this table up-regulated genes are highlighted in red and the down-regulated genes are highlighted in green.

Table S10. DEGs between normal sensory nerves and injured sensory nerves

RNA_nucleotide_accession	GeneID	GeneSymbol	log2Ratio	Probability
NM_133286	29349	Fgf8	9.550824	0.893317
NM_001100964	296935	Lmod2	8.992652	0.840861
NM_145083	246296	Lrrc15	8.614318	0.981205
NM_001106545	296395	Kcng1	8.438537	0.982408
NM_080411	140595	Gpr83	8.406094	0.99016
NM_032070	84017	Hmga2	8.133376	0.975117
NM_031089	81753	Pth2r	7.805264	0.95841
NM_001107189	304913	Tnn	7.702414	0.800687
NM_017091	25204	Pcsk1	7.681731	0.994195
NM_022211	60662	Fgf5	7.293423	0.995611
NM_001106579	296789	Sema3e	7.05661	0.976179
NM_001106858	301012	Col7a1	6.591819	0.967153
NM_019139	25453	Gdnf	6.586586	0.964675
NM_053881	116660	Ptprn	6.428169	0.985559
NM_001105734	63995	Dusp10	6.212532	0.993275
NM_001106671	298300	Ttc22	6.167632	0.909776
NM_201420	362280	Tfap2c	6.042939	0.858983
NM_012610	24596	Ngfr	6.011665	0.994868
NM_031091	81755	Rab3b	5.95469	0.989027
NM_001108111	315294	Wnt10b	5.952091	0.945774
NM_133385	170896	Ucn2	5.859839	0.982621
NM_001107504	308511	Chst8	5.848351	0.925952
NM_001107280	306081	Pcdh20	5.833044	0.971329
NM_019344	54297	Rgs8	5.755608	0.920607
NM_053763	114700	Cyp27b1	5.676246	0.875938
NM_130407	154516	Ugt1a7c	5.640948	0.977418
NM_001044269	500750	Tmem196	5.600616	0.937845
NM_001107345	306862	Tfap2a	5.550259	0.852789
NM_001106134	291441	Ska1	5.45811	0.835268
NM_001109536	689388	Ptx3	5.445497	0.882699
NM_022196	60584	Lif	5.434834	0.95926
NM_012690	24891	Abcb4	5.376434	0.971896
NM_001271181	100361733	Ociad2	5.358358	0.952994
NM_001013894	292594	Lilrb4	5.167773	0.910024
NM_017244	29563	Crabp2	5.097683	0.986125
NM_024127	25112	Gadd45a	4.845884	0.983258
NM_001109102	498533	Fam167a	4.833055	0.861674
NM_012932	25415	Crmp1	4.832514	0.883973
NM_013077	25609	Tub	4.797936	0.889955
NM_001106536	296344	Mybl2	4.692099	0.801749
NM_031689	64348	Cryba4	4.651819	0.918944

NM_001106629	297738	Steap1	4.614194	0.884433
NM_022256	64022	Btc	4.580368	0.972639
NM_199082	287884	Sectm1b	4.526547	0.919475
NM_001079937	290326	Pbk	4.399917	0.910944
NM_001113781	360559	Fam64a	4.380026	0.933775
NM_012752	25145	Cd24	4.374441	0.966763
NM_012488	24153	A2m	4.354152	0.975258
NM_001106991	303004	Tmem8a	4.336216	0.960817
NM_053896	116676	Aldh1a2	4.32047	0.955932
NM_031517	24553	Met	4.294398	0.950446
NM_022274	64041	Birc5	4.257669	0.895052
NM_001077650	366270	Edn3	4.250954	0.94811
NM_030828	58920	Gpc1	4.249606	0.973701
NM_001025740	362720	Rrm2	4.232171	0.961206
NM_053719	114511	Emb	4.215471	0.935651
NM_001014095	315952	Dzip1l	4.214374	0.948959
NM_001013886	291081	Tubb2b	4.178996	0.970126
NM_001100827	257649	Cenpf	4.155491	0.842489
NM_001271362	294297	Scube3	4.153936	0.864895
NM_001107702	310694	Fcrl2	4.139913	0.947331
NM_201418	300795	Ns5atp9	4.139395	0.909422
NM_001130500	300126	Gtse1	4.129903	0.890733
NM_172333	282836	Cthrc1	4.123262	0.826986
NM_001109419	680551	Apoc4	4.106132	0.878345
NM_001039344	500040	Tes	4.102647	0.965914
NM_022183	360243	Top2a	4.075713	0.932359
NM_001134969	500430	Ankrd6	4.072334	0.926695
NM_022624	64559	Slc22a23	4.013561	0.966657
NM_001108155	315740	Kif23	3.995748	0.898308
NM_138541	171577	Epcam	3.974233	0.888751
NM_012732	25055	Lipa	3.962071	0.964569
NM_001007648	297594	Cdca3	3.95276	0.925952
NM_001008882	316129	Uhrf1	3.899376	0.907511
NM_001191763	298506	Mycl	3.894265	0.928076
NM_001108172	315852	Ttk	3.87349	0.828118
NM_139089	245920	Cxcl10	3.846874	0.962516
NM_171991	25203	Ccnb1	3.790137	0.885778
NM_001107135	304388	Cldn15	3.789987	0.834985
NM_052800	24834	Tk1	3.780635	0.93296
NM_019296	54237	Cdk1	3.735748	0.934659
NM_001077589	360857	Rgs16	3.733411	0.960675
NM_001004264	311325	Knstrn	3.713659	0.884893
NM_001009654	295661	Spc25	3.709765	0.877991
NM_001106542	296368	Ube2c	3.709668	0.940287

NM_053744	114587	Dlk1	3.70867	0.930058
NM_053397	362572	Artn	3.676871	0.908537
NM_001025646	294074	Cep55	3.671995	0.8484
NM_001107946	313370	Hook1	3.66931	0.868823
NM_001108495	361615	Relt	3.656951	0.834242
NM_001108223	316507	Shd	3.646194	0.910201
NM_144762	140720	Baalc	3.643148	0.948181
NM_139327	117062	Hmga1	3.625586	0.959366
NM_022617	64552	Mpeg1	3.618105	0.952074
NM_001205348	501736	Cd300a	3.617782	0.87569
NM_053702	114494	Ccna2	3.603741	0.921138
NM_001191709	310621	Iqgap3	3.592414	0.897246
NM_001025768	499933	Dsn1	3.589325	0.87346
NM_012959	25454	Gfra1	3.569637	0.95972
NM_001107160	304648	Asf1b	3.532041	0.825428
NM_001107807	311676	Pmepa1	3.531221	0.956888
NM_001009470	363088	Ccnb2	3.523314	0.915086
NM_001100474	117524	Ccnf	3.51595	0.840153
NM_001107529	308761	Prc1	3.49313	0.928678
NM_017100	25515	Plk1	3.458108	0.865001
NM_001107846	312052	Steap2	3.45359	0.901069
NM_001108547	361921	Ect2	3.450698	0.819588
NM_012912	25389	Atf3	3.445301	0.955614
NM_053861	116640	Tnc	3.443299	0.948181
NM_019333	54283	Pfkfb4	3.441851	0.932359
NM_001004424	360962	Tacc3	3.441485	0.899051
NM_001108633	362384	Reep1	3.430883	0.888574
NM_133393	170905	Lfng	3.414474	0.949101
NM_001009353	301265	Pla2g7	3.413681	0.803695
NM_001031638	287435	Cd68	3.400137	0.948074
NM_139097	245956	Scn3b	3.387759	0.932323
NM_022391	64193	Pttg1	3.354318	0.904786
NM_001011930	292206	Trip13	3.353219	0.805147
NM_133298	113955	Gpnmb	3.345664	0.95211
NM_001126083	498709	Cks2	3.316239	0.908219
NM_001106192	292071	Cdt1	3.299489	0.858488
NM_001106170	291885	Mcm5	3.296479	0.879938
NM_207615	365395	Clcf1	3.295627	0.925988
NM_001107762	311336	Nusap1	3.294923	0.899193
NM_001169112	171304	Kif11	3.284511	0.854913
NM_001107369	307169	Mastl	3.276195	0.805111
NM_022300	64160	Basp1	3.275991	0.937916
NM_001013433	364396	Arl11	3.274501	0.90075
NM_001135016	308602	Tmem86a	3.262659	0.913882

NM_012980	25481	Mmp11	3.257611	0.912927
NM_001271366	291234	Mki67	3.250492	0.912077
NM_001191862	362332	Flnc	3.227032	0.940925
NM_001100684	305450	Sh3bp2	3.200726	0.918377
NM_001015032	366061	Galnt3	3.192367	0.888645
NM_001107777	311426	Siglec1	3.181655	0.889247
NM_013185	25734	Hck	3.162497	0.893176
NM_001109181	499593	Sox2	3.157443	0.944783
NM_013113	25650	Atp1b1	3.151035	0.917209
NM_001169139	306575	Ckap2	3.145302	0.886698
NM_001109418	680531	Sapcd2	3.133671	0.814774
NM_001108287	360591	Prr11	3.130999	0.851409
NM_001107512	308582	Tead2	3.127599	0.875867
NM_001271243	300219	Troap	3.124455	0.810881
NM_012953	25445	Fosl1	3.113621	0.911298
NM_001107159	304608	Mmp19	3.107366	0.941385
NM_001106507	296137	Bub1	3.095062	0.841852
NM_022634	64569	Lst1	3.089487	0.876504
NM_001173386	502902	Clec7a	3.085227	0.901104
NM_030859	81517	Mdk	3.056975	0.939579
NM_053734	114553	Ncf1	3.052978	0.919935
NM_001105925	288626	Mmp17	3.049558	0.916926
NM_001013927	297694	Plbd1	3.045846	0.875478
NM_001039019	303575	Kif18b	3.034831	0.826915
NM_057212	117582	Tmem158	3.02444	0.940075
NM_001025050	500545	Cdca8	3.00389	0.875903
NM_001106884	301227	Trem2	2.998696	0.916466
NM_053749	114592	Aurkb	2.987051	0.862381
NM_053698	114490	Cited2	2.986538	0.937456
NM_182824	315716	Cd276	2.979875	0.940075
NM_001126270	301701	Ndc80	2.974854	0.863833
NM_001168524	296060	Arhgap11a	2.96919	0.867797
NM_001024277	366959	Gcat	2.965875	0.919864
NM_001191887	363220	Aff3	2.942265	0.894875
NM_001108862	364190	Rasl10a	2.937435	0.92758
NM_001108214	316351	Npas2	2.929849	0.892291
NM_001012028	304951	Nuf2	2.913503	0.866027
NM_181086	302965	Tnfrsf12a	2.884388	0.935049
NM_178095	313210	Abca1	2.881353	0.932465
NM_001191622	308431	Phldb3	2.867232	0.894945
NM_001108112	315298	Racgap1	2.866007	0.883725
NM_031078	81742	Pde1c	2.859378	0.896114
NM_001192006	501872	Cd84	2.827046	0.840507
NM_001191813	316602	Hjurp	2.814002	0.813642

NM_031530	24770	Ccl2	2.809416	0.932642
NM_001107790	311546	Tpx2	2.797818	0.899264
NM_138529	171563	Nav2	2.797796	0.920147
NM_001105769	287110	Tbc1d24	2.793857	0.900857
NM_001005878	294286	Kifc1	2.793361	0.83049
NM_001106996	303073	Cyfip2	2.785476	0.926412
NM_001191939	498638	Msr1	2.78372	0.844931
NM_023959	66015	Adamts4	2.77775	0.928501
NM_001168543	296371	Pltp	2.775663	0.934695
NM_001012125	315714	Loxl1	2.77119	0.926165
NM_172067	64456	Spon1	2.762753	0.874628
NM_022194	60582	Il1rn	2.760008	0.801182
NM_001170487	293736	Myrf	2.751733	0.917776
NM_001271205	364712	Sox4	2.738701	0.932996
NM_012964	25460	Hmmr	2.734173	0.809536
NM_001135802	289997	Dlgap5	2.729827	0.851373
NM_001107315	306506	Pragmin	2.722963	0.906909
NM_053372	84386	Slpi	2.722504	0.876717
NM_017287	29685	Mcm6	2.720426	0.886911
NM_001130583	689069	Parvg	2.694904	0.823623
NM_012867	25338	Ninj1	2.684025	0.932076
NM_053332	80848	Cubn	2.679424	0.868363
NM_171993	64515	Cdc20	2.67566	0.886911
NM_053489	85251	Col18a1	2.669833	0.932819
NM_001106028	289993	Cdkn3	2.667795	0.80143
NM_031633	58921	Foxm1	2.663403	0.860612
NM_001025767	499356	Blnk	2.644795	0.849604
NM_019354	54315	Ucp2	2.639432	0.931934
NM_012940	25426	Cyp1b1	2.63178	0.88861
NM_001039028	316333	Actr1b	2.626215	0.930554
NM_001107225	305438	Sorcs2	2.623367	0.927403
NM_013031	25549	Slc18a2	2.62112	0.899901
NM_001102408	304944	Uck2	2.613562	0.880787
NM_017203	29454	Gdf11	2.601084	0.91728
NM_001107417	307757	Neto2	2.596798	0.800616
NM_001106074	290655	Crlf1	2.579247	0.928642
NM_053905	116685	Lmnb1	2.578408	0.876752
NM_001009645	293502	Kif22	2.576703	0.88323
NM_001108675	362584	Col9a2	2.576603	0.890202
NM_001037780	309684	Itgb2	2.573513	0.875513
NM_001191882	363126	Nphp3	2.570476	0.838242
NM_001105928	288659	Vps37b	2.56892	0.918342
NM_001169133	306451	Tenm3	2.558167	0.924005
NM_012907	25383	Apobec1	2.557386	0.88861

NM_001014205	363013	Tmem123	2.556827	0.927191
NM_001191950	499254	Tmc7	2.551874	0.901246
NM_001108965	366262	Rbm38	2.549475	0.904715
NM_022618	64553	Akap6	2.544985	0.834667
NM_001271151	688611	Cetn4	2.538192	0.854347
NM_019358	54320	Pdpn	2.528446	0.922271
NM_001107297	306279	Arhgap22	2.505279	0.895335
NM_031832	83781	Lgals3	2.505233	0.929173
NM_019165	29197	Il18	2.498643	0.829959
NM_001191636	309499	Myof	2.498332	0.923368
NM_001006975	293749	Ms4a6bl	2.48965	0.885566
NM_001008515	298566	C1qa	2.476228	0.926058
NM_001190999	679812	Plekhg1	2.465908	0.907688
NM_001109478	685679	Nme4	2.464362	0.844896
NM_019262	29687	C1qb	2.457759	0.923262
NM_031821	83722	Plk2	2.45711	0.921988
NM_001012061	308113	Cnksr3	2.450322	0.914696
NM_053598	94267	Nudt4	2.449621	0.926094
NM_013026	25216	Sdc1	2.440764	0.835374
NM_171992	58919	Ccnd1	2.438121	0.926695
NM_053843	116591	Fcgr2a	2.434887	0.825145
NM_001107749	311209	Tp53i11	2.425459	0.920926
NM_001271189	100360582	Nt5c	2.419523	0.888185
NM_013151	25692	Plat	2.414877	0.925492
NM_001108726	362800	Itgb8	2.413816	0.925244
NM_001168285	498022	RGD1559482	2.39904	0.868717
NM_001009646	293504	Qprt	2.394567	0.914236
NM_001135992	498276	LOC498276	2.392085	0.895122
NM_001191991	500915	Fam19a5	2.391152	0.921634
NM_001126291	366205	Flrt3	2.384012	0.91682
NM_001024262	312727	Tspan11	2.379536	0.911829
NM_001107100	304021	Col8a1	2.377362	0.917245
NM_001106577	296762	Phtf2	2.366579	0.9016
NM_031325	83472	Ugdh	2.366525	0.919333
NM_130409	155012	Cfh	2.356248	0.903228
NM_001013214	362556	Ttc4	2.353026	0.884362
NM_012777	25239	Apod	2.349754	0.925669
NM_001108385	361069	Dmtn	2.342062	0.890556
NM_001037492	641603	Slc41a3	2.327199	0.818491
NM_012843	25314	Emp1	2.326928	0.923616
NM_001107636	309837	Adamts14	2.326785	0.855338
NM_001145828	289337	Tlr5	2.32321	0.843976
NM_001107796	311598	Fam83d	2.322028	0.820367
NM_001107111	304091	Il10rb	2.318112	0.913705

NM_001007679	305070	Tmem206	2.313515	0.902272
NM_021578	59086	Tgfb1	2.294182	0.919298
NM_001107977	313575	Heyl	2.289872	0.87208
NM_001047084	289076	Rgs18	2.288656	0.860328
NM_001014268	367113	Lrrc1	2.282995	0.858134
NM_013080	25613	Ptprz1	2.281126	0.821216
NM_012567	24392	Gja1	2.27784	0.914732
NM_023965	66021	Cybb	2.277672	0.86808
NM_001107464	308212	Dact2	2.273944	0.821429
NM_001047853	293019	Arrdc4	2.266856	0.86624
NM_001191805	316273	Mcm3	2.262163	0.855798
NM_001013149	308795	Mesdc1	2.233573	0.910874
NM_001191792	315579	Ubash3b	2.231245	0.861603
NM_001005539	294422	Smpdl3a	2.227733	0.903122
NM_022205	60628	Cxcr4	2.212804	0.815871
NM_001109584	690366	Vangl1	2.212407	0.903511
NM_021584	83825	Dclk1	2.209039	0.917386
NM_012987	25491	Nes	2.198384	0.919829
NM_001106422	294917	Tnik	2.186116	0.842489
NM_001100802	502317	Aplp1	2.183481	0.875407
NM_021576	58813	Nt5e	2.183233	0.862806
NM_001012127	315994	Mapkapk3	2.18227	0.870204
NM_013025	25542	Ccl3	2.181719	0.80012
NM_019289	54227	Arpc1b	2.173536	0.919262
NM_001134414	290501	Tmtc4	2.172548	0.878663
NM_019197	29366	Serpine2	2.164743	0.919333
NM_001025046	500102	Fam131b	2.143073	0.815022
NM_001191791	315496	Dpy19l1	2.135745	0.910697
NM_019272	29745	Sema4f	2.135425	0.874947
NM_001106710	298845	Emilin1	2.131018	0.878239
NM_001013219	362924	St3gal1	2.129999	0.898733
NM_001039609	310839	Fnbp1l	2.125738	0.881212
NM_017320	50654	Ctss	2.121656	0.909529
NM_001106314	293618	Ifitm1	2.121199	0.917068
NM_022396	64199	Gng11	2.115507	0.911546
NM_001008722	292060	Irf8	2.102356	0.807164
NM_023969	66025	Lpar3	2.098465	0.886804
NM_019143	25661	Fn1	2.088223	0.916643
NM_022217	60668	Amph	2.080654	0.859939
NM_001108210	316325	Hs6st1	2.079623	0.907759
NM_012580	24451	Hmox1	2.077906	0.869567
NM_001191626	308790	Mex3b	2.075748	0.811305
NM_001108065	314612	Shc2	2.069447	0.86401
NM_138850	192203	Fap	2.068499	0.886097

NM_001191680	305449	Mfsd10	2.066524	0.88776
NM_001008278	287278	Cdkn2aipnl	2.058992	0.903051
NM_173101	25484	Myo1e	2.054779	0.909033
NM_001005881	300443	Lppr2	2.05348	0.871018
NM_001106268	292999	Chsy1	2.048057	0.885884
NM_001108664	362516	Msantd3	2.036266	0.874416
NM_152937	266610	Fadd	2.034402	0.811695
NM_203337	363040	St3gal4	2.031405	0.909068
NM_001013158	310508	B3galnt1	2.029714	0.884504
NM_019336	54289	Rgs1	2.024057	0.841675
NM_012523	24251	Cd53	2.013083	0.883902
NM_001106283	293154	Folr2	2.007638	0.872752
NM_001106461	295342	Rhoc	2.006819	0.911405
NM_033443	25227	Arsb	2.00636	0.906237
NM_001006956	288449	Katnal1	1.998253	0.86447
NM_053417	84477	Gab2	1.99809	0.891795
NM_001109300	501065	Cmtm7	1.997166	0.899618
NM_001007554	362650	Fblim1	1.996205	0.834879
NM_012954	25446	Fosl2	1.988484	0.869036
NM_001008556	306808	Ippk	1.984754	0.813111
NM_021835	24516	Jun	1.978493	0.911688
NM_001191558	307845	Mtss1l	1.978459	0.911192
NM_001002807	406864	Clic1	1.978295	0.911192
NM_001012106	313702	Mad2l2	1.977193	0.877
NM_001008321	299626	Gadd45b	1.968954	0.902874
NM_001108587	362187	Ccdc34	1.968183	0.813288
NM_001131001	25441	Fcer1g	1.9674	0.909175
NM_031344	83512	Fads2	1.966353	0.910803
NM_031348	83517	Fcna	1.962313	0.824862
NM_022242	63912	Fam129a	1.954849	0.907192
NM_017074	24962	Cth	1.945807	0.834313
NM_031118	81782	Soat1	1.93491	0.900432
NM_031126	81804	Stxbp2	1.934216	0.818526
NM_031807	83684	Tpbg	1.93417	0.802386
NM_001109648	691750	Tmem107	1.933172	0.80904
NM_001169138	292406	Thbs2	1.928732	0.897529
NM_001202463	360655	Cd300le	1.927384	0.870522
NM_012934	25418	Dpysl3	1.91935	0.883442
NM_021587	59107	Ltbp1	1.916719	0.887725
NM_031826	689008	Fbn2	1.914491	0.822667
NM_001107873	312538	Mcm2	1.911181	0.860293
NM_001100735	361467	Lpcat1	1.907697	0.893565
NM_001107153	304579	Usp30	1.906674	0.879796
NM_001109376	679692	Lpgat1	1.90076	0.874841

NM_130822	170641	Lphn3	1.894528	0.879548
NM_001169578	291006	Diras2	1.894141	0.859762
NM_017029	24588	Nefm	1.893791	0.828331
NM_013123	25663	Il1r1	1.892982	0.847444
NM_001008524	362634	C1qc	1.888248	0.905139
NM_031107	81771	Rps6ka1	1.886009	0.87254
NM_022226	63865	Lgmn	1.885267	0.901529
NM_012771	25211	Lyz2	1.873209	0.907511
NM_212525	361537	Tyrobp	1.871327	0.898627
NM_001107376	307270	Me2	1.868079	0.877248
NM_001106215	292401	Smoc2	1.86767	0.901104
NM_181478	353252	Rdh10	1.864973	0.89891
NM_001106989	302993	Sox8	1.863433	0.887442
NM_012705	24932	Cd4	1.861813	0.881884
NM_152790	260416	Carhsp1	1.85567	0.902485
NM_001014145	361237	Cdyl	1.845048	0.861921
NM_173313	286978	Tmsb11	1.841427	0.884964
NM_001109281	500762	Tmem198b	1.836452	0.872009
NM_001108550	361945	Postn	1.83487	0.876646
NM_198764	306417	Sh3rf1	1.832921	0.817216
NM_001004095	445415	S100a11	1.82705	0.90783
NM_019331	54281	Furin	1.820862	0.895335
NM_001271027	360959	Htra3	1.816485	0.898131
NM_001107524	308739	Rgma	1.806486	0.878628
NM_207601	302562	Plp2	1.806343	0.904609
NM_001100538	298510	Pabpc4	1.79907	0.888362
NM_173114	286894	Parm1	1.796819	0.863514
NM_017198	29431	Pak1	1.795795	0.844223
NM_001008300	291553	Nedd4l	1.795099	0.882592
NM_001109327	501841	Coro1c	1.782592	0.898981
NM_001017464	361658	Btbd16	1.779043	0.836118
NM_001013062	445442	Thbs1	1.778491	0.895335
NM_001105814	287537	Abr	1.771704	0.890627
NM_001106607	297433	Podxl2	1.771504	0.868257
NM_001007729	360918	Pf4	1.770433	0.879371
NM_017035	24655	Plcd1	1.768508	0.894379
NM_001106123	291327	Mrc1	1.767822	0.831835
NM_001044287	685433	Evi2a	1.7658	0.838879
NM_001009422	313211	Nipsnap3b	1.763953	0.859479
NM_138524	171553	A3galt2	1.760926	0.886557
NM_001191568	681050	Pmf1	1.76025	0.833852
NM_001025721	361289	Colec12	1.759432	0.890061
NM_001012164	361383	Cd97	1.755106	0.885424
NM_031140	81818	Vim	1.752184	0.905458

NM_001024990	302972	Amdhd2	1.751479	0.847728
NM_001044265	499991	Steap4	1.750044	0.900573
NM_001134883	314941	Lrp12	1.746532	0.871195
NM_017237	29545	Uchl1	1.741204	0.899299
NM_001005891	362431	Clec4a3	1.73054	0.84525
NM_133624	171164	Gbp2	1.730506	0.898981
NM_001127531	302671	Apls2	1.72895	0.880504
NM_001277055	310738	Ngf	1.727681	0.801041
NM_013044	25566	Tmod1	1.727493	0.852506
NM_001134863	306459	Stox2	1.724477	0.866806
NM_001004089	361710	Sipa1	1.717268	0.894875
NM_172322	282817	Pycard	1.716667	0.860859
NM_053959	117028	Bin1	1.715201	0.889318
NM_198755	301097	Vwa5a	1.709227	0.863939
NM_017187	29395	Hmgb2	1.701825	0.884893
NM_001107821	311821	Agpat2	1.701247	0.875584
NM_001108513	361689	Unc93b1	1.696421	0.878805
NM_001007557	316137	Emr1	1.694245	0.801359
NM_001024745	291733	Slc39a6	1.693972	0.879407
NM_001106998	303113	Jade2	1.693955	0.875407
NM_213561	406195	Tcf19	1.690917	0.874239
NM_012673	24832	Thy1	1.689569	0.885176
NM_133416	170929	Bcl2a1	1.688974	0.817641
NM_017015	24434	Gusb	1.6826	0.869744
NM_001108426	361308	Kif20a	1.677571	0.82426
NM_001134520	363135	Rad5412	1.675079	0.835764
NM_001106335	293733	Incenp	1.674204	0.806881
NM_001108243	317181	Srpx2	1.673562	0.862169
NM_001108630	362374	Vopp1	1.673473	0.869708
NM_001107163	304655	Zswim4	1.673399	0.832578
NM_199093	295703	Serping1	1.669287	0.899405
NM_001012092	311549	Xkr7	1.664021	0.840082
NM_001107205	305142	Abcg313	1.663095	0.85417
NM_001106127	291355	Prtfdc1	1.661159	0.883796
NM_001013146	25446	Fosl2	1.661119	0.833605
NM_001014269	367314	Lrrfip1	1.660595	0.871903
NM_012620	24617	Serpine1	1.652663	0.877212
NM_001013970	303378	Slfn13	1.649455	0.884929
NM_001025418	315648	Ppp2r1b	1.645195	0.858736
NM_001037775	304322	Chst12	1.640066	0.885247
NM_001034188	309651	Ppil1	1.639288	0.853957
NM_001107124	304315	Ttyh3	1.637543	0.876717
NM_133410	170923	Rap2b	1.636168	0.809005
NM_001012470	314329	Irf2bp1	1.635901	0.888468

NM_001134572	361224	RGD1306058	1.632778	0.88999
NM_001025715	360646	Limd2	1.632326	0.862771
NM_012904	25380	Anxa1	1.631288	0.896715
NM_019179	29261	Tyms	1.628315	0.805253
NM_134373	171386	Avpi1	1.622793	0.886875
NM_020097	56819	Extl3	1.619668	0.882699
NM_031024	81653	Dbn1	1.613483	0.887229
NM_001108393	361103	Zmiz1	1.612276	0.889778
NM_030842	81008	Itga7	1.610864	0.895229
NM_017125	29186	Cd63	1.606973	0.897777
NM_001107657	310178	Myo10	1.59927	0.838772
NM_001013127	304983	Tagln2	1.599252	0.897388
NM_001106098	290959	Slc35d2	1.593062	0.830278
NM_080782	114851	Cdkn1a	1.582117	0.857638
NM_001100778	399489	E2f1	1.581591	0.809075
NM_172045	259225	Ppp1r14b	1.576401	0.888043
NM_021868	60465	Cttn	1.574036	0.888999
NM_001030027	290842	Gins4	1.571964	0.803766
NM_001025155	502715	Lrrc17	1.571757	0.85686
NM_001271080	292735	Shkbp1	1.568119	0.839126
NM_183330	252929	Ctsz	1.566114	0.878699
NM_030995	25152	Map1a	1.564911	0.872929
NM_001107293	306261	Eaf1	1.564388	0.827021
NM_001108308	360667	Mfsd11	1.563215	0.811872
NM_001007710	315939	Pxylp1	1.559787	0.859054
NM_001024279	448830	Ly96	1.557625	0.849887
NM_001012174	361810	Fkbp5	1.556861	0.861921
NM_017133	29220	Thbs4	1.549836	0.89091
NM_022538	64369	Ppap2a	1.548811	0.888999
NM_001130988	681062	Slbp	1.547758	0.843869
NM_001191856	85272	Bmp7	1.546029	0.827233
NM_001048047	682105	Reep2	1.54515	0.838666
NM_001100635	25119	Mr1	1.540442	0.828366
NM_001106833	300803	Lactb	1.534016	0.836684
NM_139342	246240	Ripk3	1.533959	0.8198
NM_022597	64529	Ctsb	1.533701	0.891335
NM_031645	58965	Ramp1	1.531295	0.881566
NM_001025137	498256	Ier5	1.52675	0.872044
NM_001134980	313373	Cyp2j10	1.526172	0.864364
NM_001008379	363004	Prr13	1.525232	0.886627
NM_021581	59101	Leprel4	1.522437	0.856895
NM_001191969	499975	Fam126a	1.517597	0.852258
NM_001107979	313582	Fhl3	1.516373	0.867549
NM_016990	24170	Add1	1.514817	0.889282

NM_001085381	266706	Gjc1	1.510756	0.832189
NM_181634	314614	Lppr3	1.510015	0.863408
NM_001024779	310848	Cyp2u1	1.509894	0.863656
NM_001108313	360697	Ifngr2	1.509609	0.871832
NM_031092	81756	Rab13	1.508084	0.867266
NM_001014790	310486	Rarres1	1.50392	0.830702
NM_001037660	652957	Csf2ra	1.501769	0.840471
NM_001107309	306375	Csgalnact1	1.501366	0.848294
NM_001108902	365042	Rab32	1.501131	0.825853
NM_001108192	316033	Glb1	1.500028	0.868611
NM_001108703	362706	Rbks	1.497051	0.828932
NM_012802	25267	Pdgfra	1.496221	0.872646
NM_001015020	316742	Tgif1	1.494908	0.839303
NM_001106173	291914	Cnep1r1	1.494842	0.878451
NM_001005384	310132	Osmr	1.493373	0.809252
NM_133596	171129	Uggt1	1.491044	0.846276
NM_053484	85246	Gas7	1.490716	0.889105
NM_173116	286896	Sgpl1	1.487816	0.867726
NM_001013236	685579	Rrm1	1.486253	0.856435
NM_019141	25565	Tle4	1.483911	0.808368
NM_001107137	304423	Tyw1	1.483734	0.849497
NM_001004226	295231	Glmp	1.479921	0.877814
NM_001079894	361659	Plekha1	1.478565	0.87831
NM_145877	113886	Kif1c	1.47649	0.881424
NM_001034002	363014	Yap1	1.473152	0.876752
NM_019316	54264	Mafb	1.472808	0.821535
NM_138889	192248	Cdh13	1.471734	0.864718
NM_024160	79129	Cyba	1.470024	0.871337
NM_001271261	554172	Pxdn	1.468948	0.86539
NM_173134	286919	Cdk5rap2	1.464891	0.814066
NM_001079941	362095	Gtf3c5	1.459585	0.859762
NM_020100	56820	Ramp3	1.457832	0.848966
NM_001008508	288719	Tpst2	1.455101	0.868222
NM_001009271	290558	Nt5dc2	1.450383	0.847267
NM_001170435	293155	Anapc15	1.450211	0.837109
NM_058208	84607	Socs2	1.447839	0.831941
NM_001013220	362956	Triobp	1.446894	0.872859
NM_023985	78969	Trib1	1.443213	0.819376
NM_001127337	499073	Brsk1	1.43659	0.804793
NM_001108866	364380	Abhd4	1.434499	0.88238
NM_001004238	299799	Rab21	1.430571	0.876363
NM_001108740	362859	Ckap4	1.429293	0.871903
NM_001100655	289786	Nacad	1.428051	0.809713
NM_001109274	500616	Socs5	1.426975	0.867939

NM_177419	363247	Xrcc5	1.426243	0.849073
NM_017113	29143	Grn	1.41879	0.815942
NM_053586	94194	Cox5b	1.417716	0.883017
NM_130400	24312	Dhfr	1.415156	0.833569
NM_001100970	305494	Aebp1	1.414097	0.881283
NM_001108747	362886	Rassf3	1.410964	0.863196
NM_053765	114711	Gne	1.409605	0.839445
NM_001108472	361514	Meis3	1.405825	0.805005
NM_019275	50554	Smad4	1.404602	0.87123
NM_001025675	307351	Tubb6	1.40455	0.868611
NM_019152	29153	Capn1	1.404207	0.869213
NM_001191753	312846	Rassf8	1.401961	0.857461
NM_001008363	360772	Zfand2a	1.401237	0.83449
NM_172222	24231	C2	1.398602	0.861461
NM_017196	29427	Aif1	1.39784	0.85509
NM_138538	171574	Dnm3	1.396213	0.835941
NM_053827	116552	Plod1	1.395385	0.877
NM_019153	29158	Fbln5	1.393896	0.871195
NM_001191577	314623	Midn	1.391406	0.863726
NM_001004087	360942	Pcdh7	1.387464	0.812615
NM_001191737	295654	Ttc21b	1.383101	0.80681
NM_001012167	361527	Pld3	1.380613	0.872044
NM_001106286	293186	Lyve1	1.374363	0.837958
NM_139259	246143	Nradd	1.373395	0.811128
NM_001135749	499655	Cks1b	1.371628	0.820154
NM_001002016	60374	Lmna	1.370948	0.878557
NM_001014787	304979	Igsf8	1.368422	0.858063
NM_012817	25285	Igfbp5	1.36422	0.877602
NM_001134979	312299	Ezh2	1.362481	0.811518
NM_001014090	315594	Oaf	1.362371	0.877071
NM_033651	29728	Mcm4	1.361933	0.815801
NM_134334	171293	Ctsd	1.36175	0.879053
NM_012923	25405	Ccng1	1.360146	0.870204
NM_030826	24404	Gpx1	1.359861	0.876575
NM_024366	65153	Ncs1	1.359316	0.836401
NM_001009536	494338	Trim25	1.358606	0.81173
NM_012775	29591	Tgfbr1	1.357635	0.845108
NM_001109508	688721	Lrfn4	1.35658	0.847975
NM_001004203	288532	Mcm7	1.354099	0.829994
NM_001106757	299314	Cfp	1.351329	0.805784
NM_053761	114636	Zyx	1.349757	0.873213
NM_001004202	287910	Ccl6	1.348818	0.861461
NM_012862	25333	Mgp	1.348035	0.879265
NM_001107400	307540	Celf4	1.34681	0.830207

NM_017147	29271	Cfl1	1.346672	0.879371
NM_145789	252963	Il13ra1	1.345935	0.840011
NM_017218	29496	Erbb3	1.345457	0.876752
NM_001109912	498545	Tsc22d1	1.342377	0.823729
NM_001106576	296758	Armc10	1.342088	0.853037
NM_001008306	294019	Calhm2	1.340688	0.809713
NM_175756	289211	Fcgr2b	1.337917	0.806385
NM_001106876	301119	Mllt1	1.335122	0.858205
NM_173136	286921	Akr1b8	1.331594	0.837321
NM_001105992	289424	Xpr1	1.330436	0.848719
NM_001106430	295037	Mgst2	1.327809	0.834985
NM_012733	25056	Rbp1	1.325587	0.868965
NM_001037185	295107	Smc4	1.324637	0.832401
NM_001106899	301337	Plekhb2	1.322565	0.867372
NM_173102	29214	Tubb5	1.322558	0.876681
NM_001099647	685045	Abracl	1.321642	0.863019
NM_001006989	301013	Shisa5	1.320927	0.872009
NM_053343	83825	Dclk1	1.31834	0.802563
NM_001108452	361422	Cotl1	1.317229	0.873814
NM_001047854	293628	Chid1	1.315127	0.8238
NM_001004274	360622	Igfbp4	1.312339	0.871974
NM_024156	79125	Anxa6	1.311942	0.874133
NM_001108666	362519	Smc2	1.311918	0.803448
NM_001191695	291406	Zfp516	1.307847	0.83318
NM_012555	24356	Ets1	1.30403	0.848117
NM_199382	296973	Bpgm	1.303227	0.86624
NM_001009268	289820	Actr2	1.303004	0.868293
NM_001173341	305373	Hmgb3	1.296985	0.831941
NM_031510	24479	Idh1	1.29311	0.854559
NM_053628	114029	Megf8	1.291386	0.838808
NM_133380	25084	Il4r	1.288555	0.812296
NM_017258	29618	Btg1	1.28835	0.87208
NM_001127529	301525	Glb1l	1.287919	0.829145
NM_001191653	303599	Tanc2	1.285113	0.835374
NM_138900	192262	C1s	1.284072	0.870699
NM_001030026	290644	Ifi30	1.283795	0.859691
NM_001025119	310674	Plekho1	1.282978	0.854984
NM_053354	84350	Dnmt1	1.2827	0.805819
NM_001025279	306014	Reep4	1.281434	0.825004
NM_001106455	295325	Igsf3	1.278382	0.823942
NM_001013191	361391	Cbfb	1.278196	0.849462
NM_130812	25164	Cdkn2b	1.275373	0.80766
NM_001136162	292095	Ttc13	1.273009	0.843268
NM_001107279	306055	Pcdh17	1.267418	0.810279

NM_001100518	292155	Hs2st1	1.265491	0.850559
NM_001106950	302495	Rap2c	1.263039	0.84479
NM_001012179	361927	Fxr1	1.262794	0.859019
NM_001191110	83465	Celsr2	1.26278	0.862983
NM_001143858	309081	Dock1	1.259595	0.849568
NM_053943	116782	Pcdhgc3	1.252807	0.836826
NM_001108331	360785	Apls1	1.24799	0.853497
NM_053570	89843	Cxadr	1.246408	0.813358
NM_001108439	361368	Large	1.245678	0.846276
NM_001271251	312607	Pdzrn3	1.245585	0.826455
NM_001244867	691657	Crip1	1.241212	0.865815
NM_138509	114764	Mapre1	1.238208	0.860789
NM_030847	81505	Emp3	1.236077	0.869071
NM_001100493	287134	Gnptg	1.233971	0.847232
NM_022943	65037	Mertk	1.232195	0.851303
NM_001033683	287721	Vat1	1.226366	0.867974
NM_001013984	304860	Npl	1.224188	0.820154
NM_001014236	364534	Ssbp4	1.223722	0.841958
NM_134353	171350	Pabpc1	1.222504	0.868151
NM_001100519	293455	Gga2	1.219766	0.802598
NM_031137	81815	Tpp2	1.217834	0.832401
NM_133569	171100	Angptl2	1.215595	0.860859
NM_001008521	315265	Twf1	1.215571	0.858736
NM_001012064	308417	Pvrl2	1.213779	0.80504
NM_001106999	303122	RGD1310352	1.212072	0.854807
NM_012529	24264	Ckb	1.211267	0.867018
NM_001173511	312083	Ccdc132	1.204782	0.810031
NM_182821	306203	Pxk	1.199764	0.827729
NM_001012044	306071	Lcp1	1.199228	0.827871
NM_138828	25728	Apoe	1.198628	0.852683
NM_001004230	296304	Edem2	1.195946	0.811589
NM_012924	25406	Cd44	1.195854	0.855975
NM_001008282	287835	Galk1	1.195187	0.821322
NM_031721	65164	Htra1	1.194712	0.865036
NM_022511	64303	Pfn1	1.193557	0.86585
NM_001013182	360492	Hn1l	1.192983	0.824862
NM_001007144	298199	Plin2	1.192274	0.848577
NM_001106196	292090	Galnt2	1.181939	0.859231
NM_001012046	306141	Spry2	1.177771	0.838312
NM_138839	192129	Vmp1	1.177649	0.852789
NM_001013157	310378	Nnt	1.176889	0.815871
NM_001134514	295678	Sestd1	1.176265	0.801642
NM_001077200	361430	Piezo1	1.175454	0.824437
NM_001130564	362923	Efr3a	1.17535	0.83941

NM_001109291	500987	H2afx	1.175083	0.838737
NM_001109885	362115	Fam129b	1.173853	0.854524
NM_001017537	297392	Tex261	1.173556	0.857709
NM_001127449	287876	Actg1	1.169334	0.864965
NM_001144861	499249	Plekha7	1.168119	0.831693
NM_001008336	303882	Tnk2	1.166157	0.8421
NM_012755	25150	Fyn	1.164374	0.854807
NM_019217	29456	Map1b	1.162588	0.858382
NM_001160228	303068	Adam19	1.162522	0.835091
NM_001108106	315217	Plxnb2	1.161122	0.842135
NM_053450	84580	Hdac5	1.155285	0.845922
NM_022512	64304	Acads	1.154372	0.806775
NM_001037768	297393	Nagk	1.150051	0.830348
NM_001106852	300974	Mrpl3	1.149647	0.830313
NM_001011959	296370	Ctsa	1.146853	0.860541
NM_199404	361378	Man2b1	1.146402	0.843162
NM_001191693	307178	Arhgap21	1.14396	0.817712
NM_139096	245955	Lgals3bp	1.142264	0.853957
NM_001107881	312652	Plxnd1	1.13613	0.848188
NM_001014120	360627	Fkbp10	1.135397	0.844719
NM_022674	58940	H2afz	1.133769	0.841392
NM_001106511	296156	Cpxm1	1.132927	0.812544
NM_001007705	314399	Ubr7	1.130218	0.804049
NM_033230	24185	Akt1	1.129514	0.855231
NM_001113791	501007	RGD1562618	1.12868	0.822915
NM_001005888	314360	Galc	1.126155	0.806138
NM_001025140	498433	Psme4	1.125391	0.826278
NM_001128155	497936	Tmem88	1.123345	0.83187
NM_031617	58822	Csnk1e	1.122285	0.835198
NM_001011946	294673	Hexb	1.121295	0.843126
NM_001047885	310326	Arse	1.12102	0.819199
NM_001108815	363447	Tspan7	1.118282	0.83226
NM_057213	117596	Atp6v1b2	1.117898	0.82964
NM_012931	25414	Bcar1	1.117713	0.830631
NM_001107596	309400	Tmem2	1.117447	0.832153
NM_053593	94201	Cdk4	1.115384	0.852435
NM_053522	85428	Rhoq	1.114975	0.835374
NM_212508	361791	Nrm	1.114605	0.800262
NM_001004231	296562	Npdc1	1.11416	0.858028
NM_031342	83510	Lypla2	1.110818	0.831339
NM_001106084	290794	Tnks	1.107836	0.808332
NM_001009543	494346	Slmo2	1.107068	0.838312
NM_001108099	314856	Mdm2	1.103338	0.809996
NM_001107716	310784	Taf13	1.103332	0.825039

NM_030992	25096	Pld1	1.101624	0.81481
NM_001126287	361790	Ppp1r18	1.100256	0.849462
NM_134396	171433	Micu2	1.100038	0.835233
NM_001134640	502525	Dnajc19	1.095626	0.801147
NM_001008348	309172	Frmd8	1.094692	0.841073
NM_001191847	361957	Mfsd1	1.094133	0.818031
NM_001011929	292156	Sh3glb1	1.088937	0.842029
NM_001106933	302290	Ap3s1	1.085221	0.832437
NM_001007606	266975	Sars	1.085019	0.84164
NM_001025718	361178	Tfdp1	1.083033	0.813854
NM_031574	29372	Rasa3	1.081013	0.824012
NM_001108870	364405	Scara3	1.080481	0.835551
NM_001108818	363485	Pbdc1	1.080294	0.80458
NM_001002851	289380	Nenf	1.080225	0.842454
NM_001170335	292721	Erf	1.076454	0.828614
NM_001106210	292306	Rnaset2	1.07247	0.839657
NM_001191630	308845	Prkrir	1.066658	0.809642
NM_001115024	290994	Lman2	1.064727	0.835693
NM_001128083	688785	Trim8	1.063177	0.834525
NM_172334	282837	Fibp	1.063105	0.82203
NM_001107624	309728	Arid5b	1.059762	0.813288
NM_001167666	100312984	Eif2s3y	1.058931	0.831092
NM_001244855	362965	Rangap1	1.0565	0.803518
NM_031695	64442	St3gal2	1.055876	0.833569
NM_001014200	362828	Sppl2b	1.055874	0.82695
NM_001100806	683788	Fscn1	1.054903	0.845285
NM_057192	117538	Wipf1	1.053272	0.834914
NM_031986	83841	Sdcbp	1.049519	0.842029
NM_173111	117557	Tpm3	1.048073	0.841286
NM_012500	24206	Apeh	1.04661	0.807129
NM_001191840	84574	Tgfb1i1	1.045509	0.825074
NM_019905	56611	Anxa2	1.044277	0.847374
NM_130416	155423	Anxa7	1.041444	0.838772
NM_021695	60324	Synpo	1.040448	0.810173
NM_001012238	304334	Fam20c	1.039757	0.82065
NM_019621	29495	Dlg4	1.039693	0.820225
NM_001030039	315707	Csk	1.037736	0.826313
NM_013150	497815	Nrcam	1.034491	0.814314
NM_001106097	290939	Med10	1.030997	0.821606
NM_001037787	360762	Rpo1-3	1.030615	0.816048
NM_001009641	292808	Pepd	1.030416	0.825428
NM_001079705	292139	RGD1311558	1.0297	0.83141
NM_175754	25592	Agrn	1.029588	0.815341
NM_001108817	363478	Snx12	1.0282	0.812615

NM_053290	24642	Pgam1	1.025819	0.832578
NM_001127600	682999	Aida	1.02344	0.835127
NM_053439	84509	Ran	1.023024	0.837392
NM_021261	50665	Tmsb10	1.022358	0.84663
NM_001108373	361028	Mapk1ip1l	1.021684	0.826738
NM_053445	84575	Fads1	1.020311	0.843834
NM_001002802	288227	Bace2	1.018177	0.835021
NM_012801	25266	Pdgfa	1.018175	0.831021
NM_031145	81823	Cib1	1.01617	0.804793
NM_212498	361789	Atat1	1.01376	0.804651
NM_001134555	312705	C1r	1.011858	0.835764
NM_199256	80843	Sec61a1	1.010476	0.828402
NM_001012091	311547	Foxs1	1.007711	0.808722
NM_001134873	313560	Ctps1	1.007516	0.810597
NM_017182	29384	H2afy	1.006224	0.817818
NM_031797	83628	Cd82	1.005622	0.831481
NM_001011947	294804	Rai14	1.002095	0.819694
NM_001127544	310791	Slc25a24	1.000012	0.800651
NM_184046	297383	Rtkn	-1.01145	0.809429
NM_001017446	291737	Ino80c	-1.01333	0.809429
NM_131906	170698	Slco1a2	-1.02261	0.807518
NM_001005762	282843	Sorbs3	-1.02925	0.830879
NM_001108273	360519	Rasgef1c	-1.03413	0.820898
NM_001037097	366697	Sptlc2	-1.03519	0.821039
NM_198779	362219	Gpcpd1	-1.03633	0.81258
NM_001014071	313729	Errfi1	-1.03714	0.81888
NM_012716	25027	Slc16a1	-1.03735	0.820402
NM_022005	63847	Fxyd6	-1.03909	0.846029
NM_001191618	302612	Tspyl2	-1.04077	0.800333
NM_001008360	360480	Cdip1	-1.04112	0.810491
NM_001106158	291694	Fam13b	-1.04449	0.815624
NM_001191846	84482	Foxo1	-1.04573	0.806633
NM_031814	83709	Git1	-1.04768	0.842631
NM_001100885	296313	Myl9	-1.04989	0.836224
NM_012576	24413	Nr3c1	-1.05112	0.820685
NM_001004245	300519	Esam	-1.05363	0.805394
NM_001105837	287703	Eif1	-1.05404	0.848046
NM_057211	117560	Klf9	-1.05702	0.827906
NM_001107954	313488	Reck	-1.06392	0.826915
NM_012925	25407	Cd59	-1.06773	0.845108
NM_001107381	307348	Spire1	-1.07429	0.814279
NM_001134754	362713	Seli	-1.07454	0.814987
NM_001014227	363644	Glod4	-1.07527	0.839764
NM_001107026	303351	Rhot1	-1.077	0.825074

NM_024131	29318	Ddt	-1.07706	0.841569
NM_001013912	295264	Mllt11	-1.07999	0.805854
NM_001107188	304893	Rasal2	-1.08374	0.832047
NM_001037349	293938	Bloc1s2	-1.08882	0.838525
NM_001044275	679532	Elovl1	-1.0897	0.84886
NM_012504	24211	Atp1a1	-1.09122	0.84479
NM_001015027	362453	Crebl2	-1.09514	0.813358
NM_031576	29441	Por	-1.09597	0.838631
NM_001025703	315160	Desi1	-1.09697	0.848223
NM_177425	29317	Csrp2	-1.10269	0.81711
NM_001004213	290796	Saraf	-1.10343	0.851586
NM_001137643	499422	Gstt3	-1.10488	0.806279
NM_001106288	293410	Swap70	-1.10751	0.82957
NM_181371	297029	Gstk1	-1.1108	0.810244
NM_001109444	681578	Rnf13	-1.11101	0.853532
NM_001271239	500921	Tmem117	-1.11538	0.817924
NM_001030033	302499	C1galt1c1	-1.11957	0.823305
NM_053796	116479	F11r	-1.12031	0.801784
NM_001271384	316021	Epm2aip1	-1.12052	0.811695
NM_001012119	315136	Cbx6	-1.12125	0.837781
NM_021594	59114	Slc9a3r1	-1.12237	0.84164
NM_172030	64467	Entpd2	-1.12422	0.858736
NM_001004209	289456	Hsd17b11	-1.12434	0.856329
NM_012847	25318	Fnta	-1.13211	0.855231
NM_001107407	307618	Cdh5	-1.13371	0.834277
NM_001106524	296259	Acss1	-1.13389	0.802103
NM_031028	81657	Gabbr1	-1.13413	0.851798
NM_031561	29184	Cd36	-1.13644	0.82065
NM_001007647	297337	Rnf181	-1.13745	0.834844
NM_172062	64475	P4ha1	-1.1386	0.847798
NM_012778	25240	Aqp1	-1.13912	0.84971
NM_001024757	297930	Wwp1	-1.1407	0.835693
NM_001009692	311848	Sh3glb2	-1.14199	0.854701
NM_001107613	309639	Anks1a	-1.14467	0.832472
NM_001191927	498081	Slc35a5	-1.14515	0.814385
NM_012544	24310	Ace	-1.15074	0.804439
NM_201272	310999	Plekhg5	-1.1511	0.810137
NM_001034004	619374	Jam2	-1.15369	0.827481
NM_022592	64524	Tkt	-1.15418	0.859196
NM_199499	361549	Lgi4	-1.15648	0.862027
NM_001106749	299207	RGD1310769	-1.16054	0.82688
NM_001127580	690163	Oxct1	-1.16229	0.81265
NM_019318	54267	Maf	-1.16312	0.854559
NM_012595	24534	Ldhb	-1.1691	0.861178

NM_053882	116662	Ecm1	-1.16917	0.85155
NM_054011	117186	Sh3bp5	-1.16949	0.826596
NM_001009966	311187	Pacsin3	-1.17041	0.81573
NM_001106839	300839	Lysmd2	-1.17602	0.81488
NM_053367	84380	Dhh	-1.177	0.859337
NM_031327	83476	Cyr61	-1.18093	0.860895
NM_053713	114505	Klf4	-1.18387	0.842241
NM_172336	282840	Atf5	-1.18408	0.860328
NM_001106621	297601	Mrpl51	-1.19034	0.840118
NM_001109062	498153	Gpr146	-1.19848	0.804651
NM_001108286	360590	Ypel2	-1.1998	0.835021
NM_019312	54260	Itpkb	-1.20195	0.845073
NM_012913	25390	Atp1b3	-1.20774	0.86493
NM_001105797	287450	Slc16a11	-1.21042	0.830454
NM_134449	85332	Prkcdbp	-1.21298	0.86631
NM_001009603	293653	Acy3	-1.21669	0.813323
NM_001038615	308099	Fndc1	-1.2215	0.803625
NM_001137564	689415	Mt2A	-1.2216	0.858452
NM_001077643	100359982	Mpc2	-1.22166	0.846206
NM_031667	60568	Syt11	-1.22209	0.848789
NM_020087	56761	Notch3	-1.22863	0.810633
NM_001025766	498951	Il34	-1.23134	0.850488
NM_145674	246766	Ggta1p	-1.23157	0.837038
NM_001008364	361328	Snx24	-1.2341	0.833215
NM_001271410	100360066	Evi5	-1.2347	0.855798
NM_001039023	307098	Net1	-1.23549	0.842808
NM_019239	29582	Mgat3	-1.23827	0.85509
NM_022501	338401	Crip2	-1.23929	0.869213
NM_001008361	360554	Rnf167	-1.24473	0.857568
NM_001106434	295061	Tm4sf1	-1.24564	0.841993
NM_001100673	302983	Mapk8ip3	-1.25428	0.837073
NM_001105965	289178	Dpt	-1.25826	0.848365
NM_001007728	360871	Mpzl1	-1.25912	0.850984
NM_053455	84586	Fgl2	-1.26103	0.871124
NM_001107288	306204	Flnb	-1.26367	0.859444
NM_001014136	361014	Ngly1	-1.26491	0.844188
NM_031134	81812	Thra	-1.26562	0.824437
NM_001127530	302022	LOC302022	-1.26743	0.819517
NM_001100789	498331	Ptpn13	-1.26831	0.838029
NM_001001800	308900	rnf141	-1.27083	0.856789
NM_017345	50687	L1cam	-1.27121	0.853497
NM_031762	83571	Cdkn1b	-1.27214	0.869071
NM_022294	64124	Eltd1	-1.27264	0.828862
NM_021863	60460	Hspa2	-1.27446	0.869921

NM_001109382	679921	Vasn	-1.27462	0.82065
NM_001108831	363634	Kctd11	-1.27798	0.841463
NM_145096	246326	Zdhhc2	-1.27897	0.862558
NM_013180	25724	Itgb4	-1.28405	0.872929
NM_024360	29577	Hes1	-1.28423	0.826809
NM_001127546	315131	Kdelr3	-1.28489	0.867266
NM_001002805	406161	C4b	-1.29056	0.8318
NM_012656	24791	Sparc	-1.29123	0.874558
NM_001008297	290529	Fam213a	-1.29256	0.845073
NM_053363	84360	Clcn3	-1.2926	0.856789
NM_138826	24567	Mt1a	-1.29681	0.872186
NM_001191910	140589	Gli1	-1.29709	0.831693
NM_001135871	310856	Ppa2	-1.30229	0.846453
NM_033653	29253	Maoa	-1.30481	0.856789
NM_177421	305886	Slc22a17	-1.30768	0.871691
NM_031057	81708	Aldh6a1	-1.30785	0.859019
NM_001011933	293173	Far1	-1.31316	0.858806
NM_001006991	305149	Nudt9	-1.31496	0.860966
NM_012796	29487	Gstt2	-1.31618	0.807553
NM_001107626	309732	Ado	-1.31649	0.842347
NM_001271344	100359680	Lpcat2	-1.32687	0.859267
NM_001044284	684980	Tsc22d4	-1.32827	0.86808
NM_001109120	498741	Mboat1	-1.32992	0.847728
NM_017086	25148	Egr3	-1.33074	0.8381
NM_031525	24629	Pdgfrb	-1.33192	0.859373
NM_001106468	295425	Usp53	-1.33549	0.808049
NM_031752	78958	Bcam	-1.34552	0.867337
NM_031699	65129	Cldn1	-1.34631	0.860364
NM_001077642	54249	Cfd	-1.34796	0.869673
NM_012797	25261	Id1	-1.34821	0.856506
NM_001013138	306771	Tspan17	-1.35161	0.86939
NM_023957	66013	Arhgef9	-1.35357	0.809146
NM_031004	81633	Acta2	-1.36513	0.875442
NM_001106061	290541	Arhgef3	-1.36745	0.82964
NM_012507	24214	Atp1b2	-1.3677	0.875655
NM_130829	170673	Palm	-1.36962	0.869354
NM_001014070	313672	Kazn	-1.37812	0.802492
NM_022632	360272	Slit2	-1.38265	0.850418
NM_001013171	314543	Gulp1	-1.38653	0.858523
NM_001105879	288093	Arhgap31	-1.39177	0.869071
NM_057104	84050	Enpp2	-1.39317	0.842064
NM_080698	64507	Fmod	-1.40014	0.83679
NM_001033663	64363	Araf	-1.4033	0.813995
NM_001012121	315189	Prr5	-1.40717	0.854276

NM_001007656	298848	Mapre3	-1.40774	0.869177
NM_001001515	361084	Lmo7	-1.40847	0.808969
NM_181381	312382	Abcg2	-1.41319	0.804474
NM_031549	25123	Tagln	-1.41499	0.877885
NM_001013244	366602	Tspan13	-1.41531	0.858983
NM_022856	64824	Nab1	-1.41596	0.844861
NM_178330	291946	Tmem184c	-1.41745	0.85955
NM_001011952	295455	Slc39a8	-1.4213	0.840542
NM_053582	94174	Tinagl1	-1.42275	0.873213
NM_138905	192270	Ppap2b	-1.42596	0.876575
NM_022224	63852	Pter	-1.42618	0.818172
NM_017259	29619	Btg2	-1.42756	0.86762
NM_145682	246776	Filip1	-1.4288	0.85555
NM_012828	25297	Cacnb3	-1.43143	0.871089
NM_139189	246046	Lmbrd1	-1.43379	0.871018
NM_206950	404280	Mid1ip1	-1.43518	0.874912
NM_139116	245983	Il11ra1	-1.43568	0.854665
NM_199109	314323	Flvcr2	-1.4357	0.805076
NM_138843	192172	Mpst	-1.43669	0.840082
NM_021840	64646	Hist3h2a	-1.43877	0.828331
NM_001034083	29140	Snn	-1.44222	0.876504
NM_017274	29653	Gpam	-1.4439	0.849781
NM_001105737	89804	Tek	-1.44673	0.841392
NM_001108272	360511	Pank3	-1.44708	0.877672
NM_053811	116501	Slc9a3r2	-1.44721	0.87523
NM_031591	29583	Pecam1	-1.44977	0.86854
NM_001108509	361676	Pnpla2	-1.45043	0.874982
NM_001106009	289664	Ldb2	-1.45702	0.855904
NM_022193	60581	Acaca	-1.46256	0.85194
NM_001025775	501194	Ppip5k2	-1.46306	0.842843
NM_017301	29733	S1pr1	-1.46393	0.843622
NM_139325	24334	Eno2	-1.47063	0.87569
NM_012600	24552	Me1	-1.47741	0.842347
NM_012498	24192	Akr1b1	-1.47921	0.88553
NM_001034125	287422	Per1	-1.47989	0.855125
NM_001033682	287479	Shpk	-1.48131	0.842808
NM_001105874	288057	Mylk	-1.48387	0.816863
NM_001105759	259237	Dock9	-1.48518	0.853002
NM_001047085	289089	Ivns1abp	-1.48629	0.887654
NM_001024864	287061	Rogdi	-1.48881	0.858771
NM_001004269	315509	Jam3	-1.49286	0.873425
NM_031753	79559	Alcam	-1.49331	0.867408
NM_001107096	304007	Cep97	-1.49397	0.831092
NM_001004228	295490	Emcn	-1.49544	0.870522

NM_013155	25696	Vldlr	-1.49818	0.802209
NM_001108938	365691	Egflam	-1.49843	0.806102
NM_133651	25404	Cav1	-1.49849	0.802881
NM_130403	114004	Ppp1r14a	-1.50014	0.800014
NM_001011921	290771	Pdgfrl	-1.50325	0.804722
NM_031321	83467	Slit3	-1.50716	0.846029
NM_001009391	305177	Enoph1	-1.50776	0.878628
NM_001130542	299940	Sntb1	-1.51115	0.864576
NM_001007092	313717	Clstn1	-1.5176	0.888079
NM_012988	25492	Nfia	-1.51782	0.874062
NM_001015012	308821	Rab30	-1.51847	0.812544
NM_001107681	310506	Ppm1l	-1.52445	0.860824
NM_001109447	683713	Gal3st1	-1.5281	0.856223
NM_199117	362962	Cbx7	-1.5332	0.851692
NM_001011917	290291	Cab39l	-1.53466	0.878026
NM_001134696	100188934	Ctxn3	-1.54209	0.842595
NM_022623	64558	Fzd4	-1.54283	0.802598
NM_001024274	362129	Gtdc1	-1.54621	0.846984
NM_053350	84114	Agps	-1.54791	0.870699
NM_181474	308140	Tfb1m	-1.55593	0.865921
NM_019196	29365	Mpdz	-1.55611	0.865036
NM_001107698	310678	Hist2h3c2	-1.55674	0.805961
NM_001100666	295105	Schip1	-1.5583	0.876575
NM_019156	29169	Vtn	-1.5664	0.859762
NM_001106276	293056	Cpeb1	-1.57708	0.848648
NM_001025415	309527	Ch25h	-1.57766	0.81757
NM_001109364	503306	Sowahc	-1.58179	0.853426
NM_022245	64001	Cyb5a	-1.58392	0.892149
NM_001013427	297073	Rarres2	-1.5895	0.884893
NM_022602	64534	Pim3	-1.59067	0.822172
NM_001003403	444983	Apold1	-1.59475	0.869
NM_001109559	689959	LOC689959	-1.59516	0.804863
NM_001009292	293489	Slx1b	-1.59784	0.849108
NM_001106306	293566	Cpxm2	-1.60494	0.863266
NM_198738	293820	Psat1	-1.6071	0.869956
NM_001001972	406866	Ly6g6e	-1.6076	0.843091
NM_001108084	314751	Tmcc3	-1.62307	0.846913
NM_020086	56765	Plvap	-1.62817	0.889813
NM_001034081	313615	Paqr7	-1.63065	0.877637
NM_001101005	689852	Psmf1	-1.63142	0.892999
NM_001007684	306330	Klf2	-1.63261	0.88107
NM_057137	117278	Ebp	-1.634	0.87031
NM_031063	81727	Mvk	-1.63615	0.867337
NM_001014762	296278	Pdrg1	-1.63848	0.889636

NM_032084	84031	Chn2	-1.64445	0.875938
NM_022297	64157	Ddah1	-1.64511	0.894202
NM_138848	192181	Podxl	-1.64602	0.873637
NM_001024371	553106	Ncald	-1.64619	0.83233
NM_001008334	303330	Tmem97	-1.64639	0.879407
NM_053935	116743	Sh3gl2	-1.64808	0.847409
NM_053770	114901	Sorbs2	-1.65581	0.851515
NM_001107303	306344	Arrdc2	-1.65629	0.850382
NM_031817	83717	Omd	-1.65992	0.851727
NM_145089	246307	Asrgl1	-1.6642	0.877885
NM_031701	65131	Cldn5	-1.66596	0.881672
NM_001113357	29358	Npy1r	-1.66633	0.810987
NM_001109050	498014	RGD1565033	-1.67729	0.886026
NM_134336	171297	Nlgn3	-1.67925	0.83449
NM_013145	25686	Gnai1	-1.68089	0.889282
NM_001244933	100361818	Nfatc1	-1.68436	0.878239
NM_001108415	361251	Elmo1	-1.68467	0.885424
NM_001276715	85421	Prkd1	-1.68522	0.838772
NM_031731	65183	Aldh3a2	-1.68731	0.886203
NM_013062	25589	Kdr	-1.68954	0.844613
NM_001107227	305454	Zfyve28	-1.69633	0.81219
NM_001106120	291309	Usp6nl	-1.70481	0.888716
NM_181386	353229	Sgms1	-1.70482	0.877248
NM_022389	64191	Dhcr7	-1.70583	0.873
NM_001001969	294241	Ly6g6c	-1.70738	0.839728
NM_031716	65154	Wisp1	-1.70779	0.84971
NM_001002289	432392	Fut8	-1.71517	0.900538
NM_001106683	298504	Mfsd2a	-1.71776	0.844825
NM_031011	81640	Amd1	-1.71898	0.8833
NM_172317	116831	Fxyd3	-1.71971	0.894485
NM_053703	114495	Map2k6	-1.72153	0.834702
NM_001108654	362481	Tox	-1.72577	0.846241
NM_031798	83629	Slc12a2	-1.73	0.891335
NM_001033694	300095	Srebf2	-1.73057	0.8936
NM_001007675	303701	C1qtnf1	-1.73206	0.886274
NM_206845	298859	Dnajc27	-1.73442	0.811836
NM_022177	24772	Cxcl12	-1.7393	0.868151
NM_001048044	313838	Cdc42ep3	-1.74285	0.85109
NM_001100576	307096	Akr1c19	-1.74606	0.891866
NM_012914	25391	Atp2a3	-1.74903	0.83049
NM_001034012	310670	Adamts14	-1.75359	0.880292
NM_017024	24530	Lcat	-1.7553	0.873177
NM_031726	65171	Scamp5	-1.75636	0.882522
NM_001126093	685448	Pcp4l1	-1.75769	0.86808

NM_001106082	290705	Nek1	-1.76525	0.881141
NM_001025112	288611	Wbscr17	-1.76667	0.855019
NM_001128194	688993	Kctd7	-1.77255	0.821499
NM_001109383	679942	Angptl1	-1.77959	0.806315
NM_017000	24314	Nqo1	-1.78042	0.861497
NM_012634	24689	Prps2	-1.78104	0.886592
NM_138518	171547	Crispld2	-1.78275	0.897529
NM_139110	245977	Gpr116	-1.78404	0.875867
NM_031613	58814	Tmod2	-1.78624	0.893671
NM_001171177	299762	Tmtc2	-1.79235	0.820579
NM_001008352	310645	Pmvk	-1.7926	0.894202
NM_001127565	500464	Epb41l4b	-1.79691	0.891724
NM_001009620	287129	Tmem204	-1.79701	0.854063
NM_001107764	311341	Pla2g4b	-1.80351	0.869036
NM_013149	25690	Ahr	-1.81134	0.875903
NM_001191682	305467	Sfi1	-1.81825	0.869177
NM_138533	171569	Spon2	-1.82295	0.901812
NM_024364	60563	Hr	-1.82594	0.872115
NM_053926	116723	Pip4k2a	-1.83058	0.897742
NM_012848	25319	Fth1	-1.83075	0.908148
NM_001131013	303798	Arvcf	-1.83159	0.891618
NM_001012000	300900	Plscr4	-1.83323	0.883973
NM_001001799	308134	Tmem35	-1.83503	0.880752
NM_023090	29452	Epas1	-1.8383	0.902131
NM_181368	290553	Mustn1	-1.84082	0.873531
NM_001106769	299611	Apc2	-1.84751	0.836401
NM_031765	83574	Rxrg	-1.84956	0.882663
NM_001106304	293546	Gprc5b	-1.85326	0.900715
NM_031509	24421	Gsta1	-1.8627	0.887229
NM_001170600	24582	Myh11	-1.86325	0.882274
NM_001109010	367323	Nudt12	-1.86386	0.858771
NM_001106483	295647	Gca	-1.86514	0.877212
NM_031518	24560	Cd200	-1.86759	0.90075
NM_001013192	361621	Olfml1	-1.86859	0.894308
NM_001014140	361118	RGD1309676	-1.87128	0.845781
NM_001012163	361303	Lims2	-1.87284	0.906095
NM_001107445	307997	Hspa12a	-1.87691	0.901848
NM_001007712	316384	Sdpr	-1.8786	0.892999
NM_001191872	362734	Stxbp6	-1.88051	0.831516
NM_133623	171163	Slc6a13	-1.88234	0.821075
NM_001108533	361840	Spock2	-1.89819	0.886663
NM_017312	29884	Bok	-1.89848	0.876929
NM_001007721	360468	Emp2	-1.8993	0.908396
NM_001135778	680409	Prodh	-1.91123	0.851904

NM_001105983	289338	Disp1	-1.91295	0.887229
NM_001108621	362336	Fam180a	-1.91604	0.846701
NM_017094	25235	Ghr	-1.91752	0.863231
NM_001013967	303211	Mmgt2	-1.91911	0.886415
NM_012999	25507	Pcsk6	-1.92447	0.86178
NM_198761	304135	Adamts5	-1.92476	0.874204
NM_001134993	499566	Car13	-1.92646	0.879194
NM_001109338	502421	Slc35f1	-1.92797	0.890769
NM_001013231	364052	Pea15	-1.93086	0.911015
NM_012868	25339	Npr3	-1.93291	0.865602
NM_080892	140927	Selenbp1	-1.93702	0.876327
NM_053018	24936	Cd9	-1.94555	0.911334
NM_031582	29473	Aoc3	-1.95284	0.892362
NM_017332	50671	Fasn	-1.95949	0.905493
NM_053758	114633	Plce1	-1.96277	0.814526
NM_030865	81523	Myoc	-1.97213	0.910166
NM_145784	252939	Gpr37l1	-1.97646	0.908608
NM_053492	85254	Slc44a1	-1.97826	0.902626
NM_031048	81680	Lifr	-1.97935	0.859302
NM_012837	25307	Cst3	-1.98814	0.912997
NM_001003929	313173	Cntfr	-1.98881	0.869921
NM_017214	29480	Rgs4	-1.99101	0.825039
NM_053544	89803	Sfrp4	-1.9915	0.901564
NM_001107269	305956	Wdfy2	-1.9936	0.887229
NM_001039163	360576	Tusc5	-1.99453	0.824083
NM_177426	24424	Gstm2	-1.99711	0.90337
NM_131914	363425	Cav2	-1.99795	0.908679
NM_001135009	290905	Col4a1	-2.00368	0.911865
NM_017256	29610	Tgfbr3	-2.00749	0.909741
NM_001100690	308572	Myh14	-2.01302	0.900892
NM_031031	81660	Gatm	-2.01601	0.913988
NM_001271081	308571	Lrrc4b	-2.02127	0.9016
NM_001108015	313974	Trib2	-2.02228	0.884362
NM_080894	140929	Pde7b	-2.02595	0.810845
NM_001276721	85420	Prkcq	-2.02718	0.899264
NM_022225	25075	Htr1b	-2.02828	0.852931
NM_001024267	317409	MGC109340	-2.02935	0.884114
NM_001108233	316639	Farp2	-2.02967	0.864151
NM_024388	79240	Nr4a1	-2.03095	0.913564
NM_001106892	301261	Enpp4	-2.03338	0.902626
NM_199101	308584	Plekha4	-2.04105	0.915263
NM_032083	84030	Chn1	-2.0439	0.899441
NM_017171	29340	Prkce	-2.04984	0.812827
NM_001107587	309349	Slitrk2	-2.05514	0.854488

NM_178105	306439	Gpm6a	-2.06299	0.905564
NM_001100901	362242	Snta1	-2.07011	0.90783
NM_024353	25031	Plcb4	-2.07623	0.855762
NM_001271381	364952	Nkd1	-2.07865	0.896821
NM_134454	89805	Angpt2	-2.07947	0.854842
NM_033234	24440	Hbb	-2.08201	0.909245
NM_001079888	303384	Mmp28	-2.08506	0.879619
NM_021760	60379	Col5a3	-2.09068	0.915581
NM_017174	29354	Pla2g5	-2.09473	0.891866
NM_001037218	304299	Radil	-2.09883	0.836047
NM_013096	25632	Hba1	-2.09914	0.917386
NM_001002830	305302	Rasl11b	-2.1008	0.870735
NM_001106159	291699	Stard4	-2.10372	0.855231
NM_001106840	300850	Gsta4	-2.10579	0.904715
NM_022707	64672	Pln	-2.10592	0.844577
NM_001109481	685826	Rprml	-2.10814	0.826986
NM_001107052	303559	Arl4d	-2.11153	0.837427
NM_017060	24913	Pla2g16	-2.11479	0.91682
NM_213626	305967	Kif13b	-2.11819	0.897246
NM_022266	64032	Ctgf	-2.12358	0.887088
NM_001108374	361032	RGD1310110	-2.13343	0.828508
NM_001007722	360504	Hba2	-2.13482	0.916643
NM_001108303	360652	Sdk2	-2.13918	0.825853
NM_013090	25624	Vamp1	-2.14528	0.842985
NM_031598	29692	Pla2g2a	-2.15857	0.877177
NM_001014008	306805	Aspn	-2.15901	0.831233
NM_031531	24795	Serpina3n	-2.1648	0.85155
NM_001014244	365345	Cyb5r2	-2.17637	0.90291
NM_001100722	315691	Lingo1	-2.18087	0.847338
NM_001101001	680115	Ptpla	-2.18517	0.838029
NM_001107076	303754	Rab40b	-2.18666	0.831162
NM_130741	170496	Lcn2	-2.18762	0.897105
NM_138914	192348	Fnbp1	-2.1927	0.911228
NM_057201	117549	Gpr37	-2.19666	0.858346
NM_133396	170908	Tesk2	-2.19739	0.837357
NM_019328	54278	Nr4a2	-2.19863	0.900361
NM_001013853	287167	LOC287167	-2.20342	0.859337
NM_017348	50690	Slc6a8	-2.20413	0.914413
NM_001107236	305497	Cobl	-2.20585	0.854063
NM_031345	83514	Tsc22d3	-2.20641	0.917316
NM_001077641	24654	Plcb1	-2.21227	0.859904
NM_001107899	312912	Prex2	-2.2218	0.890415
NM_001037200	313840	Rmdn2	-2.23349	0.861249
NM_012671	24827	Tgfa	-2.23406	0.906343

NM_053365	79451	Fabp4	-2.23826	0.918413
NM_053889	116669	Vwf	-2.23962	0.869319
NM_001029899	301460	Adam23	-2.24423	0.880185
NM_001011922	291044	Nedd9	-2.24519	0.886946
NM_198731	290551	Chdh	-2.24892	0.816438
NM_031648	58971	Fxyd1	-2.25856	0.921988
NM_001168527	296115	Secisbp2l	-2.25927	0.921917
NM_001281824	303638	Abca8a	-2.26555	0.921669
NM_012927	25409	Cdh6	-2.26577	0.829039
NM_001107533	308787	Adamts13	-2.27244	0.800333
NM_001127574	691431	Slc25a33	-2.2733	0.805854
NM_001013218	362835	Reep6	-2.27641	0.89353
NM_001012459	29313	Clec11a	-2.27686	0.872717
NM_001015024	361686	Osbpl5	-2.27714	0.915121
NM_177481	140915	Slco3a1	-2.28141	0.915121
NM_053375	84390	Hcn1	-2.28727	0.836826
NM_001107768	311384	Sema6d	-2.28841	0.906768
NM_001012107	313725	Gpr157	-2.29413	0.809783
NM_175582	291023	Id4	-2.29664	0.908113
NM_080882	29213	Tubb4a	-2.2993	0.920784
NM_001135878	363517	Plxnb3	-2.30344	0.903157
NM_001113752	362278	Tmem189	-2.30847	0.922306
NM_022543	64387	Ccdc80	-2.3343	0.908537
NM_138502	29254	Mgll	-2.33678	0.923014
NM_001191810	301509	Tns1	-2.34196	0.916608
NM_001109535	689377	Rab20	-2.34383	0.872292
NM_001100657	291445	Megf10	-2.34417	0.826242
NM_172033	64471	Plekhb1	-2.34462	0.92535
NM_017353	50719	Slc7a5	-2.34679	0.910555
NM_022860	64828	B4galnt1	-2.36498	0.826278
NM_012981	25482	Mras	-2.38064	0.907157
NM_017136	29230	Sqle	-2.38082	0.916926
NM_134459	171485	Cd9912	-2.39279	0.913139
NM_013060	25587	Id2	-2.39368	0.91321
NM_031333	83501	Cdh2	-2.39421	0.897883
NM_001109563	689986	LOC689986	-2.40507	0.871337
NM_053823	116548	Pcsk5	-2.41029	0.814703
NM_031590	29576	Wisp2	-2.42609	0.841463
NM_133415	170928	Necab2	-2.42716	0.812615
NM_139192	246074	Scd1	-2.42983	0.90967
NM_001037217	304301	Mmd2	-2.43133	0.87431
NM_080906	140942	Ddit4	-2.43634	0.916395
NM_001032285	406167	Prrt1	-2.44128	0.913705
NM_001191647	287478	Ctns	-2.4428	0.918024

NM_001108226	316526	Wnt6	-2.44557	0.904821
NM_012820	25288	Acsl1	-2.45186	0.914201
NM_013162	25703	Rbp4	-2.46305	0.891901
NM_001033883	24772	Cxcl12	-2.46854	0.847444
NM_001107947	313385	Kank4	-2.46871	0.916785
NM_024399	79251	Aspa	-2.47289	0.917316
NM_031623	58844	Grb14	-2.47969	0.911086
NM_013198	25750	Maob	-2.49365	0.924749
NM_001107495	308445	Cyp2s1	-2.49793	0.910484
NM_001047102	360687	Cadm2	-2.50516	0.912643
NM_001107131	304375	Agfg2	-2.50518	0.882876
NM_001165880	406166	Egfl8	-2.51363	0.919298
NM_001106610	297453	Hdac11	-2.51682	0.894556
NM_031049	81681	Lss	-2.52567	0.906838
NM_001047915	362799	Rapgef5	-2.5281	0.867372
NM_019276	50555	Ugt8	-2.53932	0.929244
NM_012543	24309	Dbp	-2.54552	0.84525
NM_001017457	314438	Degs2	-2.54706	0.805111
NM_001108644	362429	Mfap5	-2.55137	0.927651
NM_001004080	296654	Gsn	-2.55426	0.92981
NM_001006995	308100	Acat2	-2.55869	0.921811
NM_031543	25086	Cyp2e1	-2.56119	0.892397
NM_001191563	309533	Sorcs1	-2.56182	0.922023
NM_001109263	500527	Lurap1	-2.56512	0.905706
NM_001007691	308807	Prss23	-2.56574	0.923616
NM_031840	83791	Fdps	-2.56771	0.928359
NM_001191970	500032	Thsd7a	-2.57392	0.804651
NM_001024334	500300	LOC500300	-2.57573	0.916608
NM_001134604	498392	Cytl1	-2.58046	0.904715
NM_053777	116457	Mapk8ip1	-2.5835	0.929598
NM_031062	81726	Mvd	-2.59344	0.906874
NM_001013049	289734	Smtn	-2.60931	0.927085
NM_001104527	312358	Prr15	-2.61657	0.871797
NM_001108669	362540	Ptplad2	-2.62195	0.919192
NM_001011991	299923	Ndrg1	-2.6484	0.933421
NM_001191800	316023	Dclk3	-2.65685	0.890875
NM_023104	65984	Aacs	-2.65744	0.910838
NM_001012345	252900	Dgat2	-2.69805	0.922236
NM_001137644	500592	Tnfrsf25	-2.70644	0.805394
NM_022533	64364	Pllp	-2.70694	0.934553
NM_001107869	312492	Dysf	-2.71636	0.878628
NM_001033923	500112	Gimap8	-2.71919	0.888185
NM_001163168	25054	Ntrk2	-2.71967	0.924678
NM_001108162	315762	Rasl12	-2.72015	0.901458

NM_182954	360202	Ston1	-2.73702	0.926448
NM_001109547	689711	Fam101a	-2.74711	0.897317
NM_001008520	313917	Abhd1	-2.75626	0.912219
NM_031766	83575	Cpz	-2.76196	0.900644
NM_012548	24323	Edn1	-2.77004	0.891548
NM_053536	85497	Klf15	-2.77564	0.906308
NM_001100882	290562	Sema3g	-2.77663	0.901564
NM_017308	29744	Sema6c	-2.78172	0.924466
NM_001271143	306792	S1pr3	-2.78175	0.930483
NM_001013858	287472	Tlcd1	-2.80511	0.896114
NM_001105833	287644	Phospho1	-2.81398	0.909882
NM_145095	246325	Kcnh8	-2.81562	0.804191
NM_022608	64543	Sec14l3	-2.81587	0.897317
NM_021595	59115	Ninj2	-2.81614	0.911581
NM_001109597	690489	Cys1	-2.82978	0.882168
NM_001109430	680883	Lrtm2	-2.83255	0.805147
NM_019361	54323	Arc	-2.83767	0.843834
NM_144741	246250	Retn	-2.83949	0.872186
NM_031556	25404	Cav1	-2.84533	0.853037
NM_001201369	292077	Sult5a1	-2.84881	0.889034
NM_053856	116635	Scg3	-2.85385	0.893353
NM_053715	114507	Slc5a3	-2.87472	0.840259
NM_001107942	313325	Lad1	-2.87793	0.843728
NM_031602	29718	Kcnj10	-2.88376	0.812969
NM_001106104	291022	Ptpdc1	-2.8951	0.923793
NM_153621	266729	Dab1	-2.898	0.86408
NM_001107793	311569	Acss2	-2.89962	0.928076
NM_001109426	680723	Tmem88b	-2.90545	0.903511
NM_144737	246245	Fmo2	-2.90963	0.90068
NM_001109423	680615	Hist1h2ak	-2.9165	0.840082
NM_173094	24450	Hmgcs2	-2.9189	0.813925
NM_001106893	301264	Cyp39a1	-2.94289	0.865461
NM_052809	81718	Cdo1	-2.94319	0.927191
NM_001108970	366352	Mob3b	-2.94589	0.927333
NM_001106081	678743	Tll1	-2.94734	0.888326
NM_031688	64347	Sncg	-2.95063	0.941562
NM_001108578	362107	Aif1l	-2.95272	0.935757
NM_001107239	305509	Adcy1	-2.9649	0.909068
NM_053642	114100	Sc5d	-2.97376	0.937633
NM_012517	24239	Cacna1c	-2.98145	0.877141
NM_017137	29232	Clcn2	-2.98313	0.927226
NM_203409	288280	Ncam2	-2.99696	0.831941
NM_001282336	100912292	LOC100912292	-3.0021	0.874912
NM_031715	65152	Pfkm	-3.01973	0.938093

NM_001108354	360916	Tmem150c	-3.02211	0.810562
NM_017235	29540	Hsd17b7	-3.0231	0.846701
NM_133295	113902	Ces1d	-3.02936	0.853851
NM_012941	25427	Cyp51	-3.04027	0.942765
NM_012881	25353	Spp1	-3.04559	0.9445
NM_001025129	361018	Fam107a	-3.05121	0.922094
NM_001012054	307100	Calml3	-3.0642	0.834348
NM_053503	85265	Ajuba	-3.06719	0.916926
NM_182738	308965	Chp2	-3.06947	0.929881
NM_053633	114090	Egr2	-3.09356	0.939226
NM_031834	83783	Sult1a1	-3.10847	0.941774
NM_080886	140910	Msmo1	-3.11081	0.944818
NM_023023	65208	Dpysl5	-3.11445	0.851338
NM_133567	171097	Adap1	-3.15489	0.911688
NM_001107811	311730	Gpr155	-3.1811	0.908891
NM_153311	266681	Tmprss5	-3.1894	0.937208
NM_001010958	314441	Slc25a29	-3.19198	0.892468
NM_031521	24586	Ncam1	-3.19261	0.937916
NM_001111341	690102	Hist2h2ab	-3.2208	0.943721
NM_001009399	309262	Nsdhl	-3.22473	0.944641
NM_144744	246253	Adipoq	-3.25268	0.934447
NM_001135007	680451	Nrbp2	-3.25271	0.948393
NM_012945	25433	Hbegf	-3.25415	0.944393
NM_019363	54349	Aox1	-3.26187	0.917209
NM_001191678	305332	Limch1	-3.29266	0.947756
NM_001012049	306424	Mfap3l	-3.2945	0.940429
NM_001025688	310811	Palmd	-3.30407	0.939933
NM_030852	81510	Mia	-3.30886	0.893176
NM_001100974	307398	Arhgef37	-3.3139	0.861709
NM_001080148	298298	Dhcr24	-3.31979	0.94903
NM_053500	85262	Slc25a27	-3.34075	0.803235
NM_012505	24212	Atp1a2	-3.34135	0.948039
NM_012703	25357	Thrsp	-3.34883	0.911688
NM_021690	59326	Rapgef3	-3.35255	0.919935
NM_001108218	316395	Hecw2	-3.35688	0.823835
NM_012497	24191	Aldoc	-3.35802	0.946092
NM_057116	117256	Ppp2r2c	-3.38084	0.937279
NM_199502	363455	Chrdl1	-3.40422	0.913068
NM_199498	360626	Krt19	-3.40698	0.861886
NM_001034944	366962	Grap2	-3.41437	0.838702
NM_013134	25675	Hmgcr	-3.42391	0.950021
NM_013094	25629	Plin1	-3.43615	0.892822
NM_001107065	303678	Caskin2	-3.44282	0.950658
NM_012598	24539	Lpl	-3.4444	0.9468

NM_012972	25470	Kcna5	-3.4539	0.927297
NM_001108526	361764	Sema4g	-3.46534	0.943154
NM_001107056	303601	Cyb561	-3.51183	0.916501
NM_024375	79216	Gdf10	-3.52137	0.816048
NM_001106662	298141	Frmd3	-3.52743	0.930943
NM_017070	24950	Srd5a1	-3.53098	0.950977
NM_175762	300438	Ldlr	-3.54245	0.944783
NM_001134746	500219	Lrrtm4	-3.5444	0.906661
NM_017255	29597	P2ry2	-3.55069	0.951649
NM_001048042	289095	Nmnat2	-3.55322	0.89176
NM_001107857	312275	Ephb6	-3.55661	0.931899
NM_019292	54232	Car3	-3.562	0.954729
NM_053621	113970	Magi2	-3.58127	0.922023
NM_001130502	315145	Fam83f	-3.58309	0.939615
NM_053767	114767	Ptpre	-3.60463	0.948039
NM_130431	161476	Hspb2	-3.60783	0.950658
NM_001163921	685202	Efcc1	-3.62025	0.933633
NM_001191072	680616	Ppp1r16b	-3.62315	0.936996
NM_001037336	641521	Lrrc4	-3.64373	0.909245
NM_022008	63848	Fxyd7	-3.65067	0.957844
NM_031634	58923	Mefv	-3.68557	0.807978
NM_031066	81730	Fez1	-3.68566	0.955189
NM_213624	291325	St8sia6	-3.72166	0.897883
NM_001105749	116996	Il16	-3.7254	0.946163
NM_212522	314251	Sptb	-3.75779	0.943615
NM_012808	25274	Tst	-3.76784	0.938765
NM_053681	114216	S100a3	-3.77102	0.920643
NM_031003	81632	Abat	-3.77714	0.90383
NM_053535	85496	Enpp1	-3.78594	0.94142
NM_001107713	310764	Fam212b	-3.79946	0.958516
NM_001107645	309922	Rhobtb3	-3.8163	0.958445
NM_019169	29219	Snca	-3.89618	0.965914
NM_001108438	361364	Dok4	-3.89754	0.952499
NM_001107659	310207	Sema5a	-3.90748	0.96471
NM_001004236	298436	Tspan1	-3.93369	0.816296
NM_053686	114246	Trpv6	-3.94306	0.804439
NM_001130548	314981	Col14a1	-3.94523	0.964498
NM_145717	29639	Fxyd2	-3.94992	0.941986
NM_183403	29326	Gpx2	-3.97661	0.948074
NM_001108584	362161	Rapsn	-3.98142	0.91275
NM_022215	60666	Gpd1	-3.9926	0.962728
NM_012946	25434	Sparcl1	-4.00069	0.96871
NM_022392	64194	Insig1	-4.02143	0.963507
NM_012935	25420	Cryab	-4.02974	0.969453

NM_017027	24564	Mpz	-4.04019	0.969807
NM_022182	29348	Fgf7	-4.04987	0.963613
NM_053457	84588	Cldn11	-4.06514	0.867868
NM_031154	81869	Gstm7	-4.0678	0.959684
NM_017251	29584	Gjb1	-4.10969	0.966693
NM_001009632	289388	G0s2	-4.15231	0.914519
NM_001127567	500841	RGD1561648	-4.17648	0.867195
NM_138521	360616	Ppp1r1b	-4.18376	0.958091
NM_001191077	681021	Paqr6	-4.21659	0.971967
NM_001135834	288271	Mrap	-4.2265	0.964498
NM_001100713	313644	Rap1gap	-4.25636	0.958339
NM_001106784	299802	Lgr5	-4.26375	0.956605
NM_001106976	302934	Ppl	-4.27575	0.874558
NM_198780	362282	Pck1	-4.33659	0.887406
NM_001191575	116680	Ptpru	-4.34555	0.80504
NM_001271084	499157	Svip	-4.38365	0.975825
NM_001002835	314280	Smoc1	-4.39402	0.952782
NM_053346	83834	Nrn1	-4.39785	0.955224
NM_001107591	309377	Sfrp5	-4.46578	0.977984
NM_153294	259224	Npw	-4.51885	0.900963
NM_001013071	293688	Tm7sf2	-4.56145	0.955331
NM_001105754	192247	Sez6	-4.61703	0.807164
NM_053539	89784	Idi1	-4.6305	0.97855
NM_053955	117024	Crym	-4.67319	0.819305
NM_019278	50561	Resp18	-4.6803	0.941703
NM_012561	24373	Fst	-4.70118	0.875088
NM_001271202	303659	Kif19	-4.7728	0.978125
NM_017268	29637	Hmgcs1	-4.7868	0.983081
NM_134383	171402	Elovl6	-4.79536	0.973347
NM_138530	171564	Pbld1	-4.83049	0.942022
NM_019238	29580	Fdft1	-4.83661	0.979506
NM_001105994	289440	Ephx4	-4.87299	0.940181
NM_145670	246755	Bcas1	-4.9214	0.982621
NM_012686	24877	Vsnl1	-4.92654	0.953101
NM_001170399	304280	Tmem130	-4.92731	0.825251
NM_001108901	365038	Samd5	-4.9537	0.812155
NM_001047103	360882	Cadm3	-4.9553	0.985523
NM_012970	25468	Kcna2	-5.01943	0.964463
NM_001109394	680178	Cldn20	-5.02141	0.885636
NM_001107745	311184	C1qtnf4	-5.06059	0.852081
NM_053504	85266	Prss12	-5.0668	0.976922
NM_001108088	314772	Epyc	-5.06814	0.941916
NM_080692	140725	Cacng4	-5.07428	0.945845
NM_001109403	680404	C1ql3	-5.07751	0.974621

NM_001109467	685462	Emid1	-5.09063	0.978515
NM_145769	252892	Lgi1	-5.13868	0.964144
NM_001108491	361602	Me3	-5.14104	0.905423
NM_031686	64155	Scn7a	-5.17037	0.988213
NM_153737	266803	Sostdc1	-5.1811	0.987541
NM_173152	286937	Pex5l	-5.18726	0.92489
NM_001109294	501002	RGD1562084	-5.19106	0.818208
NM_001105894	288289	Chodl	-5.25729	0.92804
NM_001037327	364634	Csmd1	-5.30514	0.869248
NM_021688	59324	Kcnk1	-5.3127	0.982656
NM_001134610	499126	Igflr1	-5.34399	0.88776
NM_031334	83502	Cdh1	-5.41219	0.986302
NM_053514	85327	Lin7a	-5.47044	0.914236
NM_001005898	450224	Omg	-5.48558	0.856152
NM_031743	84550	Slc24a2	-5.61245	0.883442
NM_017037	24660	Pmp22	-5.62048	0.993239
NM_001109051	498034	Fn3k	-5.67093	0.863373
NM_001109480	685756	Tmem229a	-5.6805	0.974161
NM_001170597	362931	Bai1	-5.68305	0.936075
NM_013166	25707	Cntf	-5.7341	0.993098
NM_001109514	688790	Pmp2	-5.77245	0.993947
NM_053440	84510	Stmn2	-5.86483	0.88238
NM_001191844	361895	Elovl7	-5.87359	0.986868
NM_001008514	298487	Cldn19	-5.93563	0.994195
NM_001127504	499156	Gas2	-6.03341	0.956322
NM_030856	81514	Lrrn3	-6.06874	0.990656
NM_001127538	307925	Spire2	-6.10691	0.95887
NM_001107283	306147	Slitrk1	-6.19025	0.807872
NM_001135253	689933	Syce3	-6.32346	0.912573
NM_012846	25317	Fgf1	-6.33169	0.990372
NM_012798	25263	Mal	-6.41078	0.996567
NM_001109492	686081	B3galt2	-6.46016	0.910378
NM_001012738	29593	Ckmt1b	-6.5645	0.840648
NM_001135583	307855	Fa2h	-6.5684	0.995894
NM_001012009	303272	Pipox	-6.574	0.823588
NM_017226	29511	Padi2	-6.67441	0.990549
NM_024394	79246	Htr3a	-6.78589	0.83456
NM_053968	117038	Mt3	-6.82392	0.996106
NM_213628	364901	St8sia5	-6.93092	0.968108
NM_173095	24520	Kcna1	-7.01968	0.994018
NM_181382	315675	Gldn	-7.06694	0.995222
NM_001169141	306616	Myom2	-7.0799	0.956534
NM_023971	66027	Drp2	-7.25973	0.997487
NM_012929	25412	Col2a1	-7.27484	0.982727

NM_019375	56003	3-Sep	-7.31942	0.990479
NM_144756	246274	Faim2	-7.52314	0.983612
NM_012654	24784	Slc9a3	-7.53399	0.943402
NM_023976	78960	Prx	-7.55346	0.998903
NM_001106110	291130	Dcdc2	-7.62037	0.89314
NM_001253918	689826	Ncmap	-7.6476	0.99908
NM_139339	246235	Slc36a2	-7.82387	0.99508
NM_024346	29246	Stmn3	-7.88634	0.954764
NM_001134845	688613	LOC688613	-7.9667	0.984815
NM_030993	25113	Ddn	-8.45194	0.986797
NM_181636	353303	Col23a1	-8.54854	0.99147
NM_030854	81512	Lect1	-8.57293	0.998053
NM_013015	25526	Ptgds	-8.6928	0.807376
NM_001008311	295619	Ermn	-8.92122	0.833322
NM_022866	64846	Slc13a3	-9.61077	0.98347
NM_017190	29409	Mag	-9.6675	0.996779
NM_181433	305427	Otop1	-10.2266	0.934766
NM_001135710	287060	Sec1415	-10.3593	0.94004
NM_053809	116499	Fgf4	-10.4265	0.943862
NM_001109374	679668	LRRTM1	-10.4469	0.94457
NM_022285	64057	Hapln2	-11.539	0.975117

In this table up-regulated genes are highlighted in red and the down-regulated genes are highlighted in green.

Table S11. DEGs between normal motor nerves and normal sensory nerves

RNA_nucleotide_accession	GeneID	GeneSymbol	log2Ratio	Probability
NM_031531	24795	Serpina3n	2.386093	0.863743
NM_001105894	288289	Chodl	1.550571	0.808416
NM_001282336	100912292	LOC100912292	1.509071	0.806605
NM_022849	170568	Dmbt1	1.485806	0.815518
NM_021595	59115	Ninj2	1.344347	0.847088
NM_001007691	308807	Prss23	1.293707	0.865909
NM_053856	116635	Scg3	1.2781	0.814311
NM_001127565	500464	Epb4114b	1.262052	0.857848
NM_001106225	292597	Ttyh1	1.252637	0.855078
NM_001034124	287382	Mfap4	1.23718	0.800071
NM_031333	83501	Cdh2	1.173121	0.825355
NM_001002805	406161	C4b	1.156009	0.81907
NM_031521	24586	Ncam1	1.127729	0.842756
NM_022008	63848	Fxyd7	1.100414	0.848402
NM_031765	83574	Rxrg	1.08142	0.823438
NM_001163168	25054	Ntrk2	1.039391	0.83402
NM_031686	64155	Scn7a	1.012866	0.838104

NM_001047102	360687	Cadm2	1.008125	0.816442
NM_134411	171452	Rab3il1	-1.0034	0.805291
NM_001134696	100188934	Ctxn3	-1.01528	0.821911
NM_013104	25641	Igfbp6	-1.03575	0.849112
NM_001108621	362336	Fam180a	-1.0366	0.812322
NM_012880	25352	Sod3	-1.0409	0.847266
NM_199396	315259	Prickle1	-1.05354	0.813175
NM_001007008	361824	Chchd10	-1.05757	0.833523
NM_001012118	315039	Osr2	-1.06185	0.813991
NM_001107749	311209	Tp53il1	-1.06741	0.824858
NM_031118	81782	Soat1	-1.08663	0.829119
NM_001105797	287450	Slc16a11	-1.12071	0.841655
NM_012881	25353	Spp1	-1.12309	0.856392
NM_024127	25112	Gadd45a	-1.1262	0.819389
NM_020100	56820	Ramp3	-1.13532	0.81005
NM_053021	24854	Clu	-1.13914	0.857315
NM_001108173	315870	Tbx18	-1.16311	0.801669
NM_031817	83717	Omd	-1.17429	0.839986
NM_001001515	361084	Lmo7	-1.17778	0.830575
NM_012929	25412	Col2a1	-1.18052	0.852024
NM_001012091	311547	Foxs1	-1.18516	0.840518
NM_001109514	688790	Pmp2	-1.18822	0.861896
NM_001108164	315795	Cgnl1	-1.1984	0.837749
NM_020088	117242	Tenm2	-1.21061	0.820916
NM_030859	81517	Mdk	-1.22067	0.831072
NM_001108374	361032	RGD1310110	-1.2355	0.823011
NM_172035	64512	Fzd2	-1.26366	0.861257
NM_001108045	314332	Tmem63c	-1.27351	0.800959
NM_001108870	364405	Scara3	-1.29448	0.864915
NM_001034927	311642	Sulf2	-1.3194	0.869815
NM_001126300	686539	Islr	-1.32251	0.874609
NM_001101680	171356	Foxc2	-1.34149	0.846591
NM_133623	171163	Slc6a13	-1.38638	0.850355
NM_001106666	298189	Bnc2	-1.39456	0.829119
NM_053861	116640	Tnc	-1.40108	0.833629
NM_001106306	293566	Cpxm2	-1.41467	0.874325
NM_053394	84410	Klf5	-1.42525	0.871626
NM_019212	29437	Acta1	-1.45744	0.835795
NM_001107495	308445	Cyp2s1	-1.47718	0.882528
NM_001107942	313325	Lad1	-1.50637	0.851243
NM_031699	65129	Cldn1	-1.51592	0.885227
NM_001110143	306454	Cldn22	-1.51706	0.848757
NM_181087	312495	Cyp26b1	-1.52843	0.817294
NM_001106750	299236	Flrt2	-1.5351	0.822017

NM_001047957	500350	LOC500350	-1.5529	0.863885
NM_053566	89830	Ptch1	-1.55367	0.884304
NM_017184	29388	Tnni1	-1.57142	0.818359
NM_001104633	246262	Sema3d	-1.59687	0.839631
NM_001012092	311549	Xkr7	-1.5975	0.839631
NM_001169141	306616	Myom2	-1.64109	0.882422
NM_013107	25644	Bmp6	-1.66061	0.821804
NM_053608	94341	Kcnj13	-1.67261	0.856286
NM_001024276	366792	Mettl7b	-1.67463	0.852024
NM_017074	24962	Cth	-1.6918	0.808452
NM_001004099	394266	Gjb2	-1.75593	0.856925
NM_053731	114523	Ntn1	-1.768	0.882173
NM_183403	29326	Gpx2	-1.77502	0.897514
NM_134376	171393	Clstn3	-1.80704	0.847266
NM_031069	81733	Nell1	-1.82005	0.835405
NM_001005898	450224	Omg	-1.91301	0.867969
NM_031783	83613	Nefl	-2.00884	0.882386
NM_153300	266603	Aldh1a3	-2.01352	0.82777
NM_001025627	288016	Leprel1	-2.01967	0.863991
NM_017221	29499	Shh	-2.16079	0.902841
NM_017244	29563	Crabp2	-2.36626	0.902024
NM_031813	83708	Mybph	-2.42383	0.8663
NM_199498	360626	Krt19	-2.45834	0.91005
NM_017069	24947	Gabra3	-2.47516	0.883665
NM_001007601	29700	Pcbd1	-2.53786	0.9038
NM_012676	24837	Tnnt2	-2.60243	0.90625
NM_001107583	309312	Gldc	-2.68093	0.885298
NM_199370	25626	Krt8	-2.83471	0.894815
NM_001100842	305104	Col9a1	-4.28502	0.945632
NM_001047870	300242	Krt7	-5.66761	0.94581
NM_001008809	406227	Krt72	-6.29057	0.816548
NM_001013943	299052	Aldoart2	-6.50308	0.838068
NM_019183	29275	Actc1	-8.79141	0.828977
NM_001004022	287700	Krt15	-9.93233	0.925746

In this table up-regulated genes are highlighted in red and the down-regulated genes are highlighted in green.

Table S12. DEGs between injured motor nerves and injured sensory nerves

RNA_nucleotide_accession	GeneID	GeneSymbol	log2Ratio	Probability
NM_022242	63912	Fam129a	1.166347	0.874884
NM_012608	24590	Mme	1.154421	0.838264
NM_001108353	360914	Plac8	-1.01364	0.821162

NM_001017479	497979	Tmem100	-1.02662	0.801891
NM_012881	25353	Spp1	-1.09097	0.867063
NM_153300	266603	Aldh1a3	-1.17198	0.807794
NM_019156	29169	Vtn	-1.20566	0.825606
NM_001107495	308445	Cyp2s1	-1.21705	0.810638
NM_030845	81503	Cxcl1	-1.35161	0.823686
NM_001106306	293566	Cpxm2	-1.37343	0.843526
NM_001105797	287450	Slc16a11	-1.44446	0.86532
NM_001109514	688790	Pmp2	-1.45456	0.871542
NM_012505	24212	Atp1a2	-1.4818	0.863223
NM_012932	25415	Crmp1	-1.5658	0.855614
NM_001107591	309377	Sfrp5	-1.61631	0.89291
NM_001004099	394266	Gjb2	-1.61637	0.83741
NM_033237	29141	Gal	-1.66746	0.868805

In this table up-regulated genes are highlighted in red and the down-regulated genes are highlighted in green.
